# Supplementary material for: Uncovering the cytochrome P450-catalyzed methylenedioxy bridge formation in streptovaricins biosynthesis
Source: Nat Commun. 2020 Sep 9;11:4501. doi: 10.1038/s41467-020-18336-5 (PMC7481197; doi:10.1038/s41467-020-18336-5)
Supplement: Supplementary file 1 — Supplementary information [file 41467_2020_18336_MOESM1_ESM.pdf]

Supplementary Information

Uncovering the Cytochrome P450-Catalyzed Methylenedioxy Bridge Formation in Streptovaricins Biosynthesis

Sun et al.

Table of Contents

Supplementary Methods

Supplementary Tables

Supplementary Table 1. ^{1}H and ^{13}C NMR data of compounds **1** and **2**.
Supplementary Table 2. ^{1}H (600 MHz) and ^{13}C (150 MHz) NMR data of compounds **3** and **4**.
Supplementary Table 3. ^{1}H (600 MHz) and ^{13}C (150 MHz) NMR Data of compounds **5** and **6** ($CHCl_3$-d_1).
Supplementary Table 4. Kinetic parameters of StvP2 catalyzing substrates **2**, **4** and **6** to form MDB.
Supplementary Table 5. Data collection and refinement statistics.
Supplementary Table 6. ^{1}H (600 MHz) and ^{13}C (150 MHz) NMR Data of compounds **7** (DMSO-d_6) and **8** (CH_3OH-d_4).
Supplementary Table 7. Bacterial strains used in this study.
Supplementary Table 8. Plasmids used in this study.
Supplementary Table 9. Primers used in this study.

Supplementary Figures

Supplementary Fig. 1 MDB-contained streptovaricins reported.
Supplementary Fig. 2 ESI-HRMS spectrum of streptovaricin C (**1**)
Supplementary Fig. 3 ^{1}H NMR spectrum (400 MHz, $CHCl_3$-d_1) of streptovaricin C (**1**).
Supplementary Fig. 4 ^{13}C NMR spectrum (100 MHz, $CHCl_3$-d_1) of streptovaricin C (**1**).
Supplementary Fig. 5 Phylogenetic tree of cytochrome P450 enzymes for MDB formation of natural products.
Supplementary Fig. 6 SDS-PAGE analysis of recombination proteins StvM1, StvA2 and StvP2.
Supplementary Fig. 7 *In vitro* enzymatic conversion of the crude extract from ΔstvP2 catalyzed by StvP2.
Supplementary Fig. 8 ESI-HRMS spectrum of 6-methoxy-streptovaricin C (**2**)
Supplementary Fig. 9 ^{1}H NMR spectrum (600 MHz, CH_3OH-d_4) of 6-methoxy-streptovaricin C (**2**).
Supplementary Fig. 10 ^{13}C NMR and DEPT135 spectrum (150 MHz, CH_3OH-d_4) of 6-methoxy-streptovaricin C (**2**).
Supplementary Fig. 11 HSQC spectrum (600 MHz, CH_3OH-d_4) of 6-methoxy-streptovaricin C (**2**).
Supplementary Fig. 12 ^{1}H-^{1}H COSY spectrum (600 MHz, CH_3OH-d_4) of 6-methoxy-streptovaricin C (**2**).
Supplementary Fig. 13 HMBC spectrum (600 MHz, CH_3OH-d_4) of 6-methoxy-streptovaricin C (**2**).
Supplementary Fig. 14 Crucial HMBC correlations of streptovaricin derivatives **2-8**.
Supplementary Fig. 15 Scheme of mutants ΔstvP1P5 and ΔstvP1P5P2 construction and verification.

Supplementary Fig. 16 Scheme of mutants ΔstvP1P4 and ΔstvP1P4P2 construction and verification.
Supplementary Fig. 17 ESI-HRMS spectrum of streptovaricin H (**3**)
Supplementary Fig. 18 ^{1}H NMR spectrum (600 MHz, $CHCl_3$-d_1) of streptovaricin H (**3**).
Supplementary Fig. 19 ^{13}C NMR and DEPT135 spectrum (150 MHz, $CHCl_3$-d_1) of streptovaricin H (**3**).
Supplementary Fig. 20 HSQC spectrum (600 MHz, $CHCl_3$-d_1) of streptovaricin H (**3**).
Supplementary Fig. 21 ^{1}H-^{1}H COSY spectrum (600 MHz, $CHCl_3$-d_1) of streptovaricin H (**3**).
Supplementary Fig. 22 HMBC spectrum (600 MHz, $CHCl_3$-d_1) of streptovaricin H (**3**).
Supplementary Fig. 23 ESI-HRMS spectrum of 6-methoxy-streptovaricin H (**4**)
Supplementary Fig. 24 ^{1}H NMR spectrum (600 MHz, CH_3OH-d_4) of 6-methoxy-streptovaricin H (**4**).
Supplementary Fig. 25 ^{13}C NMR and DEPT135 spectrum (150 MHz, CH_3OH-d_4) of 6-methoxy-streptovaricin H (**4**).
Supplementary Fig. 26 HSQC spectrum (600 MHz, CH_3OH-d_4) of 6-methoxy-streptovaricin H (**4**).
Supplementary Fig. 27 ^{1}H-^{1}H COSY spectrum (600 MHz, CH_3OH-d_4) of 6-methoxy-streptovaricin H (**4**).
Supplementary Fig. 28 HMBC spectrum (600 MHz, CH_3OH-d_4) of 6-methoxy-streptovaricin H (**4**).
Supplementary Fig. 29 ESI-HRMS spectrum of streptovaricin D (**5**)
Supplementary Fig. 30 ^{1}H NMR spectrum (600 MHz, $CHCl_3$-d_1) of streptovaricin D (**5**).
Supplementary Fig. 31 ^{13}C NMR and DEPT135 spectrum (150 MHz, $CHCl_3$-d_1) of streptovaricin D (**5**).
Supplementary Fig. 32 HSQC spectrum (600 MHz, $CHCl_3$-d_1) of streptovaricin D (**5**).
Supplementary Fig. 33 ^{1}H-^{1}H COSY spectrum (600 MHz, $CHCl_3$-d_1) of streptovaricin D (**5**).
Supplementary Fig. 34 HMBC spectrum (600 MHz, $CHCl_3$-d_1) of streptovaricin D (**5**).
Supplementary Fig. 35 ESI-HRMS spectrum of 6-methoxy-streptovaricin D (**6**)
Supplementary Fig. 36 ^{1}H NMR spectrum (600 MHz, CH_3OH-d_4) of 6-methoxy-streptovaricin D (**6**).
Supplementary Fig. 37 ^{13}C NMR and DEPT135 spectrum (150 MHz, CH_3OH-d_4) of 6-methoxy-streptovaricin D (**6**).
Supplementary Fig. 38 HSQC spectrum (600 MHz, CH_3OH-d_4) of 6-methoxy-streptovaricin D (**6**).
Supplementary Fig. 39 ^{1}H-^{1}H COSY spectrum (600 MHz, CH_3OH-d_4) of 6-methoxy-streptovaricin D (**6**).
Supplementary Fig. 40 HMBC spectrum (600 MHz, CH_3OH-d_4) of 6-methoxy-streptovaricin D (**6**).
Supplementary Fig. 41 *In vitro* enzymatic conversion of 6-methoxy-streptovaricin H (**4**) to form streptovaricin H (**3**) catalyzed by StvP2.
Supplementary Fig. 42 *In vitro* enzymatic conversion of 6-methoxy-streptovaricin D (**6**) to form streptovaricin D (**5**) catalyzed by StvP2.

Supplementary Fig. 43 Stereo view of the StvP2 and substrate **2**-bound StvP2 structures.
Supplementary Fig. 44 Electron density map of substrate **2**.
Supplementary Fig. 45 Amino acid sequence alignment of StvP2 with other cytochrome P450 enzymes reported to catalyze MDB formation.
Supplementary Fig. 46 Scheme of mutant ΔstvM1 construction and verification.
Supplementary Fig. 47 ESI-HRMS spectrum of damavaricin C (**7**)
Supplementary Fig. 48 ^{1}H NMR spectrum (600 MHz, DMSO-d_6) of damavaricin C (**7**).
Supplementary Fig. 49 ^{13}C NMR and DEPT135 spectrum (150 MHz, DMSO-d_6) of damavaricin C (**7**).
Supplementary Fig. 50 HSQC spectrum (600 MHz, DMSO-d_6) of damavaricin C (**7**).
Supplementary Fig. 51 ^{1}H-^{1}H COSY spectrum (600 MHz, DMSO-d_6) of damavaricin C (**7**).
Supplementary Fig. 52 HMBC spectrum (600 MHz, DMSO-d_6) of damavaricin C (**7**).
Supplementary Fig. 53 ESI-HRMS spectrum of 6-methoxy-damavaricin C (**8**)
Supplementary Fig. 54 ^{1}H NMR spectrum (600 MHz, CH_3OH-d_4) of 6-methoxy-damavaricin C (**8**).
Supplementary Fig. 55 ^{13}C NMR spectrum (150 MHz, CH_3OH-d_4) of 6-methoxy-damavaricin C (**8**).
Supplementary Fig. 56 HSQC spectrum (600 MHz, CH_3OH-d_4) of 6-methoxy-damavaricin C (**8**).
Supplementary Fig. 57 ^{1}H-^{1}H COSY spectrum (600 MHz, CH_3OH-d_4) of 6-methoxy-damavaricin C (**8**).
Supplementary Fig. 58 HMBC spectrum (600 MHz, CH_3OH-d_4) of 6-methoxy-damavaricin C (**8**).
Supplementary Fig. 59 Scheme of mutant ΔstvA1 construction and verification.
Supplementary Fig. 60 Scheme of mutant ΔstvA2 construction and verification.
Supplementary Fig. 61 *In vitro* conversions of compound **8.**
Supplementary Fig. 62 LC-ESI-HRMS analysis of acetylated products of enzymatic conversion of **7** by StvP2.
Supplementary Fig. 63 A biosynthetic network of acetylation and MDB formation confirmed by *in vitro* assay.
Supplementary Fig. 64 Comparative analysis of the catalytic efficiency of StvP2 and StvA2.

Supplementary References

Supplementary Methods

Construction of double in-frame deletion mutants ΔstvP1P4 and ΔstvP1P5

To construct double in-frame deletion mutants ΔstvP1P4 and ΔstvP1P5, *stvP4*-disrupted plasmid pWHU2801 and *stvP5*-disrupted plasmid pWHU2802 (Supplementary Table 8) was introduced into in-frame deletion mutant ΔstvP1 by conjugation on ABB13 solid plates (containing 10 mM $MgCl_2$), respectively. After incubated at 28°C for 12 h, the plates were overlaid with apramycin (35 μg mL^{-1}) and nalidixic acid (30 μg mL^{-1}) and then continued to culture at 28°C for 5-6 days till the ex-conjugants formed. Single apramycin-resistant exconjugants from this plate were patched on ABB13 solid medium plate containing apramycin (35 μg mL^{-1}) and nalidixic acid (30 μg mL^{-1}), and grown at 28°C for 2 or 3 days for further resistance confirmation. To screen the double cross-over mutant, each single colony from the plate without any antibiotics was patched onto ABB13 plates without and with 35 μg mL^{-1} apramycin, respectively. Genomic DNA of single apramycin-sensitive colonies as mutant candidate was extracted and checked by PCR using the checking primers CK-stvP4-For and CK-stvP4-Rev, and CK-stvP5-For and CK-stvP5-Rev (Supplementary Table 9), and further verified by sequencing of PCR products.

Construction of triple gene in-frame deletion mutants ΔstvP1P4P2 and ΔstvP1P5P2

Firstly, the plasmid used for in-frame deletion of *stvP2* based on the double in-frame deletion mutants ΔstvP1P4 and ΔstvP1P5 was constructed. To achieve it, two DNA fragments served as upstream and downstream homologous arms flanking *stvP2* were amplified from the genome DNA of ΔstvP1 mutant using primer pairs of stvP2'-L-For, and stvP2'-L-Rev, and stvP2'-R-For and stvP2'-R-Rev (Supplementary Table 9), respectively. The PCR products were purified and then cloned into the *Streptomyces*-*E. coli* shuttle vector pYH7 by Gibson Assembly methods to obtain the plasmid pWHU2907 (Supplementary Table 8), which was verified by restriction endonuclease digestion and sequencing.

To construct triple gene in-frame deletion mutants ΔstvP1P4P2 and ΔstvP1P5P2, the plasmid pWHU2907 was introduced into ΔstvP1P4 and ΔstvP1P5 by conjugation, respectively. The rest processes of mutant construction were the same as the

construction of ΔstvP1P4 and ΔstvP1P5 mentioned above. The mutant was verified through PCR using the check primers CK-stvP2-For and CK-stvP2-Rev (Supplementary Table 9) and sequencing.

Construction of the mutant ΔstvM1

To construct the plasmid used for in-frame deletion of *stvM1*, two DNA fragments flanking *stvM1* were amplified from the genome DNA of wild-type strain then double digested by *Nde*I +*Pst*I, and *Pst*I+*Hin*dIII, respectively, and finally cloned into the *Streptomyces-E. coli* shuttle vector pYH7 to obtain the plasmid pWHU2820 (Supplementary Table 8), which was verified by restriction endonuclease digestion and sequencing.

To construct the *stvM1* in-frame deletion mutant, the plasmid pWHU2820 was introduced into wild-type strain by conjugation. The rest constructed processes was the same as the construction of ΔstvP1P4 and ΔstvP1P5 mentioned above. The mutant was verified through PCR using the check primers CK-stvM1-For and CK-stvM1-Rev (Supplementary Table 9) and sequencing.

Construction of the mutant ΔstvA1 and ΔstvA2

To construct the plasmid used for single in-frame deletion of *stvA1* and *stvA2*, two pairs of DNA fragments flanking *stvA1* and *stvA2* were amplified from the genome DNA of wild-type strain, respectively. Each pair of the DNA fragments were double digested by *Nde*I+*Eco*RI, and *Eco*RI+*Hin*dIII for *stvA1*, and *Nde*I+*Pst*I, and *Pst*I+*Hin*dIII for *stvA2*, respectively, then cloned into the *Streptomyces-E. coli* shuttle vector pYH7 to obtain the plasmid pWHU2905 and pWHU2906 (Supplementary Table 8), which were verified by restriction endonuclease digestion and sequencing.

The plasmid pWHU2905 and pWHU2906 was introduced into the wild-type strain by conjugation, respectively. The rest constructed processes was the same as the construction of ΔstvP1P4 and ΔstvP1P5 mentioned above. The mutant was verified through PCR using the check primers CK-stvA1-For and CK-stvA1-Rev for ΔstvA1, and CK-stvA2-For and CK-stvA2-Rev for ΔstvA2 (Supplementary Table 9) and sequencing.

Fermentation of the wild-type strain and isolation of streptovaricin C (1)

A patch (~1 × 1 cm^2) of wild-type strain culture on ABB13 solid medium plate was inoculated into 50 mL of TSBY liquid broth, and cultured at 28°C and 220 rpm for 36 h to obtain the seed culture. One percent of seed culture was inoculated into 10 L of SFM broth to ferment at 28°C for 5 days at 220 rpm. Then the fermentation culture was centrifuged at 5,000 rpm for 10 min and the supernatant was extracted with identical volume of ethyl acetate for three times. The combined ethyl acetate layer was concentrated under reduced pressure to yield the crude extract. Subsequently, it was subjected to a RP-18 silica gel column (15 × 310 mm) on medium pressure liquid chromatography (MPLC) system equipped with a UV detector, and eluted with acetonitrile-water (10-100%) to fractionate to 10 fractions. Based on the HPLC-DAD analysis, fractions contained the target component were further isolated and purified by semi-preparative HPLC with an Agilent column (ZORBAX Eclipse XDB-C18, 5 μm, 9.4 × 250 mm) eluted with 40% acetonitrile-water to obtain compound **1** (t_R = 15.5 min).

Fermentation of ∆stvP2 and isolation of 6-methoxy-streptovaricin C (2)

A patch (~1 × 1 cm^2) of ∆stvP2 culture on ABB13 solid medium plate was inoculated into 50 mL of TSBY liquid broth in 250 mL shake flask, and cultured at 28°C and 220 rpm in shaker for 36 h to obtain the seed culture. One percent of seed culture was inoculated into 20 L of SFM broth to ferment at 28°C for 5 days at 220 rpm. Then the fermentation culture was centrifuged at 5,000 rpm for 10 min and the supernatant was extracted with identical volume of ethyl acetate for three times. The combined ethyl acetate layer was concentrated under reduced pressure to yield the crude extract of ∆stvP2. Subsequently, it was subjected to a RP-18 silica gel column (26 × 310 mm) on MPLC system equipped with a UV detector, and eluted with a gradient concentration of acetonitrile-water (10-100%) to fractionate to 14 fractions. Based on the HPLC-DAD and LC-ESI-HRMS analysis, fractions contained the target component were further isolated and purified by semi-preparative HPLC with an Agilent column (ZORBAX Eclipse XDB-C18, 5 μm, 9.4 × 250 mm) eluted with 35% acetonitrile-water (with 1‰ v/v formic acid in water) to yield compound **2** (t_R = 14.0 min).

Fermentation of ΔstvP1P5 and ΔstvP1P4 and isolation of streptovaricin H (3) and streptovaricin D (5)

The mutants ΔstvP1P5 and ΔstvP1P4 was fermented on the same conditions as ΔstvP2. After centrifugation, the supernatant was concentrated under reduced pressure at 35°C to yield the crude extract of ΔstvP1P5 and ΔstvP1P4, respectively. Then each of the crude extract was subjected to a RP-18 column (15 × 310 mm) on MPLC system equipped with UV detector, and eluted with acetonitrile-water in a gradient concentration (10%-100%) to give 12 fractions. According to their HPLC-DAD analysis and the UV absorption feature, the fraction containing target component from ΔstvP1P5 crude extract, was further purified by HPLC on a Agilent semi-preparative column (ZORBAX Eclipse XDB-C18, 5 μm, 9.4 × 250 mm) eluted with 75% acetonitrile-water to obtain compound **3** (t_R = 19.0 min), and the fraction containing target component from ΔstvP1P4 crude extract was purified by HPLC on a Agilent semi-preparative column (ZORBAX Eclipse XDB-C18, 5 μm, 9.4 × 250 mm) eluted with 70% acetonitrile-water to yield compound **5** (t_R = 17.5 min).

Fermentation of ΔstvP1P5P2 and ΔstvP1P4P2 and isolation of 6-methoxy-streptovaricin H (4) and 6-methoxy-streptovaricin D (6)

A patch (~1 × 1 cm^2) of ΔstvP1P5P2 and ΔstvP1P4P2 cultures on a ABB13 solid medium plate was inoculated into 50 mL TSBY medium in 250 mL shake flask, respectively, and cultured at 28°C and 220 rpm in shaker for 36 h to obtain primary seed culture. Then 1% of these culture was inoculated into 1,400 mL of TSBY medium (700 mL for each in 2 L shake flask) to culture at 28°C and 220 rpm for another 36 h to obtain the secondary seed culture. Next, these cultures were inoculated into 30 L of SFM medium in 50 L fermentation tank for large-scale fermentation at 28°C and 220 rpm for 5 days with the continuous supply of sterile air. When completed, the fermentation liquid was centrifuged at 5,000 rpm for 10 min and the combined supernatant was extracted with identical volume of ethyl acetate for 3 times. The ethyl acetate layer was further concentrated under reduced pressure to yield the crude extract of ΔstvP1P5P2 and ΔstvP1P4P2. Each of them was then subjected to a RP-18 chromatography column (26 × 310 mm) on MPLC system equipped with a UV detector, eluted with acetonitrile-water (10%-100%) to give 15

fractions. According to their HPLC-DAD and LC-ESI-HRMS analysis, the target fractions were further subjected to Sephadex LH-20 gel column chromatography eluted with methanol to give 4 subfractions. Based on the detection of LC-ESI-HRMS, The target sub-fraction (calcd. *m/z* 728.36405, $[M + H]^+$) from ΔstvP1P5P2 was isolated by HPLC on a semi-preparative Agilent column (ZORBAX Eclipse XDB-C18, 5 μm, 9.4 × 250 mm) eluted with 40% acetonitrile-water (with 1‰ formic acid in water) to yield pure compound **4** (t_R = 15 min), and the target sub-fraction (calcd. *m/z* 756.35897, $[M + H]^+$) from ΔstvP1P4P2 was isolated on a semi-preparative Agilent column (ZORBAX Eclipse XDB-C18, 5 μm, 9.4 × 250 mm) eluted with 45% acetonitrile-water (with 1‰ formic acid in water) to yield pure compound **6** (t_R = 16.0 min).

Fermentation of ΔstvM1 and ΔstvA2 and isolation of damavaricin C (7) and 6-methoxy- damavaricin C (8)

The mutants ΔstvM1 and ΔstvA2 was fermented, followed by extraction with ethyl acetate on the same conditions as ΔstvP2 to obtain the crude extract of ΔstvM1 and ΔstvA2, respectively. These crude extracts were further subjected to a RP-18 chromatography column (15 × 310 mm) on MPLC system equipped with a UV detector eluted with acetonitrile-water (10%-100%) to gain 12 fractions. Detected by HPLC-DAD, the intermediate of interest, compound **7** (t_R =13.5 min), was further purified from the target fraction of ΔstvM1 crude extract by HPLC with a semi-preparative Agilent chromatography column (ZORBAX Eclipse XDB-C18, 5 μm, 9.4 × 250 mm) eluted by 40% acetonitrile-water (with 1‰ formic acid in water), and compound **8** (t_R =16.5 min) was purified from the target fraction of ΔstvA2 crude extract, by HPLC with a semi-preparative Agilent chromatography column (ZORBAX Eclipse XDB-C18, 5 μm, 9.4 × 250 mm) eluted by 45% acetonitrile-water (with 1‰ formic acid in water).

Chemical structure identification of streptovaricins (1-8)

UV/Vis spectrum of **1** exhibited the characteristic absorption with λ_{max} at 245, 262, 315, 432 nm, indicating that it was a MDB-contained streptovaricin. The ESI-HRMS spectrum of compound **1** showed a quasi-molecular ion peak at *m/z* 770.33801(calcd. for

$C_{40}H_{52}NO_{14}^+$, *m/z* 770.33823 [M + H]$^+$), indicating a molecular formula of $C_{40}H_{51}NO_{14}$, with sixteen degrees of unsaturation, as the same of streptovaricin C. Based on the same retention time with the standard streptovaricin C and comparative analysis of ^{1}H and ^{13}C NMR data with those of streptovaricin C reported[1], the structure of compound **1** was determined as streptovaricin C.

ESI-HRMS data of compound **2** exhibited a quasi-molecular ion peak at *m/z* 772.35358 (calcd. for $C_{40}H_{54}NO_{14}^+$, *m/z* 772.35388 [M + H]$^+$), in agreement with a molecular formula of $C_{40}H_{53}NO_{14}$, with fifteen degrees of unsaturation. Its UV/Vis spectrum (λ_{max} 241, 332, 345 nm) differed significantly from those of MDB-contained streptovaricins and protostreptovaricin intermediates, indicating that compound **2** owned a very unique chromophore moiety. Its ^{1}H NMR spectrum exhibited four doublet methyl signals (δ_H 0.04 (d, 6.9 Hz, 3H), 0.90 (d, 6.6 Hz, 3H), 1.28 (d, 7.2 Hz, 3H), 2.27 (d, 1.2 Hz, 3H)), seven singlet methyl signals (δ_H 1.26 (s, 3H), 2.00 (s, 3H), 2.04 (s, 3H), 2.20 (s, 3H), 2.23 (s, 3H), 3.69 (s, 3H), 3.70 (s, 3H)), of which two were singlet methoxy signals (δ_H 3.69 (s, 3H), 3.70 (s, 3H)). Comparative analysis of ^{13}C NMR and DEPT spectrum of **2** verified the presence of nine methyl carbon signals (δ_C 9.3, 10.1, 12.0, 12.5, 12.7, 18.1, 20.7, 21.9, 28.4) and two methoxy signals (δ_C 52.1, 62.6). In addition, four carbon signals (δ_C 170.5, 174.4, 174.5, 201.4) in the ^{13}C NMR spectrum suggested one keto carbonyl and three ester or amide carbonyls in the structure of **2**. A series of carbon signals in δ_C 110-160 ppm zone of ^{13}C NMR, together with the UV/Vis absorption wavelength (λ_{max} 332, 345 nm), indicated that the aromatic ring system may exist. According to the correlations in ^{1}H-^{1}H COSY, the linkage relationship from C17 to C27 was established. The key HMBC correlations from H30 (δ_H 2.04 (s, 3H)) to C15 (δ_C 174.5), C16 (δ_C 133.1), C17 (δ_C 131.1), from H25 (δ_H 3.75 (dd, 10.9, 2.5 Hz, 1H)) to C33 (δ_C 174.4), from H34 (δ_H 3.69 (s, 3H)) to C33 (δ_C 174.4), from H29 (δ_H 6.12 (d, 0.5 Hz, 1H)) to C27 (δ_C 77.3), C28 (δ_C 76.2), C36 (δ_C 28.4), C12 (δ_C 139.5) and from H37 (δ_H 2.27 (d, 1.2 Hz, 3H)) to C11 (δ_C 201.4), C12 (δ_C 139.5), C29 (δ_C 150.4) further determined the linkage of C11-C15 fragment, the same as the structure of ansa chain in streptovaricin C. Based on the analysis above, we deduced that this compound should also be naphthalene ansamycins with a different chromophore, in agreement with the rest eight degrees of unsaturation. The HMBC

correlations from H13 (δ_H 3.70 (s, 3H)) to C6 (δ_C 157.3), and from H39 (δ_H 2.20 (s, 3H)) to C38 (δ_C 170.5), C4 (δ_C 138.1) suggested that a methoxy and an acetoxy were linked to C6 and C4, respectively. Moreover, two methyl groups were connected with C3 and C7, respectively, deduced from the HMBC correlations from H14 (δ_H 2.23 (s, 3H)) to C6 (δ_C 157.3), C7 (δ_C 114.7), C8 (δ_C 155.4), and from H40 (δ_H 2.00 (s, 3H)) to C2 (δ_C 128.8), C3 (δ_C 119.1) and C4 (δ_C 138.1). Thus, the structure of compound 2 was determined as a never reported streptovaricin, with its chiral centers referred to those of streptovaricin C, and named as 6-methoxy-streptovaricin C.

The UV/Vis spectrum of compound **3** shared the same characteristic absorption peaks as that of streptovaricin C (**1**), indicating that **3** was also the MDB-contained streptovaricin. Based on the quasi-molecular ion peak of its ESI-HRMS spectrum at *m/z* 726.34772 (calcd. for $C_{39}H_{52}NO_{12}^+$, *m/z* 726.34840 $[M + H]^+$), the molecular formula of **3** was determined as $C_{39}H_{51}NO_{12}$, with fifteen degrees of unsaturation. The molecular weight (MW) of **3** was 44 amu less than that of **1**, corresponding to the loss of a carboxy anion (COO^-). Compared ^1H and ^{13}C NMR spectra with that of streptovaricin C (**1**), it was found that most of the NMR signals were almost identical but a methoxycarbonyl signals (δ_H 3.69 (s, 3H); δ_C 173.2, 52.0) are absent in **3**. HMBC correlations from H23 (δ_H 3.61 (d, 9.2 Hz, 1H)) to C24 (δ_C 32.7), C25 (δ_C 71.3), C33 (δ_C 10.7) further verified that the group linked to C24 was a methyl other than a methoxycarbonyl group. All of the NMR signals were assigned by comparative analysis of 1D and 2D NMR data. The chiral centers of 3 were assigned based on the biogenetic pathway of streptovaricins. Thus, the structure of **3** was determined and named as streptovaricin H.

The UV/Vis spectrum of **4** exhibited the same absorption characteristic as that of **2**, indicating that **4** was also a streptovaricin shared the same naphthol chromophore. ESI-HRMS spectrum exhibited a quasi-molecular ion peak at *m/z* 728.36389 (calcd. for $C_{39}H_{54}NO_{12}^+$, *m/z* 728.36405 $[M + H]^+$), suggesting that its molecular formula was $C_{39}H_{53}NO_{12}$, with fourteen degrees of unsaturation. According to correlations in the ^1H-^1H COSY spectrum as well as key HMBC correlations from H30 (δ_H 2.03 (m, 1H)) to C15 (δ_C 175.0), C16 (δ_C 132.9), C17 (δ_C 131.7), from H36 (δ_H 1.25 (s, 3H)) to C27(δ_C 77.6), C28 (δ_C 76.6), C29 (δ_C 151.0) and from H37 (δ_H 2.27 (d, 0.7 Hz, 3H)) to C29 (δ_C 151.0), C12

(δ_C 139.5), C11 (δ_C 201.4), the structure of ansa chain moiety was determined to be the same as that in compound 3. HMBC correlations from H13 (δ_H 3.70 (s, 3H)) to C6 (δ_C 157.3), and from H39 (δ_H 2.18 (s, 3H)) to C38 (δ_C 170.5), C4 (δ_C 138.0) further verified the structure of naphthalene ring fragment. The chiral centers in the structure were referred to the stereochemistry of streptovaricin C based on the biogenetic pathway. Therefore, the structure of **4** was identified umambiguously and named as 6-methoxy-streptovaricin H.

UV/Vis spectrum of compound **5** exhibited the same absorption as that of **1**, suggesting that it belonged to a MDB-contained streptovaricins. Its molecular formula was deduced to be $C_{40}H_{51}NO_{13}$, with sixteen degrees of unsaturation, on the basis of the quasi-molecular ion peak at *m/z* 754.34259 (calcd. for $C_{40}H_{52}NO_{13}^+$, *m/z* 754.34332 $[M + H]^+$) in ESI-HRMS spectrum. The MW of **5** was 16 amu less than that of **1**, corresponding to the loss of an *O* atom in the structure of **5**. HMBC correlations from H36 (δ_H 0.68 (d, 7.0 Hz, 3H)) to C27 (δ_C 73.2), C28 (δ_C 37.5), C29 (δ_C 153.4) along with correlations of H10 and H17/H7/H34 in ^1H-^1H COSY spectrum determined that C28 was a tertiary carbon connected with a methyl group, other than a quaternary carbon further substituted by a hydroxyl group as in the structure of **1**, which was in agreement with the *O* atom loss in structure. Thus, the structure of **5** was identified to be streptovaricin D[1].

According to the UV/Vis spectrum of **6**, it was inferred as a streptovaricin shared the same chromophore as that of **2**. Its molecular formula was determined to be $C_{40}H_{53}NO_{13}$, with fifteen degrees of unsaturation, in agreement with the quasi-molecular ion peak at m/z 756.35864 (calcd. for $C_{40}H_{54}NO_{13}^+$, *m/z* 756.35897 $[M + H]^+$) in the ESI-HRMS spectrum. The MW of **6** was 2 amu more than that of **5**, indicating it could be a MDB-split form of streptovaricin D. HMBC correlations from H36 (δ_H 0.58 (d, 5.6 Hz, 3H)) to C27 (δ_C 75.3), C28 (δ_C 38.1), C29 (δ_C 146.4) along with correlations of H10 and H17/H7/H34 in ^1H-^1H COSY spectrum verified that the ansa chain structure was the same as that of streptovaricin D (**5**). The stereocenters were referred to the streptovaricin D on the basis of the biogenetic pathway. Therefore, the structure of **6** was identified as 6-methoxy-streptovaricin D.

The molecular formula of compound **7** was determined as $C_{37}H_{47}NO_{13}$ from its quasi-molecular ion peak at *m/z* 714.31097 (calcd. for $C_{37}H_{48}NO_{13}^+$, 714.31202 $[M + H]^+$)

in the ESI-HRMS spectrum. HMBC correlations from H30 (δ_H 1.89 (s, 3H)) to C15 (δ_C 169.6), C16 (δ_C 131.0), C17 (δ_C 131.6), from C28-OH (δ_H 4.50 (s, 1H)) to C27 (δ_C 75.3), C28 (δ_C 74.2), C29 (δ_C 144.1), C36 (δ_C 28.8) and from H37 (δ_H 2.11 (d, 1.0 Hz, 3H)) to C11 (δ_C 196.6), C12 (δ_C 136.1), C29 (δ_C 144.1) and correlations in the ^{1}H-^{1}H COSY spectrum determined the ansa chain structure was the same as that of streptovaricin C, indicating that it could be also a streptovaricin intermediate with a different chromophore. HMBC correlations from H40 (δ_H 1.72 (s, 3H)) to C2 (δ_C 140.0), C3 (δ_C 135.3), C4 (δ_C 184.1), from C2-NH (δ_H 9.71 (s, 1H)) to C1 (δ_C 184.3), C3 (δ_C 135.3), combined with the rest eight degrees of unsaturation, determined that its main chromophore was a naphthoquinone structure. The key HMBC correlations from C8-OH (δ_H 12.56 (s, 1H)) to C1 (δ_C 184.3), C7 (δ_C 117.0), C8 (δ_C 160.9), C9 (δ_C 106.6) further indicated that a hydroxyl group was linked to C8. Thus, the structure of **7** was identified as damavaricin C[2,3], reported as an intermediate during streptovaricin biosynthesis.

The UV/Vis spectrum of **8** was very similar to that of **7**, indicating that the differences in their structures were very small. The quasi-molecular ion peak at *m/z* 728.32678 (calcd. for $C_{38}H_{50}NO_{13}^+$, 728.32767 $[M + H]^+$) in its ESI-HRMS determined their molecular formula of $C_{38}H_{49}NO_{13}$, with fifteen degrees of unsaturation. The MW of **8** was 14 amu more than that of **7**, corresponding to the substitution of a methyl group. Key HMBC correlations from C6-OCH_3 (δ_H 3.79 (s, 3H)) to C6 (δ_C 163.0) verified this deduction. All the ^{1}H and ^{13}C NMR data were assigned based on comparative analysis of 1D (^{1}H NMR, ^{13}C NMR) and 2D (^{1}H-^{1}H COSY, HSQC, HMBC) NMR spectra. So, the structure of **8** was identified as a methylated product of damavaricin C at C6-OH, and named as 6-methoxy-damavaricin C.

Sequential enzymatic conversion of 8 by StvA2 or/and StvP2

A series of biochemical conversions of **8** were performed in a 100 μL reaction system containing 2.5 μM StvA2 or/and 5 μM StvP2 with 100 μM **8**, 4 mM NADPH, 200 μM acetyl CoA in 50 mM Tris-HCl buffer (pH 7.5) in the presence or absence of 100 μM Fdx and 5 μM FdR at 28°C for 3 h. All the reactions were terminated by identical volume of methanol, followed by centrifugation at 12,000 rpm for 5 min. Then the resulting supernatants were analyzed by LC-ESI-HRMS.

Time dependent conversion experiments of 1 or 2 by StvA2, and 2 by StvP2

A 100 μL reaction system containing 100 μM of **1** or **2** with 2.5 μM StvA2, 200 μM acetyl CoA in 50 mM Tris-HCl buffer (pH 7.5) was incubated at 28°C. Another 100 μL reaction system containing 100 μM of **2** was incubated with 2.5 μM StvP2, 5 μM FdR, 100 μM Fdx and 3 mM NADPH in 50 mM Tris-HCl buffer (pH 7.5) at 28°C. The time points for reactions were set at 0, 1, 2, 4, 6, 10, 20, 40, 60, 120 min, respectively. When each reaction reached to the reaction time point, the reaction mixture was terminated with identical volume (100 μL) of methanol. After centrifuged at 12,000 rpm for 10 min and filtrated with nylon filter membrane, the mixture was analyzed by HPLC at *λ* 245 nm.

Supplementary Tables

Supplementary Table 1. ^{1}H and ^{13}C NMR data of compounds 1 and 2.

	**1**[a]			**2**[b]	
	δ_C	δ_H		δ_C	δ_H
1	153.3	—	1	147.7	—
2	134.5	—	2	128.8	—
3	121.5	—	3	119.1	—
4	136.2	—	4	138.1	—
5	101.9	—	5	117.8	—
6	159.1	—	6	157.3	—
7	107.4	—	7	114.7	—
8	188.5	—	8	155.4	—
9	113.2	—	9	112.3	—
10	125.2	—	10	126.4	—
11	168.5	—	11	201.4	—
12	130.2	—	12	139.5	—
13	89.3	5.81 (d, *J* = 4.4 Hz, 1H)<br>5.15 (d, *J* = 4.4 Hz, 1H)	13	62.6	3.70 (s, 3H)
14	7.5	1.95 (s, 3H)	14	9.3	2.23 (s, 3H)
15	169.2	—	15	174.5	—
16	126.4	—	16	133.1	—
17	135.3	7.74 (d, *J* = 12.1 Hz, 1H)	17	131.1	7.22 (d, *J* = 11.6 Hz, 1H)
18	124.1	6.47 (t, *J* = 11.4 Hz, 1H)	18	124.1	6.43 (t, *J* = 11.3 Hz, 1H)
19	144.0	5.76 (t, *J* = 9.8 Hz, 1H)	19	144.4	5.62 (t, *J* = 10.3 Hz, 1H)
20	38.3	3.04 (p, *J* = 6.7 Hz, 1H)	20	39.6	3.14 (tq, *J* = 10.3, 6.6 Hz, 1H)
21	82.6	3.54 (m, 1H)	21	83.2	3.40 (d, *J* = 10.3 Hz, 1H)
22	41.8	2.18 (p, *J* = 6.9 Hz, 1H)	22	35.4	1.87 (q, *J* = 7.1 Hz, 1H)
23	77.3	4.15 (m, 1H)	23	76.0	4.13 (d, *J* = 11.1 Hz, 1H)
24	47.1	2.86 (t, *J* = 2.6 Hz, 1H)	24	54.6	2.97 (dd, *J* = 11.1, 2.6 Hz, 1H)
25	70.4	4.13 (m, 1H)	25	71.3	3.75 (dd, *J* = 10.9, 2.5 Hz, 1H)
26	38.9	1.96 (p, *J* = 6.9 Hz, 1H)	26	38.7	1.69 (dq, *J* = 10.9, 7.1 Hz, 1H)
27	73.7	3.44 (d, *J*= 4.5 Hz, 1H)	27	77.3	3.73 (d, *J* = 1.7 Hz, 1H)
28	77.2	—	28	76.2	—
29	153.7	5.63 (q, *J* = 1.3 Hz, 1H)	29	150.4	6.12 (d, *J* = 0.5 Hz, 1H)
30	12.7	2.20 (s, 3H)	30	12.7	2.04 (s, 3H)
31	22.1	1.20 (d, *J* = 6.7 Hz, 3H)	31	18.1	0.90 (d, *J* = 6.6 Hz, 3H)
32	15.7	0.94 (d, *J* = 6.4 Hz, 3H)	32	21.9	1.28 (d, *J* = 7.2 Hz, 3H)
33	173.2	—	33	174.4	—
34	52.0	3.69 (s, 3H)	34	52.1	3.69 (s, 3H)
35	10.4	0.94 (d, *J* = 6.4 Hz, 3H)	35	10.1	0.04 (d, *J* = 6.9 Hz, 3H)
36	22.1	1.12 (s, 3H)	36	28.4	1.26 (s, 3H)
37	13.1	2.27 (d, *J* = 1.4 Hz, 3H)	37	12.0	2.27 (d, *J* = 1.2 Hz, 3H)
38	168.9	—	38	170.5	—
39	21.2	2.27 (s, 3H)	39	20.7	2.20 (s, 3H)
40	14.0	2.00 (s, 3H)	40	12.5	2.00 (s, 3H)

Note: [a], ^{1}H NMR data of compound **1** were acquired on 400 MHz using $CHCl_3$-d_1 as solvent, and ^{13}C NMR data on 100 MHz; [b], ^{1}H NMR data of compound **2** was acquired on 600 MHz using CH_3OH-d_4 as solvent, and ^{13}C NMR data on 150 MHz.

Supplementary Table 2. ^{1}H (600 MHz) and ^{13}C (150 MHz) NMR data of compounds 3 and 4.

	**3**[a]			**4**[b]	
	***δ*C**	***δ*H**		***δ*C**	***δ*H**
1	153.9	—	1	147.5	—
2	134.5	—	2	128.1	—
3	121.0	—	3	119.2	—
4	136.1	—	4	138.0	—
5	101.8	—	5	117.9	—
6	159.1	—	6	157.3	—
7	107.5	—	7	114.8	—
8	188.4	—	8	155.5	—
9	113.3	—	9	112.3	—
10	125.6	—	10	126.5	—
11	168.7	—	11	201.4	—
12	130.2	—	12	139.5	—
13	89.3	5.82 (d, *J* = 4.4 Hz, 1H)<br>5.16 (d, *J* = 4.4 Hz, 1H)	13	62.5	3.70 (s, 3H)
14	7.5	1.95 (s, 3H)	14	9.3	2.22 (s, 3H)
15	170.1	—	15	175.0	—
16	126.5	—	16	132.9	—
17	135.5	7.72 (d, *J* = 12.2 Hz, 1H)	17	131.7	7.20 (d, *J* = 11.4 Hz, 1H)
18	124.3	6.51 (t, *J* = 11.5 Hz, 1H)	18	123.9	6.42 (t, *J* = 11.1 Hz, 1H)
19	144.8	5.77 (t, *J* = 9.6 Hz, 1H)	19	144.7	5.70 (t, *J* = 10.1 Hz, 1H)
20	39.4	2.96 (p, *J* = 7.1 Hz, 1H)	20	39.6	3.12 (m, 1H)
21	83.0	3.46 (d, *J* = 7.8 Hz, 1H)	21	83.3	3.41 (d, *J* = 9.7 Hz, 1H)
22	42.6	2.24 (m, 1H)	22	34.3	2.12 (m, 1H)
23	81.3	3.61 (d, *J* = 9.2 Hz, 1H)	23	80.3	3.56 (d, *J* = 9.9 Hz, 1H)
24	32.7	1.97 (m, 1H)	24	40.0	1.85 (m, 1H)
25	71.3	4.03 (d, *J* = 9.2 Hz, 1H)	25	71.8	3.68 (d, *J* = 14.1 Hz, 3H)
26	37.2	1.73 (p, *J* = 7.6 Hz, 1H)	26	37.2	1.84 (m, 1H)
27	73.6	3.42 (d, *J* = 4.5 Hz, 1H)	27	77.6	3.63 (m, 1H)
28	77.3	—	28	76.6	—
29	153.5	5.61 (s, 1H)	29	151.0	6.13 (s, 1H)
30	12.9	2.23 (s, 3H)	30	12.7	2.03 (m, 1H)
31	22.7	1.24 (d, *J* = 6.8 Hz, 3H)	31	18.4	1.01 (d, *J* = 6.5 Hz, 3H)
32	14.8	0.90 (d, *J* = 6.1 Hz, 3H)	32	22.0	1.25 (d, *J* = 6.5 Hz, 3H)
33	10.7	0.99 (d, *J* = 7.0 Hz, 3H)	33	9.5	0.80 (d, *J* = 6.3 Hz, 3H)
34	—	—	34	—	—
35	9.9	0.90 (d, *J* = 6.4 Hz, 3H)	35	10.3	0.03 (d, *J* = 6.0 Hz, 3H)
36	22.0	1.14 (s, 3H)	36	27.9	1.25 (s, 3H)
37	13.1	2.29 (s, 3H)	37	11.8	2.27 (d, *J* = 0.7 Hz, 3H)
38	168.8	—	38	170.5	—
39	21.2	2.28 (s, 3H)	39	20.7	2.18 (s, 3H)
40	13.8	2.00 (s, 3H)	40	12.6	1.99 (s, 3H)

Note: [a], the sample of compound **3** was dissolved in $CHCl_3$-d_1 solvent; [b], the sample of compound **4** was dissolved in CH_3OH-d_4 solvent.

Supplementary Table 3. ^{1}H (600 MHz) and ^{13}C (150 MHz) NMR Data of compounds 5 and 6 ($CHCl_3$-d_1).

	5			6	
	δ_C	δ_H		δ_C	δ_H
1	152.7	—	1	145.4	—
2	133.8	—	2	124.8	—
3	121.1	—	3	118.3	—
4	136.5	—	4	136.1	—
5	101.6	—	5	115.4	—
6	159.1	—	6	157.5	—
7	107.0	—	7	115.5	—
8	188.5	—	8	155.5	—
9	113.2	—	9	111.6	—
10	125.3	—	10	126.8	—
11	168.5	—	11	196.3	—
12	127.2	—	12	141.0	—
13	89.4	5.82 (d, *J* = 4.4 Hz, 1H)<br>5.19 (d, *J* = 4.4 Hz, 1H)	13	63.3	3.83 (s, 3H)
14	7.5	1.97 (s, 3H)	14	9.1	2.27 (s, 3H)
15	169.6	—	15	168.8	—
16	127.2	—	16	130.6	—
17	135.0	7.60 (d, *J* = 12.2 Hz, 1H)	17	130.7	7.43 (d, *J* = 10.8 Hz, 1H)
18	123.7	6.46 (t, *J* = 11.8 Hz, 1H)	18	125.4	6.49 (t, *J* = 10.8 Hz, 1H)
19	144.2	5.76 (t, *J* = 9.6 Hz, 1H)	19	143.1	5.66 (t, *J* = 10.3 Hz, 1H)
20	38.8	2.94 (p, *J* = 6.9 Hz, 1H)	20	36.7	3.10 (tt, *J* = 16.0, 7.8 Hz, 1H)
21	83.5	3.50 (d, *J* = 8.0 Hz, 1H)	21	77.7	3.23 (d, *J* = 7.8 Hz, 1H)
22	42.0	2.25 (p, *J* = 6.9 Hz, 1H)	22	40.8	2.25 (m, 1H)
23	77.0	4.16 (m, 1H)	23	69.1	4.49 (dd, *J* = 9.4, 1.3 Hz, 1H)
24	47.0	2.86 (t, *J* = 2.6 Hz, 1H)	24	51.5	2.56 (dd, *J* = 9.4, 3.0 Hz, 1H)
25	69.9	4.13 (m, 1H)	25	77.0	4.24 (s, 1H)
26	38.4	1.98 (p, *J* = 6.9 Hz, 1H)	26	35.4	1.88 (q, *J* = 6.9 Hz, 1H)
27	73.2	3.36 (dd, *J* = 8.5, 4.5 Hz, 1H)	27	75.3	2.70 (m, 1H)
28	37.5	2.56 (m, 1H)	28	38.1	2.72 (m, 1H)
29	153.4	5.41 (dd, *J* = 9.7, 1.0 Hz, 1H)	29	146.4	5.05 (d, *J* = 8.7 Hz, 1H)
30	12.8	2.21 (s, 3H)	30	12.7	2.11 (s, 3H)
31	22.3	1.24 (d, *J* = 6.9 Hz, 3H)	31	17.2	1.06 (d, *J* = 6.5 Hz, 3H)
32	15.4	0.96 (d, *J* = 6.6 Hz, 3H)	32	12.8	1.04 (d, *J* = 7.3 Hz, 3H)
33	172.5	—	33	174.8	—
34	51.9	3.70 (s, 3H)	34	51.3	3.02 (s, 3H)
35	9.1	0.77 (d, *J* = 6.9 Hz, 3H)	35	13.2	1.16 (d, *J* = 7.2 Hz, 3H)
36	15.7	0.68 (d, *J* = 7.0 Hz, 3H)	36	14.9	0.58 (d, *J* = 5.6 Hz, 3H)
37	12.7	2.02 (d, *J* = 1.0 Hz, 3H)	37	12.2	2.04 (s, 3H)
38	169.0	—	38	167.9	—
39	21.3	2.27 (s, 3H)	39	20.4	2.21 (s, 3H)
40	14.4	1.98 (s, 3H)	40	12.6	2.23 (s, 3H)

Supplementary Table 4. Kinetic parameters of StvP2 catalyzing substrates 2, 4 and 6 to form MDB.

	V_{max} (min^{-1})	V_i (min^{-1})	K_s (μM)	K_i (μM)	n^H	Reduced Chi-Sqr	R^2 (COD)
**2**	19.82 ± 6.21	4.04 ± 0.71	109.66 ± 46.19	367.30 ± 128.57	1.59 ± 0.36	0.28725	0.99331
**4**	14.45 ± 3.55	2.69 ± 0.59	95.56 ± 30.84	399.35 ± 122.10	1.71 ± 0.37	0.18687	0.99324
**6**	10.98 ± 4.11	0.65 ± 0.66	67.40 ± 28.39	288.50 ± 122.60	2.10 ± 0.83	0.38681	0.98074

The substrate inhibition kinetics are fitted with the modified Hill equation: $V = \left(V_{max} + V_i\left(\frac{[S]^2}{K_i^2}\right)\right) \Big/ \left(1 + \frac{K_s^{n^H}}{[S]^{n^H}} + \frac{[S]^2}{K_i^2}\right)$. V_{max} and V_i are the reaction velocity in the absence or presence of inhibition, corresponding to the catalytic constants k_{cat} and $k_{cat(i)}$, respectively, and n^H is the Hill coefficient. K_s and K_i are comparable to the $[S]_{1/2}$ values for the ascending and descending arms of the curve, respectively. Source data are provided as a Source Data file.

Supplementary Table 5. Data collection and refinement statistics.

	Substrate-free StvP2	Substrate-bound StvP2
**Data collection**		
Space group	*C* 2 2 2_1	*P* 1 2_1 1
Cell dimensions		
a, *b*, *c* (Å)	60.1 145.7 90.4	61.6 87.2 78.7
α, *β*, *γ* (°)	90 90 90	90 112.3 90
Wavelength (Å)	0.9789	0.9789
Resolution range (Å)	19.84 - 1.35 (1.398 - 1.35)	19.78 - 2.3 (2.382 - 2.3)
R_{merge} (%)	7.531 (>100)	11.97 (60.53)
$CC_{1/2}$ (%)	99.9 (78.8)	99.6 (84.1)
$I/σ(I)$	18.10 (2.11)	11.60 (2.57)
Completeness (%)	99.69 (97.48)	99.24 (99.91)
Redundancy	12.8 (10.1)	6.5 (6.7)
**Refinement**		
Resolution (Å)	19.84 - 1.35	19.78 - 2.3
No. reflections	86927 (8420)	34175 (3424)
R_{work} / R_{free}	0.1392 / 0.1703	0.2216 /0.2691
No. atoms		
Protein	2914	5765
Ligands	43	196
Water	421	301
B-factor		
Average *B*-factor	23.27	41.42
Protein	21.94	41.79
Ligands	14.34	32.23
Solvent	33.37	40.26
R.m.s. deviations		
Bond length (Å)	0.017	0.004
Bond angles (°)	1.44	0.92
Ramachandran statistics		
Favored (%)	99.19	96.49
Allowed (%)	0.81	3.51
Outliers (%)	0.00	0.00
PDB code	6M4Q	6M4P

Statistics for the highest-resolution shell are shown in parentheses.

Supplementary Table 6. ^{1}H (600 MHz) and ^{13}C (150 MHz) NMR Data of compounds 7 (DMSO-d_6) and 8 (CH_3OH-d_4).

	7			8	
	δ_C	δ_H		δ_C	δ_H
1	184.3	—	1	186.5	—
2	140.0	—	2	140.7	—
3	135.3	—	3	138.7	—
4	184.1	—	4	185.3	—
5	124.3	—	5	129.9	—
6	159.4	—	6	163.0	—
7	117.0	—	7	128.2	—
8	160.9	—	8	163.2	—
9	106.6	—	9	111.4	—
10	129.1	—	10	130.9	—
11	196.6	—	11	199.5	—
12	136.1	—	12	139.2	—
			13	63.1	3.79 (s, 3H)
14	8.8	2.12 (s, 3H)	14	9.5	2.26 (s, 3H)
15	169.6	—	15	172.3	—
16	131.0	—	16	132.7	—
17	131.6	7.10 (d, J = 11.2 Hz, 1H)	17	133.2	7.23 (d, J = 11.4 Hz, 1H)
18	122.5	6.30 (t, J = 10.9 Hz, 1H)	18	124.2	6.42 (t, J = 11.2 Hz, 1H)
19	144.4	5.61 (t, J = 10.1 Hz, 1H)	19	144.9	5.61 (t, J = 10.4 Hz, 1H)
20	38.1	2.96 (h, J = 6.7 Hz, 1H)	20	40.1	3.05 (tq, J = 10.5, 6.6 Hz, 1H)
21	80.9	3.29 (dd, J = 9.5, 6.5 Hz, 1H)	21	83.1	3.39 (d, J = 10.3 Hz, 1H)
22	33.6	1.77 (q, J = 7.0 Hz, 1H)	22	35.4	1.91 (m, 1H)
23	73.8	3.99 (t, J = 9.0 Hz, 1H)	23	75.9	4.13 (dd, J = 10.7, 1.8 Hz, 1H)
24	52.9	2.81 (dd, J = 10.7, 1.7 Hz, 1H)	24	54.3	2.96 (dd, J = 10.7, 2.5 Hz, 1H)
25	69.6	3.71 (ddd, J = 10.3, 7.9, 1.6 Hz, 1H)	25	71.3	3.83 (dd, J = 10.9, 2.5 Hz, 1H)
26	36.8	1.69 (dd, J = 10.3, 7.2 Hz, 1H)	26	38.7	1.76 (m, 1H)
27	75.3	3.68 (d, J = 6.3 Hz, 1H)	27	77.3	3.81 (d, J = 1.7 Hz, 1H)
28	74.2	—	28	75.9	—
29	144.1	6.31 (s, 1H)	29	146.3	6.36 (s, 1H)
30	12.4	1.89 (s, 3H)	30	12.5	2.00 (d, J = 1.0 Hz, 3H)
31	17.8	0.72 (d, J = 6.6 Hz, 3H)	31	18.0	0.85 (d, J = 6.6 Hz, 3H)
32	21.3	1.17 (d, J = 6.9 Hz, 3H)	32	22.0	1.27 (d, J = 7.2 Hz, 3H)
33	172.3	—	33	174.2	—
34	51.0	3.52 (s, 3H)	34	52.1	3.66 (s, 3H)
35	10.4	0.26 (d, J = 6.8 Hz, 3H)	35	11.9	0.39 (d, J = 6.9 Hz, 3H)
36	28.8	1.17 (s, 3H)	36	28.3	1.29 (s, 3H)
37	12.2	2.11 (d, J = 1.0 Hz, 3H)	37	12.4	2.23 (d, J = 1.2 Hz, 3H)
40	13.0	1.72 (s, 3H)	40	13.8	1.89 (s, 3H)

Supplementary Table 7. Bacterial strains used in this study.

Strain	Characteristic	Reference
Escherichia coli		
DH10B	Host for general DNA manipulation	Invitrogen
ET12567/pUZ8002	Donor strain for conjugation between *E. coli* and *Streptomyces*	4
BL21(DE3)	Host for recombinant protein expression	NEB
C43(DE3)	Host derived from BL21(DE3) for recombinant protein expression	NEB
Streptomyces spectabilis		
CCTCC M2017417	Streptovaricins producing wild-type strain	5
ΔstvP1	*stvP1* in-frame deletion mutant	5
ΔstvP2	*stvP2* in-frame deletion mutant	5
ΔstvP1P4	*stvP1* and *stvP4* double in-frame deletion mutant	This study
ΔstvP1P5	*stvP1* and *stvP5* double in-frame deletion mutant	This study
ΔstvP1P4P2	*stvP1*, *stvP4* and *stvP2* triple in-frame deletion mutant	This study
ΔstvP1P5P2	*stvP1*, *stvP5* and *stvP2* triple in-frame deletion mutant	This study
ΔstvM1	*stvM1* in-frame deletion mutant	This study
ΔstvA1	*stvA1* in-frame deletion mutant	This study
ΔstvA2	*StvA2* in-frame deletion mutant	This study

Supplementary Table 8. Plasmids used in this study.

Plasmid	Description	Reference
pYH7	*Streptomyces-E. coil* shuttle vector	6
pET28a(+)	Vector for recombinant protein expression	Invitrogen
pET-His	Vector derivated from pET28a(+) for recombinant protein expression	This study
pET-His-StvP2	Plasmid for recombinant protein StvP2 expression used for biochemical assay and cultivation of co-crystal	This study
pET28a(+)-StvM1	Plasmid for StvM1 expression	This study
pET28a(+)-StvA2	Plasmid for StvA2 expression	This study
pWHU2801	Plasmid for *stvP4* in-frame deletion	5
pWHU2802	Plasmid for *stvP5* in-frame deletion	5
pWHU2907	Plasmid for *stvP2* in-frame deletion based on ΔstvP1P4 or ΔstvP1P5 double in-frame deletion mutant	This study
pWHU2820	Plasmid for *stvM1* in-frame deletion	This study
pWHU2905	Plasmid for *stvA1* in-frame deletion	This study
pWHU2906	Plasmid for *stvA2* in-frame deletion	This study

Supplementary Table 9. Primers used in this study.

Primers	Oligonucleotide sequences (5' to 3')
Primers for homologous arm construction	
stvM1-L-For	GAGCATATGACGCTCAACGGCCTCC (*Nde*I)
stvM1-L-Rev	CTGCTGCAGATCTGGCTCCTCGAGG (*Pst*I)
stvM1-R-For	CTCCTGCAGCACGCCCTCCACCAG (*Pst*I)
stvM1-R-Rev	GCCAAGCTTAAGAACGCCATCGACT (*Hin*dIII)
stvA1-L-For	CTTCATATGATGCTGGAGAAGATC (*Nde*I)
stvA1-L-Rev	GAGGAATTCCCGTCGACGTGGTGAC (*Eco*R1)
stvA1-R-For	CGCGAATTCACTCATCGTCGCCGCT (*Eco*R1)
stvA1-R-Rev	TGCAAGCTTGCCCAGTACTCCACCC (*Hin*dIII)
stvA2-L-For	TCCCATATGCGAGGTAACTCTCCAC (*Nde*I)
stvA2-L-Rev	GTTCTGCAGCGTGTAGCCCACGTA (*Pst*I)
stvA2-R-For	ACGCTGCAGAACCGCCTGACGTCGC (*Pst*I)
stvA2-R-Rev	CGCAAGCTTGAGCGGATGCCCTGGG (*Hin*dIII)
stvP4-L-For	GCGCATATGGAGCTGAATGGGACC (*Nde*I)
stvP4-L-Rev	CGCCTGCAGTGACCGGCCACCGACC (*Pst*I)
stvP4-R-For	GTTCTGCAGGGTGGCTGTCATGGG (*Pst*I)
stvP4-R-Rev	GGGAAGCTTCGACGGACACGCTCAC (*Hin*dIII)
stvP5-L-For	AGACATATGACTTCGTCGATGACCG (*Nde*I)
stvP5-L-Rev	CTTCTGCAGCGCCTCGTCTGAGCAC (*Pst*I)
stvP5-R-For	GTTCTGCAGGGTGGCTGTCATAGGT (*Pst*I)
stvP5-R-Rev	GTCAAGCTTGTCGTCGAGCAGCTCC (*Hin*dIII)
stvP2'-L-For	AAGGCGAATACTTCACGGATGGTTCGGCACCAG
stvP2'-L-Rev	TGCCGACATGGTCACCTGGTAGGTGACGGG
stvP2'-R-For	ACCAGGTGACCATGTCGGCACCCTCGTGCG
stvP2'-R-Rev	ACCTGCAGGCATGCAACCCTGACCTTCGTCGGCGA
Primers for mutants verification	
CK-stvM1-For	TCCGGTAGCGCGTGTGGTCC
CK-stvM1-Rev	GAGCGCCGGGGAAAATTACC
CK-stvA1-For	TGCTGGTGACCGTGGCGAGG
CK-stvA1-Rev	AACGGCGCGGCCGCGCACCT
CK-stvA2-For	CATGCTGCTTGAATCTCTGT
CK-stvA2-Rev	CTTCGCCGACCCGGACACGC
CK-stvP1-For	GTGCTGGTATGCGTTGTCGT
CK-stvP1-Rev	GGAGCGCCAAGATCGCCGAA
CK-stvP4-For	GATGGCGTCTACCGGCCGTA
CK-stvP4-Rev	AAGAGCGCGGAATCCTTATC
CK-stvP5- For	TCGACGTACCGGAAGTGGCT
CK-stvP5- Rev	TCCAGGACGGTCACGCGCAG
CK-stvP2- For	AACTGGGGCGTCGTGCCGAC
CK-stvP2- Rev	AAGCAGCGCCGCGCGTACGC

Primers for protein expression vector construction

StvM1-For	TGCCGCGCGGCAGCCATATGACCGACGACAACGCATA
StvM1-Rev	GACGGAGCTCGAATTCTCAGTCGCGCGCCGCCTCG
StvA2-For	CGCGCGGCAGCCATATGATGACGACAGC
StvA2-Rev	ACGGAGCTCGAATTCTCAGGCCTGCGACGTCAGGC
StvP2-pet-his-1	CAAGGAATGGTGCATGCA
StvP2-pet-his-2	TGATGATGATGGTGATGACC
StvP2-pet-his-3	TCACCATCATCATCATATGACCGAGACGCCCG
StvP2-pet-his-4	TTGTCACGGAGCTCGAATTCCTACCAGGTGACCGGCAG

Mutagenesis primers for StvP2

H92A-For	ATGCTCGCGATCGACCCGCCGGACCAC
H92A-Rev	GTCGATCGCGAGCATGTCGCCGGGCAG
H92D-For	ATGCTCGATATCGACCCGCCGGACC
H92D-Rev	GTCGATATCGAGCATGTCGCCGGGCAG
H92K-For	ATGCTCAAAATCGACCCGCCGGACCAC
H92K-Rev	GTCGATTTTGAGCATGTCGCCGGGCAG
D89A-For	CCGGCGCGATGCTCCACATCGACCCGCCG
D89A-Rev	GGAGCATCGCGCCGGGCAGCGGCGGG
D89E-For	CCGGCGAAATGCTCCACATCGACCCGCCG
D89E-Rev	GGAGCATTTCGCCGGGCAGCGGCGGG
D89K-For	CCGGCAAAATGCTCCACATCGACCCGCCG
D89K-Rev	GGAGCATTTTGCCGGGCAGCGGCGGG
R72A-For	GACAGGGCGCACTGGCCCCTGGCCGAC
R72A-Rev	CCAGTGCGCCCTGTCGTTGCGGTGGCT
R72E-For	GACAGGGAGCACTGGCCCCTGGCCGAC
R72E -Rev	CCAGTGCTCCCTGTCGTTGCGGTGGCT
R72K-For	GACAGGAAGCACTGGCCCCTGGCCGAC
R72K-Rev	CCAGTGCTTCCTGTCGTTGCGGTGGCT

Supplementary Figures

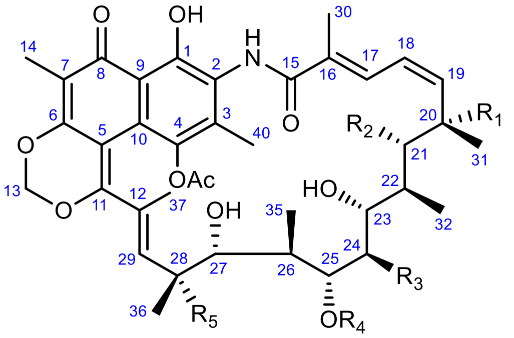

Streptovaricins	R_1	R_2	R_3	R_4	R_5
A	OH	OH	$COOCH_3$	CH_3CO	OH
B	H	OH	$COOCH_3$	CH_3CO	OH
C	H	OH	$COOCH_3$	H	OH
D	H	OH	$COOCH_3$	H	H
E	H	=O	$COOCH_3$	H	OH
F	H	R_3+R_2 = COO		H	OH
G	OH	OH	$COOCH_3$	H	OH
J	H	CH_3COO	$COOCH_3$	H	OH
K	OH	CH_3COO	$COOCH_3$	H	OH

Supplementary Fig. 1 MDB-contained streptovaricins reported.

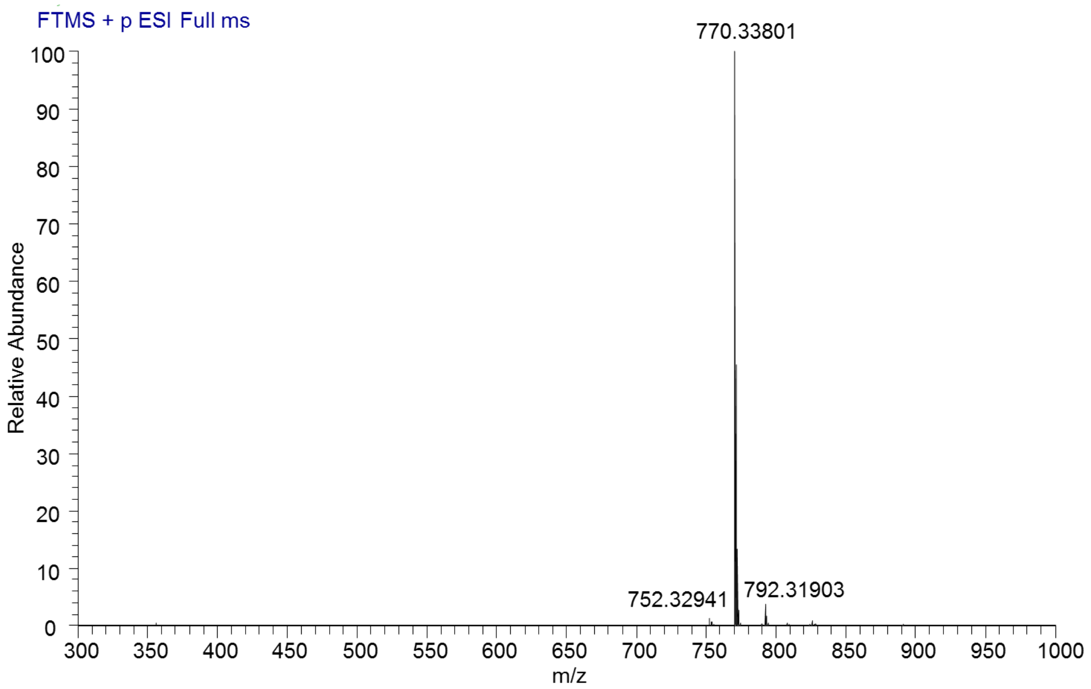

Supplementary Fig. 2 ESI-HRMS spectrum of streptovaricin C (1).

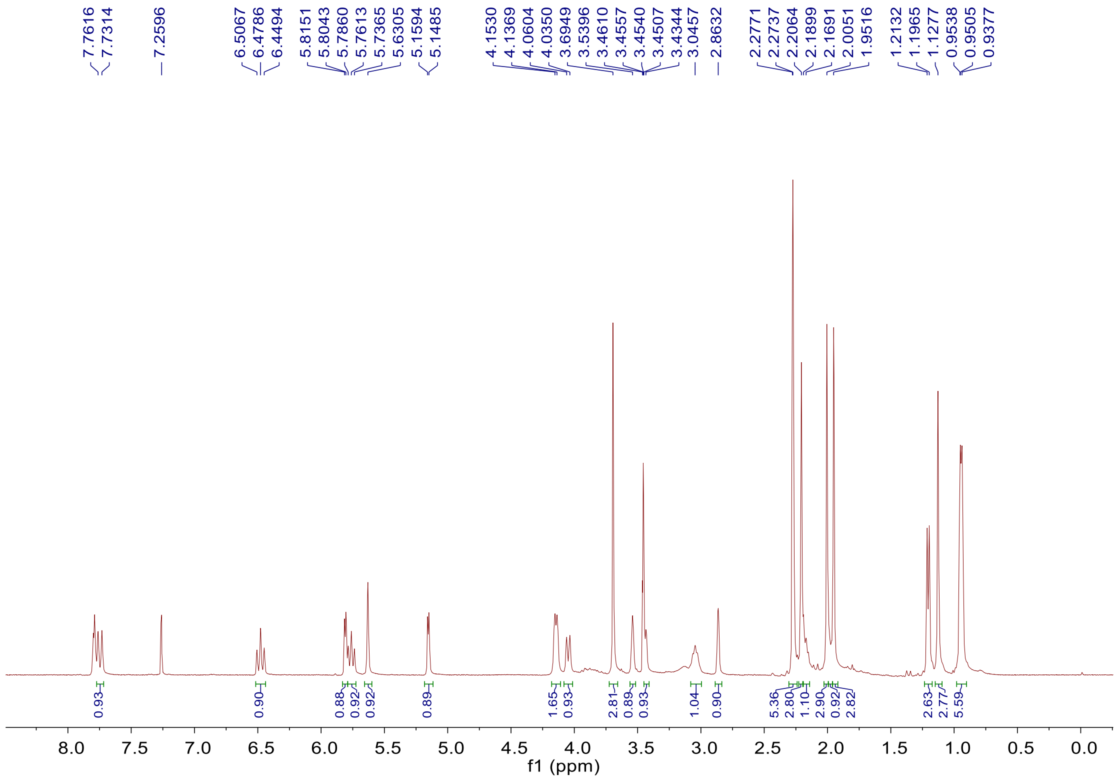

Supplementary Fig. 3 ^{1}H NMR spectrum (400 MHz, $CHCl_3$-d_1) of streptovaricin C (1).

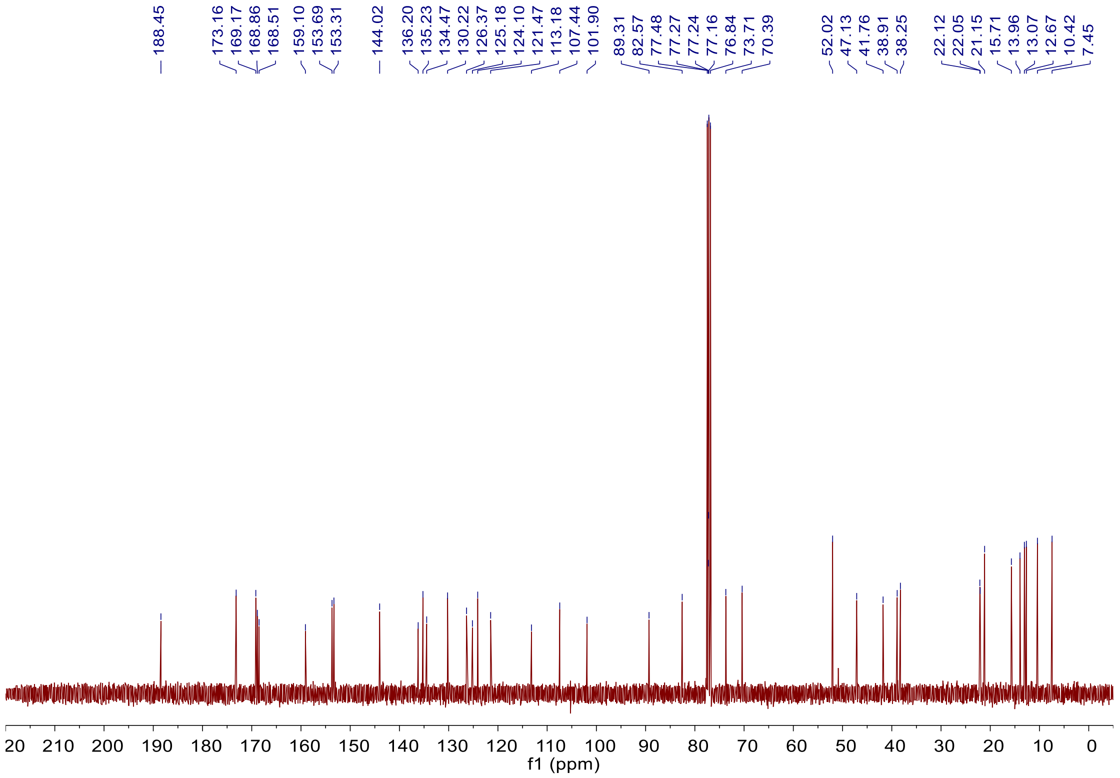

Supplementary Fig. 4 ^{13}C NMR spectrum (100 MHz, $CHCl_3$-d_1) of streptovaricin C (1).

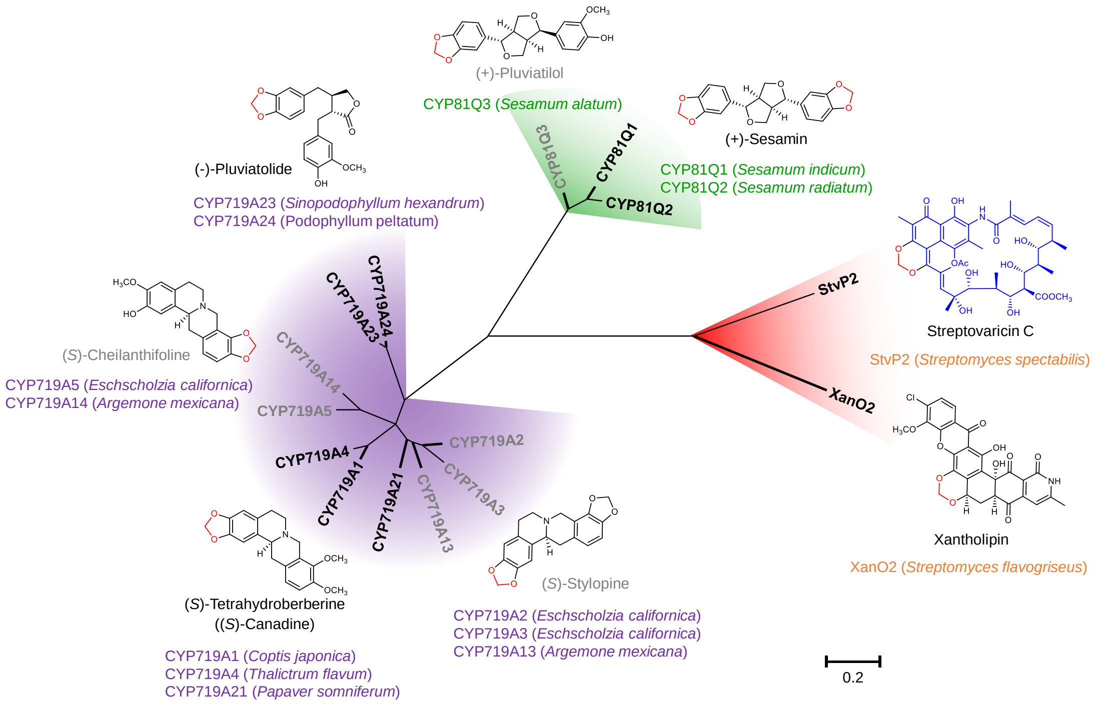

Supplementary Fig. 5 Phylogenetic tree of cytochrome P450 enzymes for MDB formation of natural products. Amino acid sequences used for the analysis were obtained from GenBank with accession numbers: CYP719A1 (No. AB026122, Canadine synthase, *Coptis japonica*); CYP719A2 (No. AB126257, Stylopine synthase, *Eschscholzia californica*); CYP719A3 (No. AB126256, Stylopine synthase, *Eschscholzia californica*); CYP719A4 (No. AY610513, Canadine synthase, *Thalictrum flavum*); CYP719A5 (No. AB434654, Cheilanthifoline synthase, *Eschscholzia californica*); CYP719A13 (No. EF451151, Stylopine synthase, *Argemone mexicana*); CYP719A14 (No. EF451152, Cheilanthifoline synthase, *Argemone mexicana*); CYP719A21 (No. JQ659003, Canadine synthase, *Papaver somniferum*); CYP719A23 (No. KC110997, Pluviatolide synthase, *Sinopodophyllum hexandrum*); CYP719A24 (No. KC110998, Pluviatolide synthase, *Podophyllum peltatum*); CYP81Q1 (No. KP771974, (+)-Piperitol/(+)-sesamin synthase, *Sesamum indicum*); CYP81Q2 (No. AB194715, (+)-Piperitol/(+)-sesamin synthase, *Sesamum radiatum*); CYP81Q3 (No. AB566040, (+)-Pluviatilol synthase, *Sesamum alatum*); XanO2 (No. GQ421798, P450 hydroxylase, *Streptomyces flavogriseus*); StvP2 (No. No. KY593296, P450 hydroxylase, *Streptomyces spectabilis*). The alignment was obtained using ClustalW in MEGA6 and the phylogenetic tree was constructed using MEGA6 based on the neighbor-joining method.

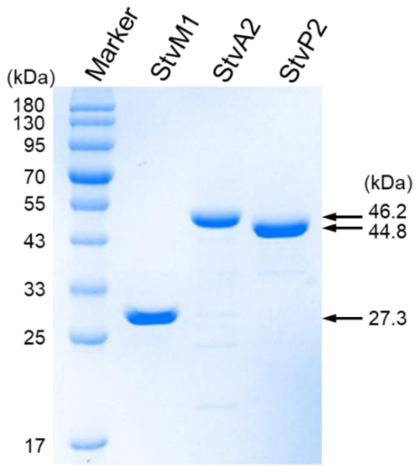

Supplementary Fig. 6 SDS-PAGE analysis of recombination proteins StvM1, StvA2 and StvP2. The results are representative of three independent experiments. Source data are provided as a Source Data file.

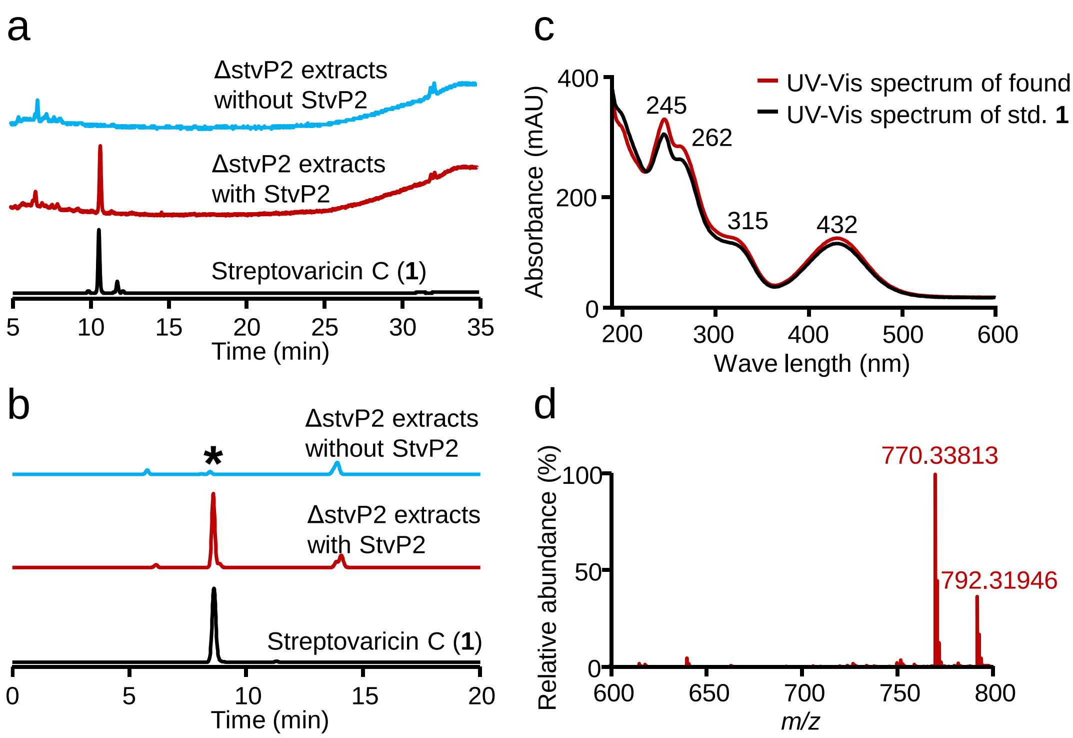

Supplementary Fig. 7 *In vitro* enzymatic conversion of the crude extract from ΔstvP2 catalyzed by StvP2. (**a**) HPLC-DAD comparative analysis of enzymatic conversion of crude extracts from ΔstvP2 with or without StvP2 in the presence of Fdx, FdR and NADPH. All traces were monitored at λ_{max}432 nm. (**b**) Confirmation of the results of (**a**) by LC-ESI-HRMS analysis. The asterisk indicated no component corresponding to **1** was detected. (**c**) UV/Vis spectrum of the newly produced peak corresponding to **1**. (**d**) ESI-HRMS spectrum of the newly produced peak corresponding to **1** (calcd. *m/z* 770.33823 $[M + H]^+$; 792.32018 $[M + Na]^+$). The pure compound **1** identified by NMR was used as standard. Experiments in **a-d** are representative of three independent experiments.

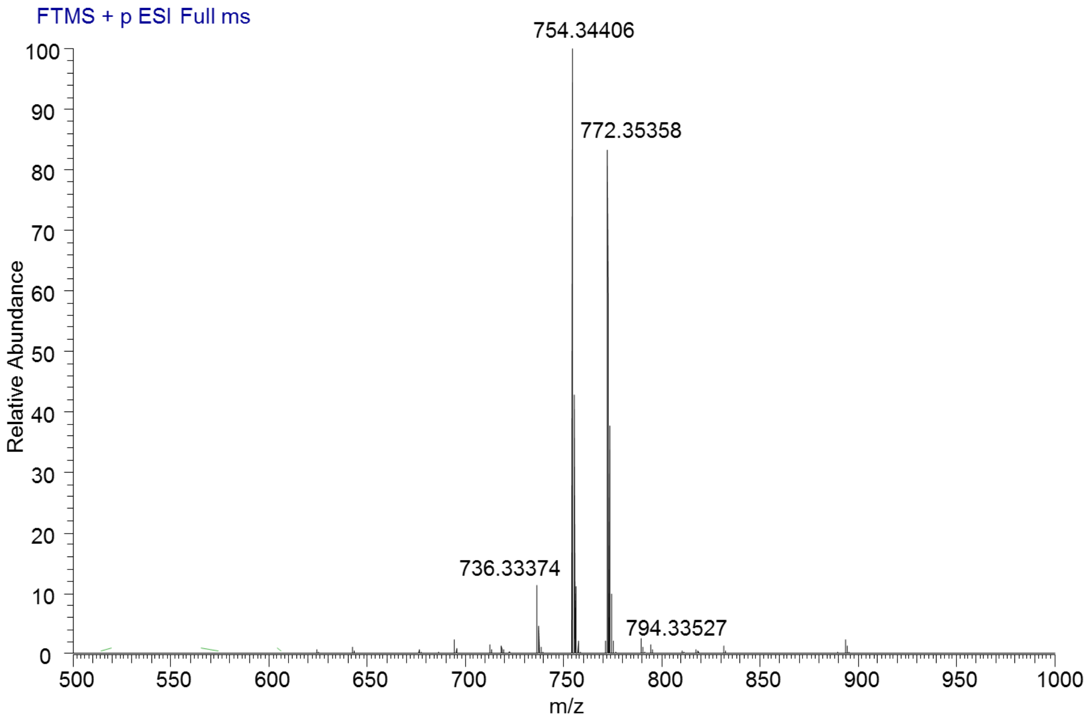

Supplementary Fig. 8 ESI-HRMS spectrum of 6-methoxy-streptovaricin C (2).

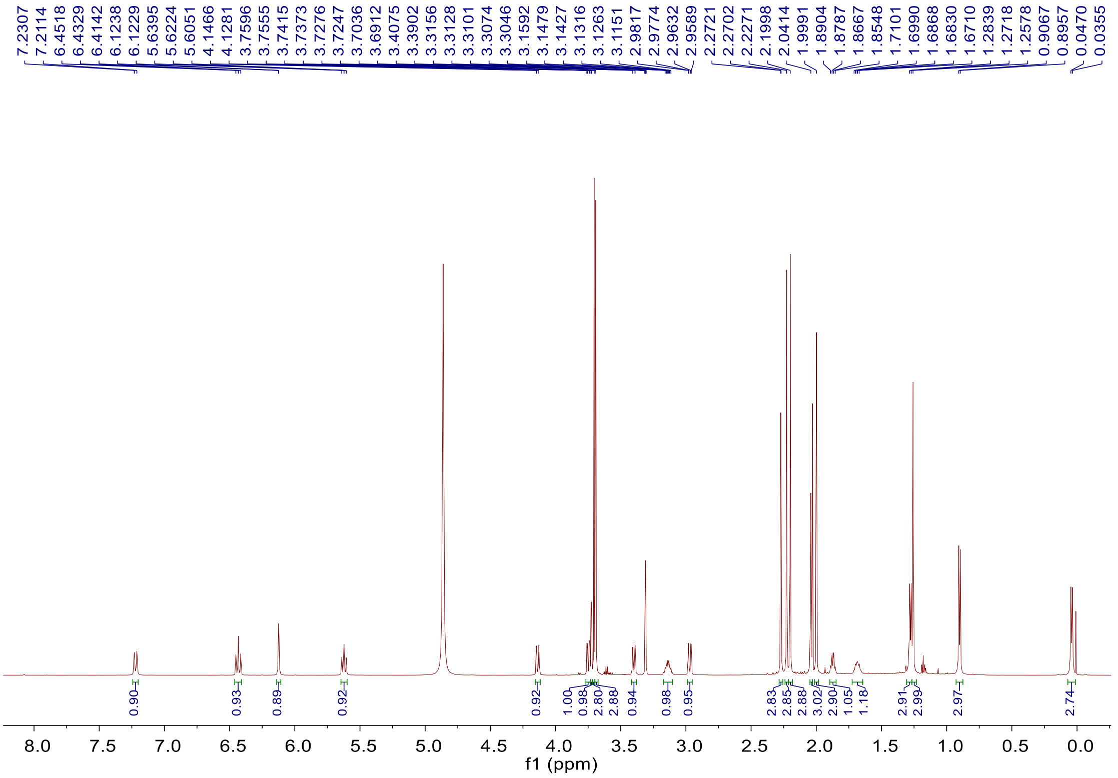

Supplementary Fig. 9 ^{1}H NMR spectrum (600 MHz, CH_3OH-d_4) of 6-methoxy-streptovaricin C (2).

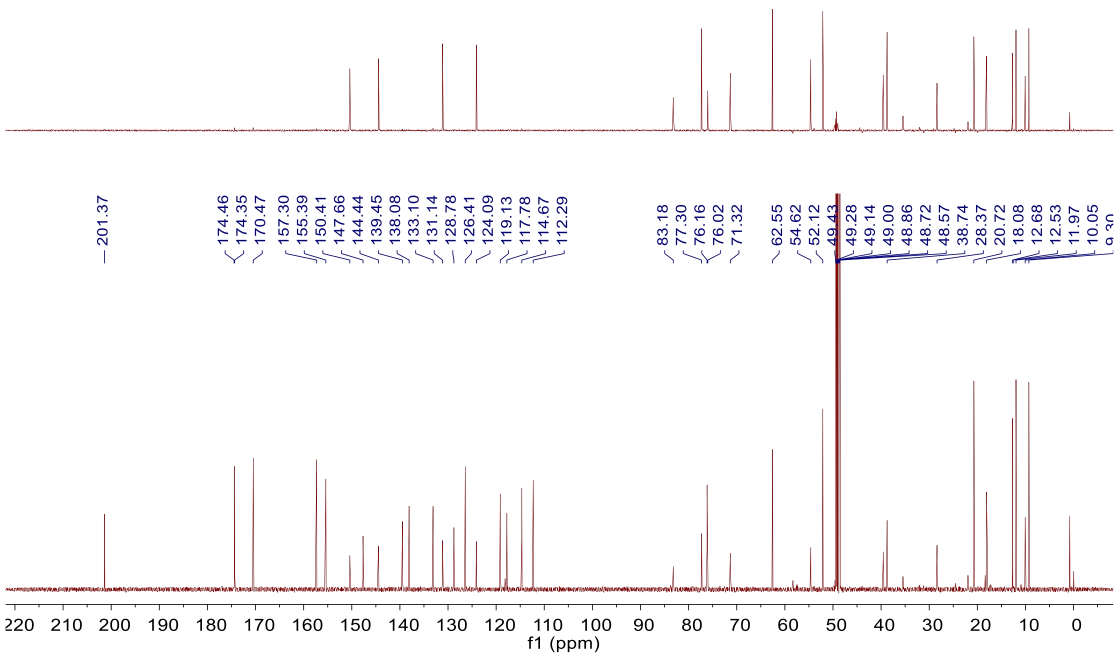

Supplementary Fig. 10 ^{13}C NMR and DEPT135 spectrum (150 MHz, CH_3OH-d_4) of 6-methoxy-streptovaricin C (2).

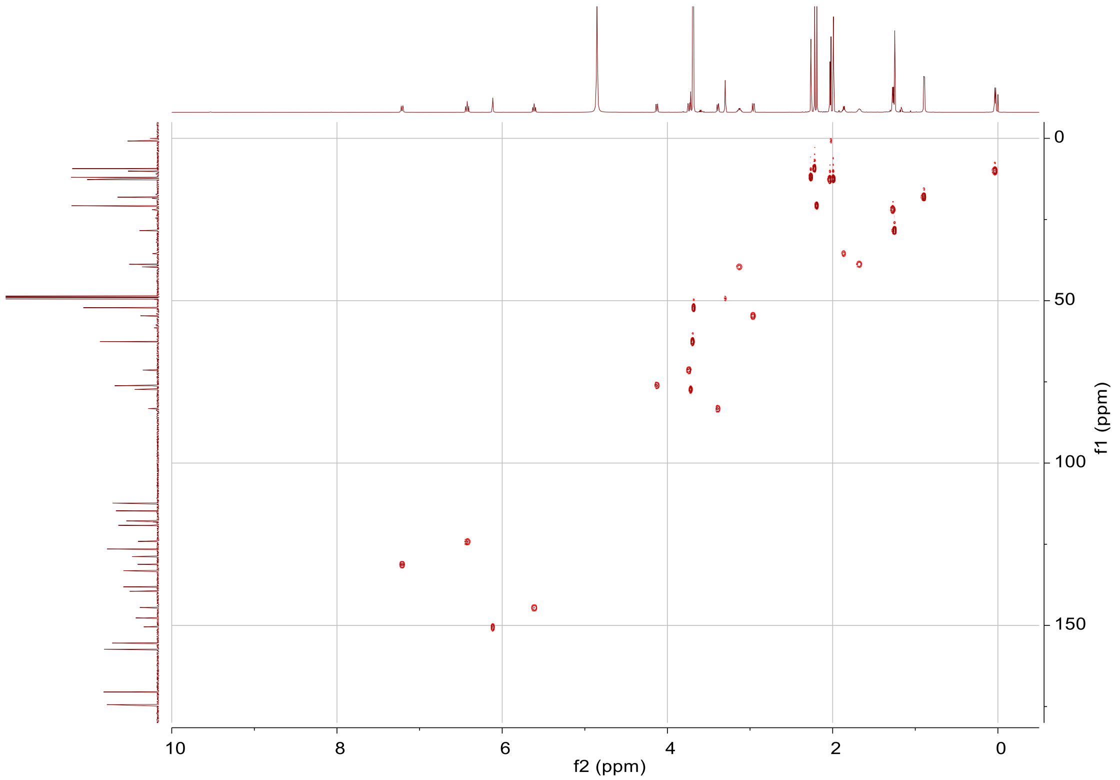

Supplementary Fig. 11 HSQC spectrum (600 MHz, CH_3OH-d_4)of 6-methoxy-streptovaricin C (2)

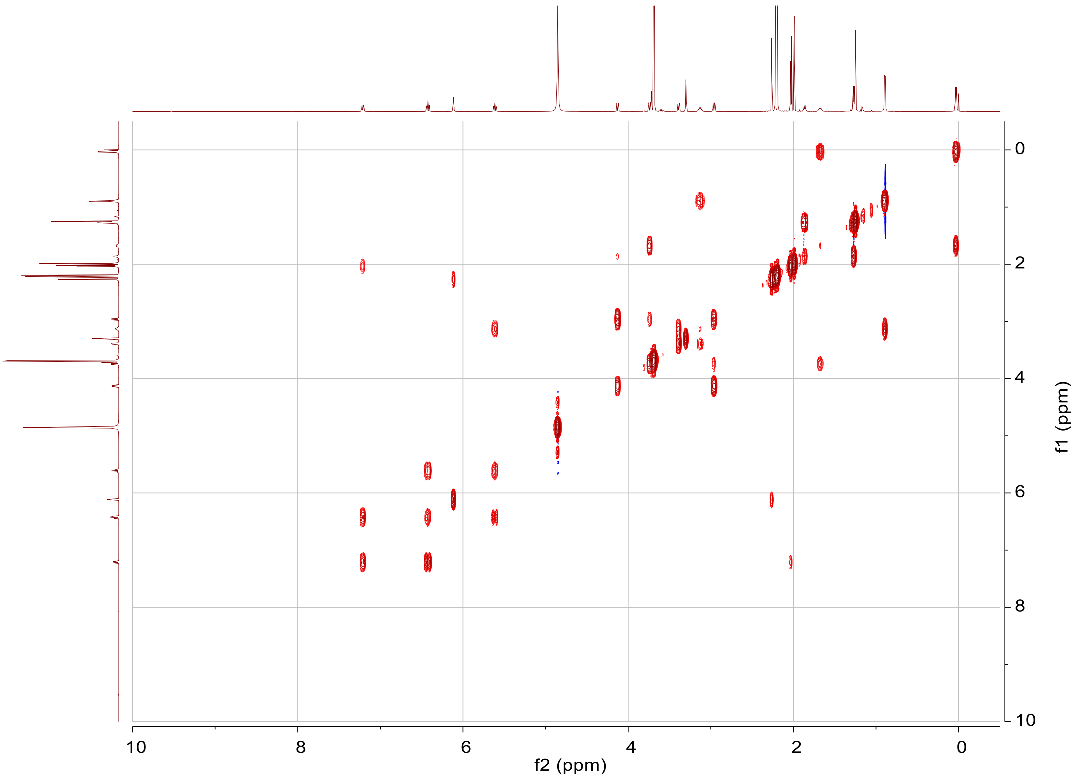

Supplementary Fig. 12 ^{1}H-^{1}H COSY spectrum (600 MHz, CH_3OH-*d*$_4$) of 6-methoxy-streptovaricin C (2).

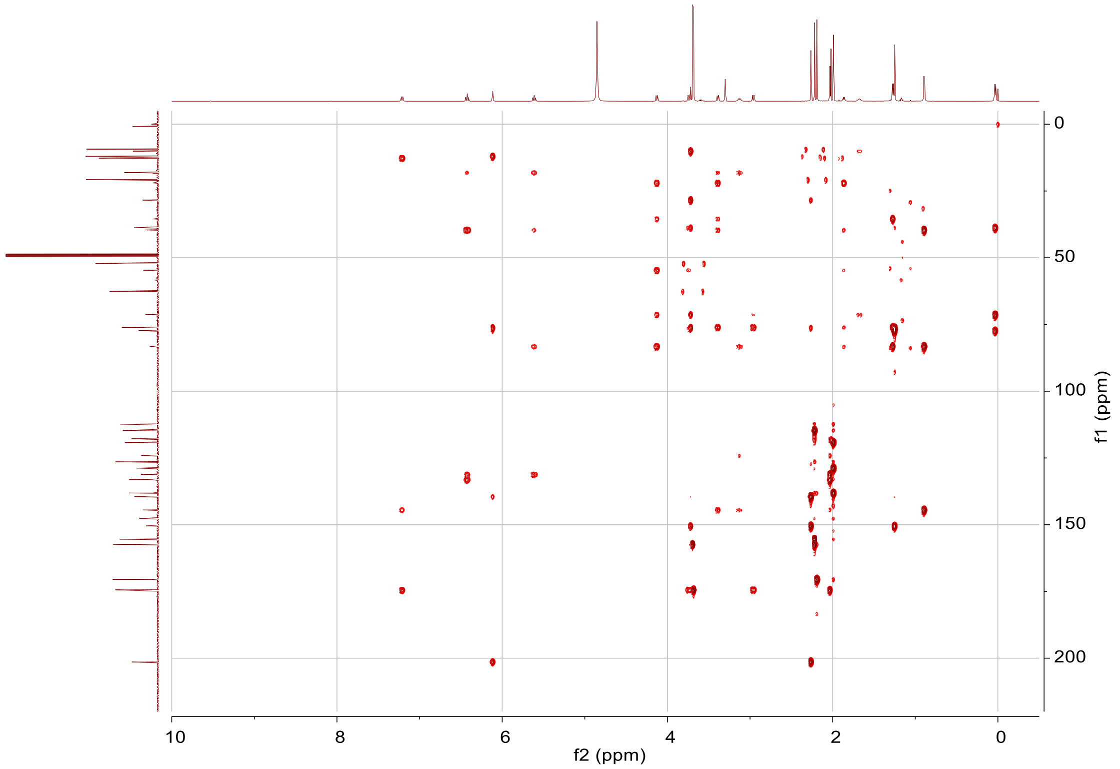

Supplementary Fig. 13 HMBC spectrum (600 MHz, CH_3OH-*d*$_4$) of 6-methoxy-streptovaricin C (2).

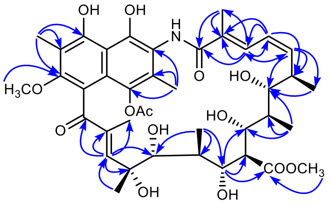

6-methoxy-streptovaricin C (**2**)

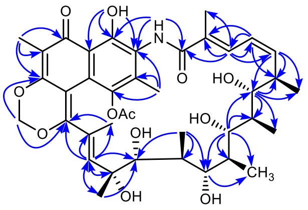

Streptovaricin H (**3**)

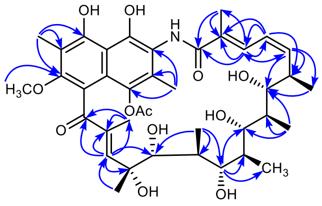

6-methoxy-streptovaricin H (**4**)

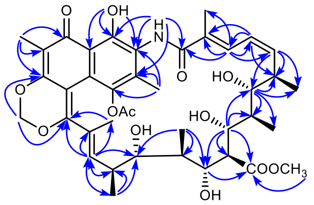

Streptovaricin D (**5**)

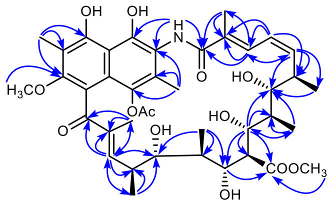

6-methoxy-streptovaricin D (**6**)

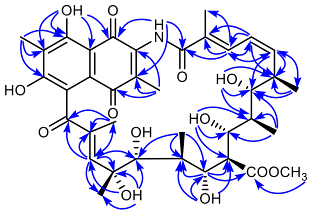

Damavaricin C (**7**)

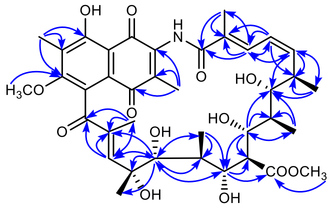

6-methoxy-damavaricin C (**8**)

Supplementary Fig. 14 Crucial HMBC correlations of streptovaricin derivatives 2-8.

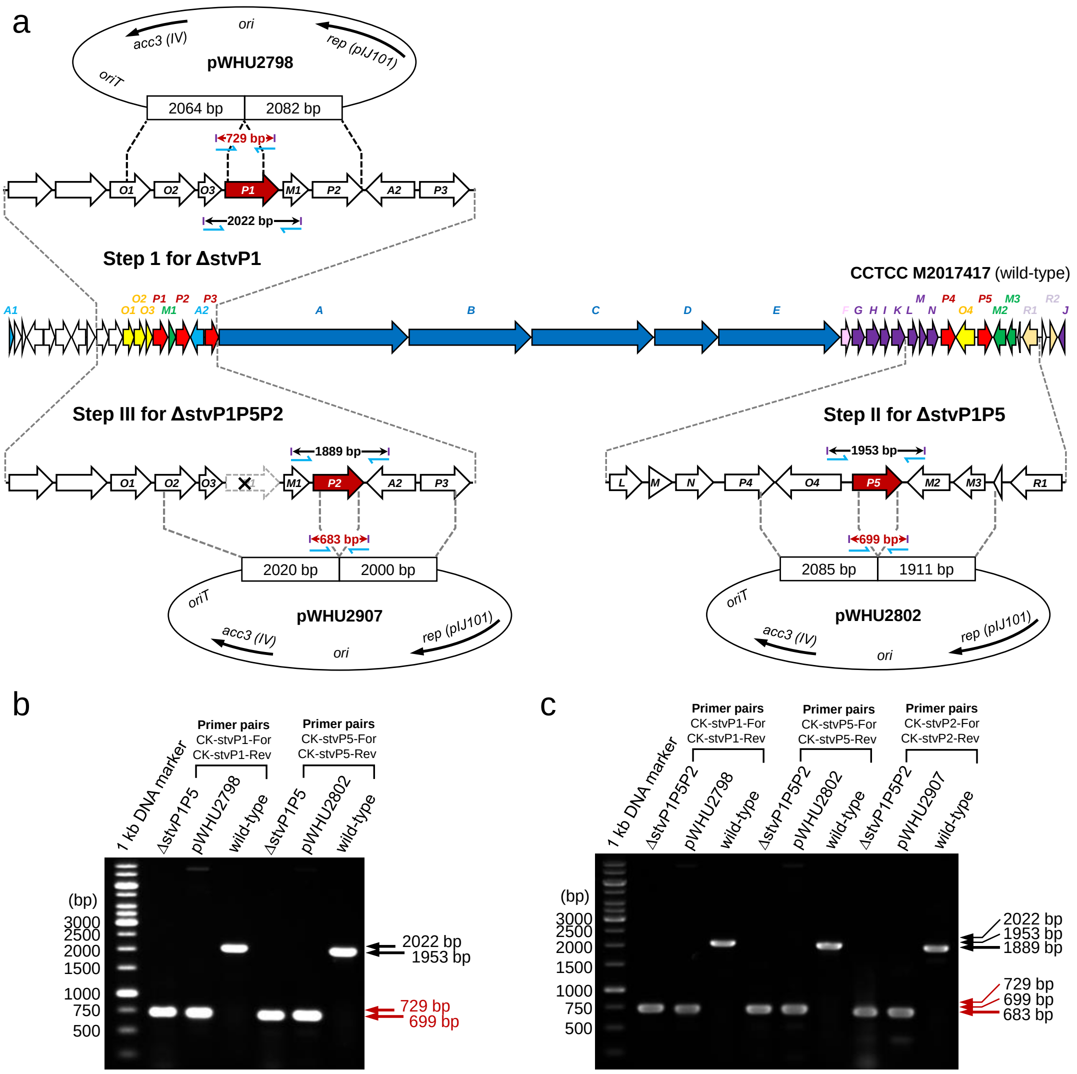

Supplementary Fig. 15 Scheme of mutants ΔstvP1P5 and ΔstvP1P5P2 construction and verification. (**a**) Representative scheme for double gene in-frame deletion of *stvP1* and *stvP5*, and triple gene in-frame deletion of *stvP1*, *stvP5* and *stvP2*. The primers used for checking the mutant is indicated by blue half arrows. Agarose gel electrophoresis of mutant verification for ΔstvP1P5 (**b**) and ΔstvP1P5P2 (**c**) by PCR. The results in **b** and **c** are representative of three independent experiments. Source data are provided as a Source Data file.

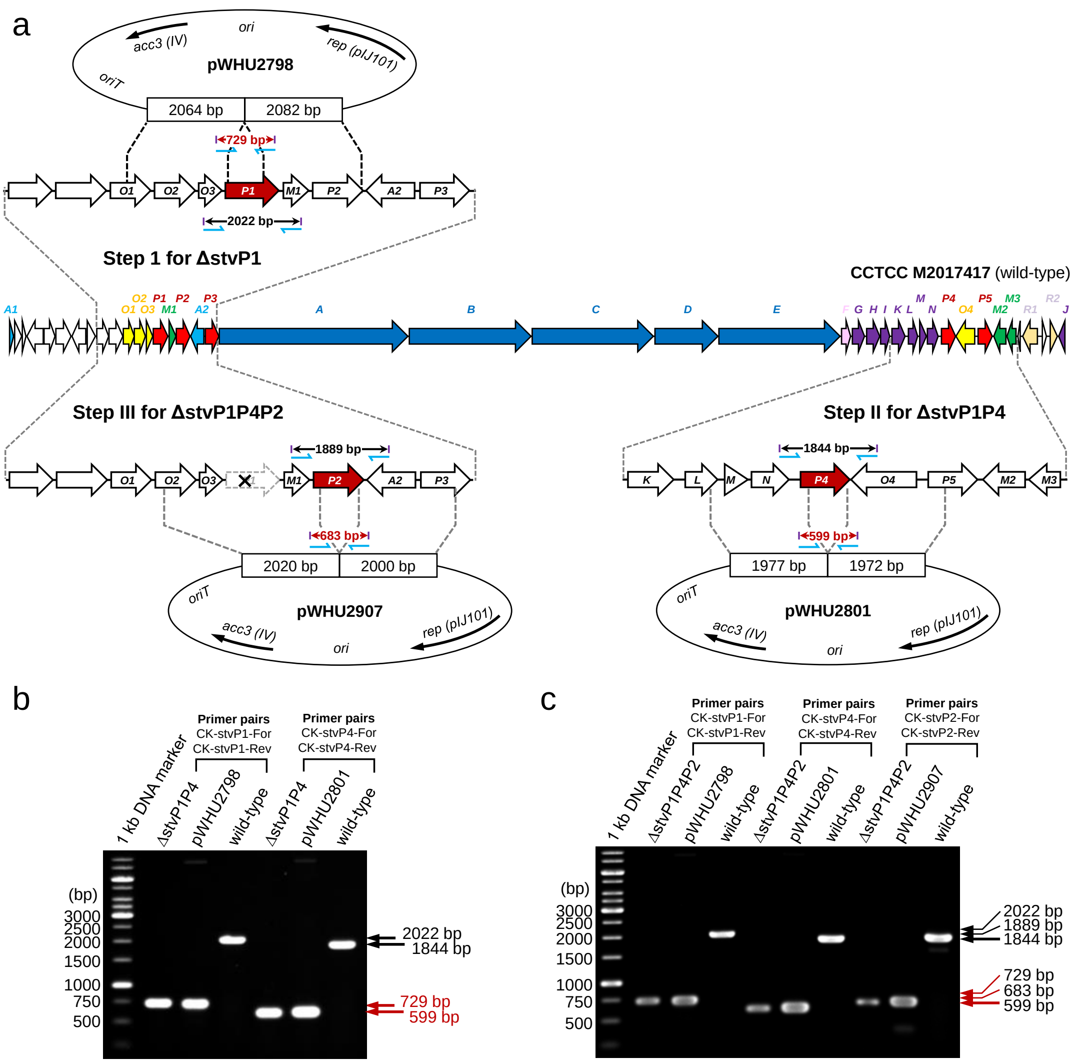

Supplementary Fig. 16 Scheme of mutants ΔstvP1P4 and ΔstvP1P4P2 construction and verification. (**a**) Representative scheme for double gene in-frame deletion of *stvP1* and *stvP4*, and triple gene in-frame deletion of *stvP1*, *stvP4* and *stvP2*. The primers used for checking the mutant is indicated by blue half arrows. Agarose gel electrophoresis of mutant verification for ΔstvP1P4 (**b**) and ΔstvP1P4P2 (**c**) by PCR. The results in **b** and **c** are representative of three independent experiments. Source data are provided as a Source Data file.

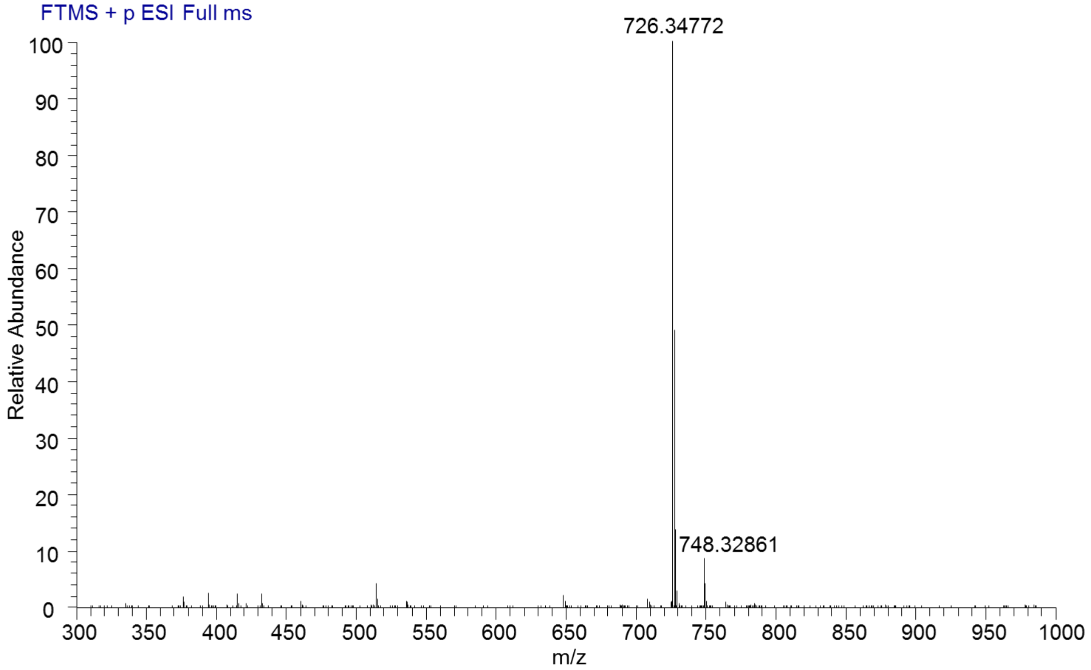

Supplementary Fig.17 ESI-HRMS spectrum of streptovaricin H (3).

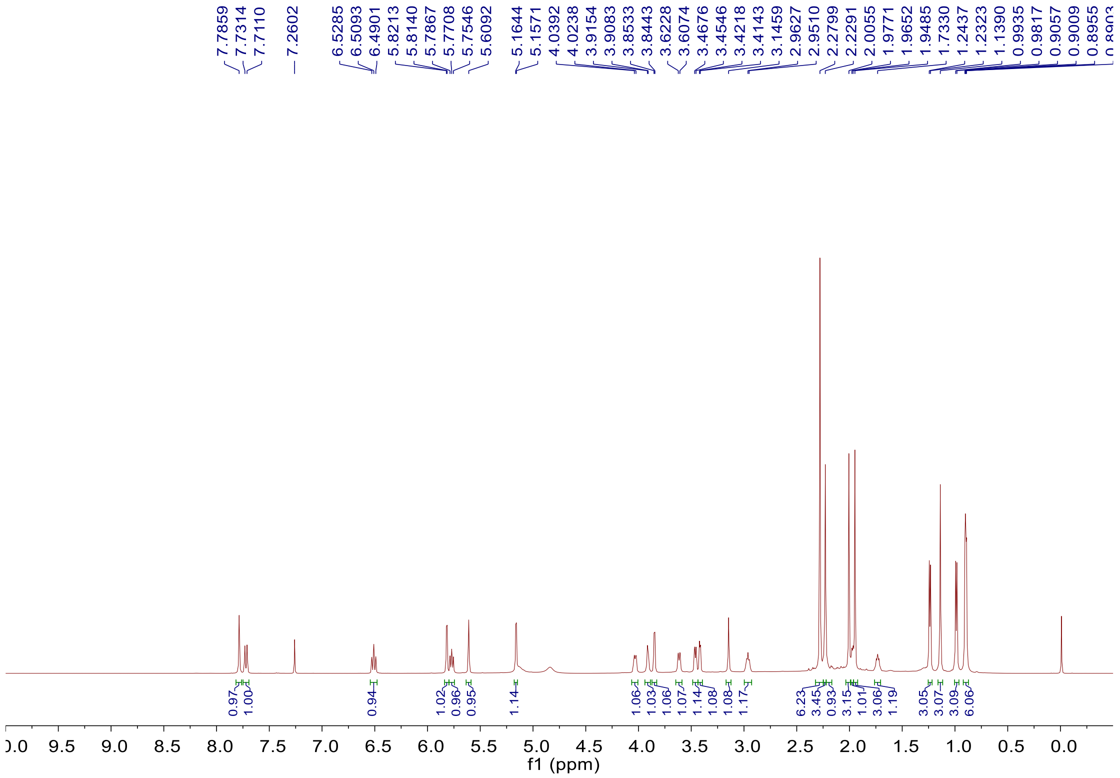

Supplementary Fig. 18 ^{1}H NMR spectrum (600 MHz, $CHCl_3$-d_1) of streptovaricin H (3).

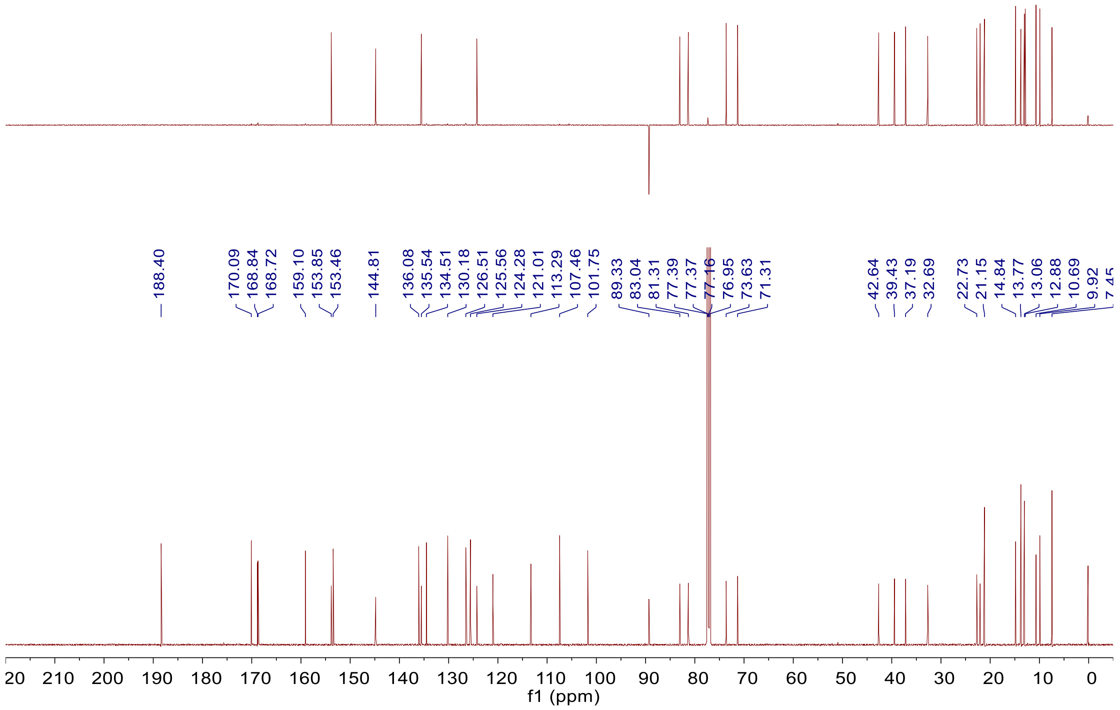

Supplementary Fig. 19 ^{13}C NMR and DEPT135 spectrum (150 MHz, $CHCl_3$-d_1) of streptovaricin H (3).

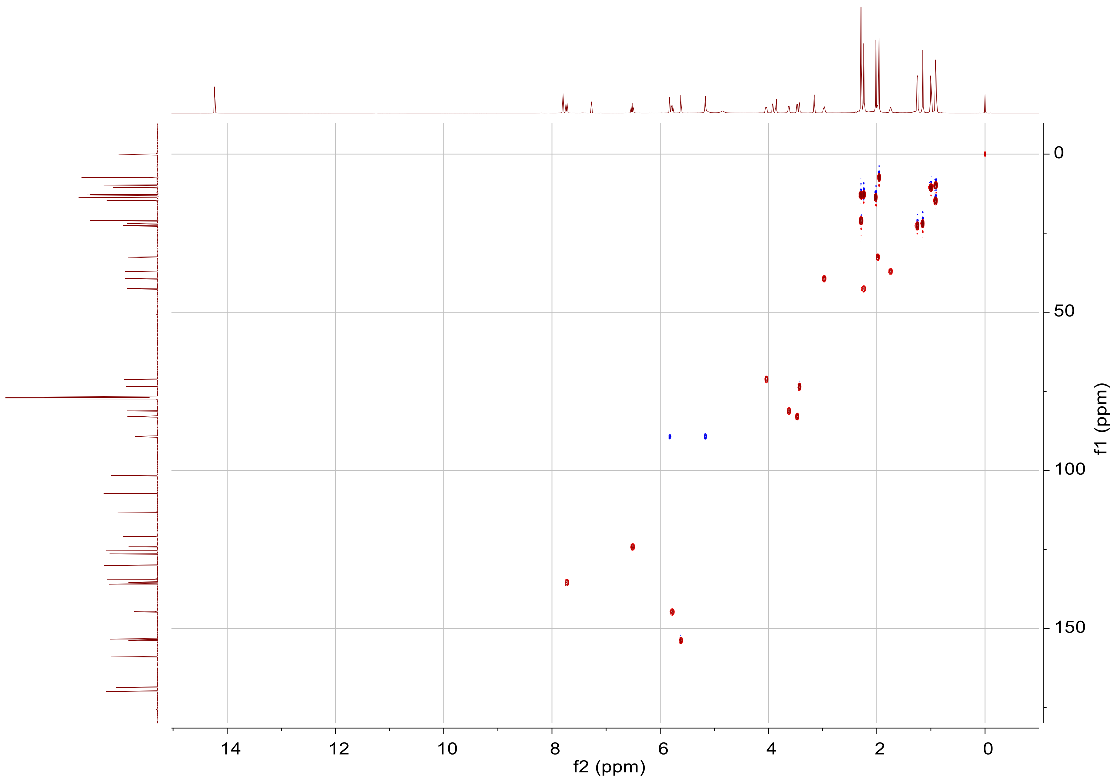

Supplementary Fig. 20 HSQC spectrum (600 MHz, $CHCl_3$-d_1) of streptovaricin H (3).

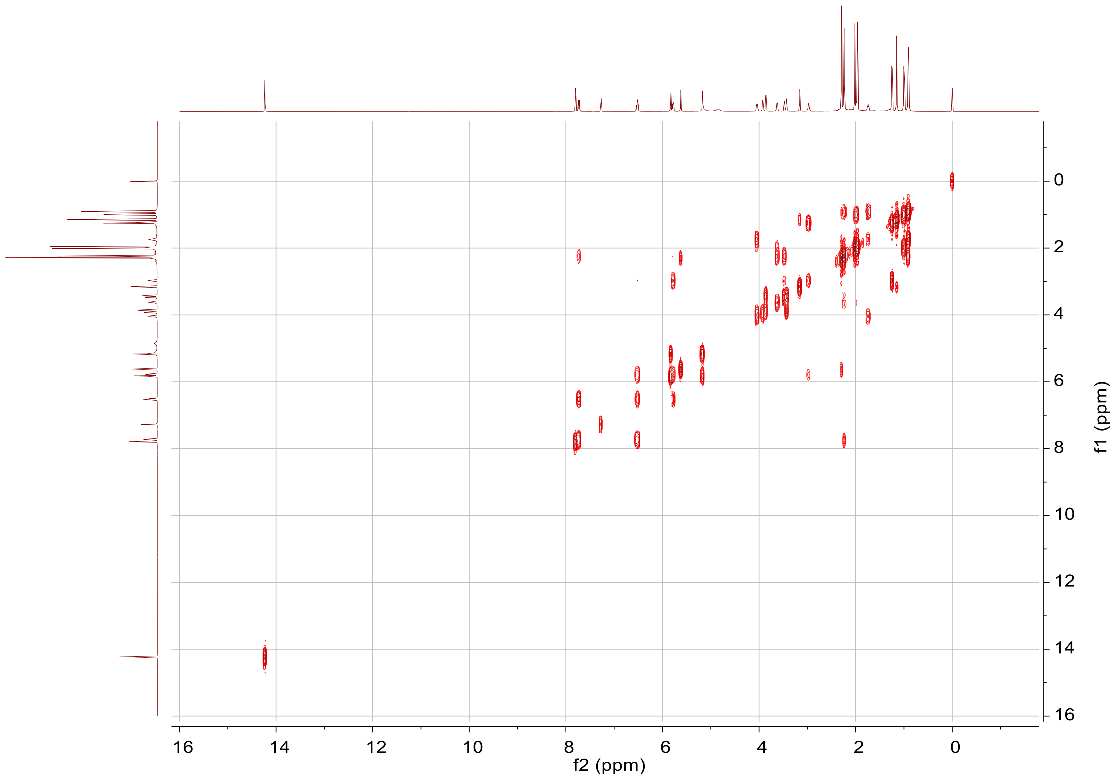

Supplementary Fig. 21 ^1H-^1H COSY spectrum (600 MHz, $CHCl_3$-d_1) of streptovaricin H (3).

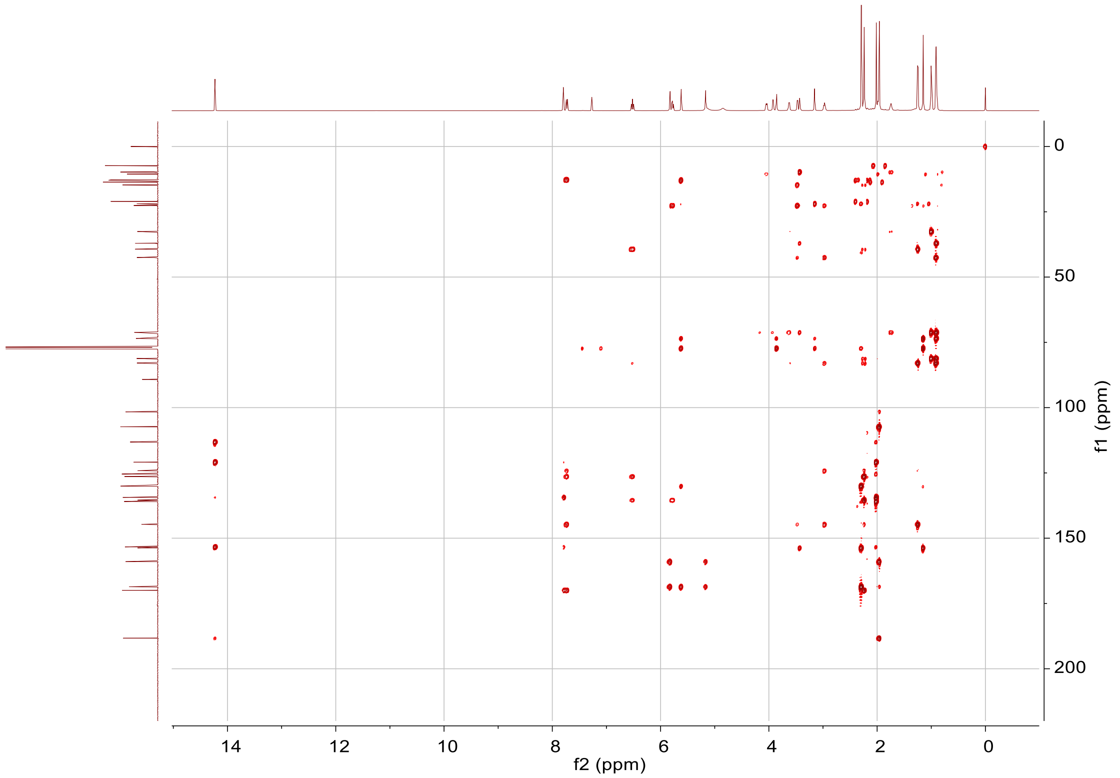

Supplementary Fig. 22 HMBC spectrum (600 MHz, $CHCl_3$-d_1) of streptovaricin H (3).

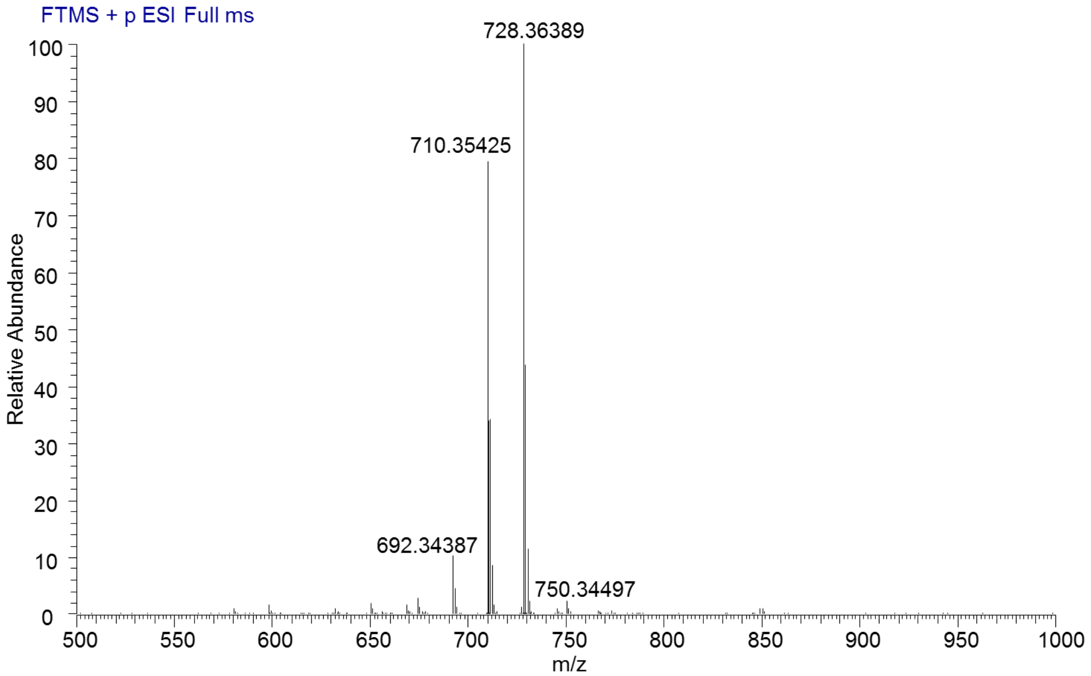

Supplementary Fig. 23 ESI-HRMS spectrum of 6-methoxy-streptovaricin H (4).

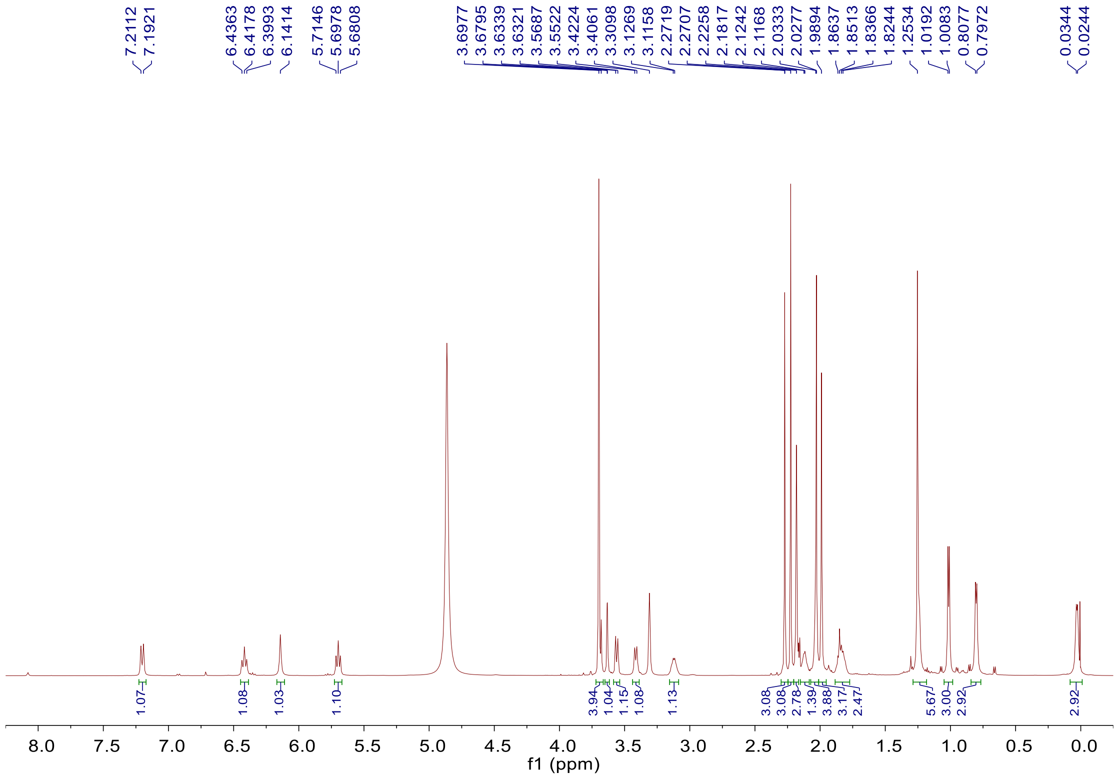

Supplementary Fig. 24 ^{1}H NMR spectrum (600 MHz, CH_3OH-d_4) of 6-methoxy-streptovaricin H (4).

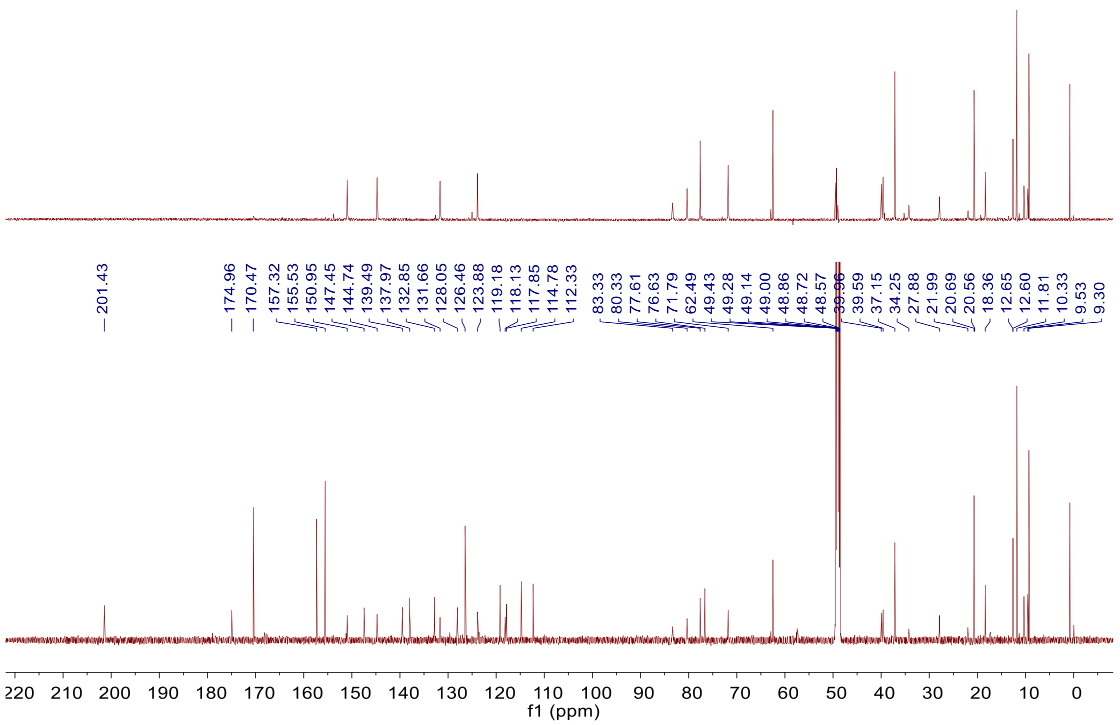

Supplementary Fig. 25 ^{13}C NMR and DEPT135 spectrum (600 MHz, CH_3OH-d_4) of 6-methoxy-streptovaricin H (4).

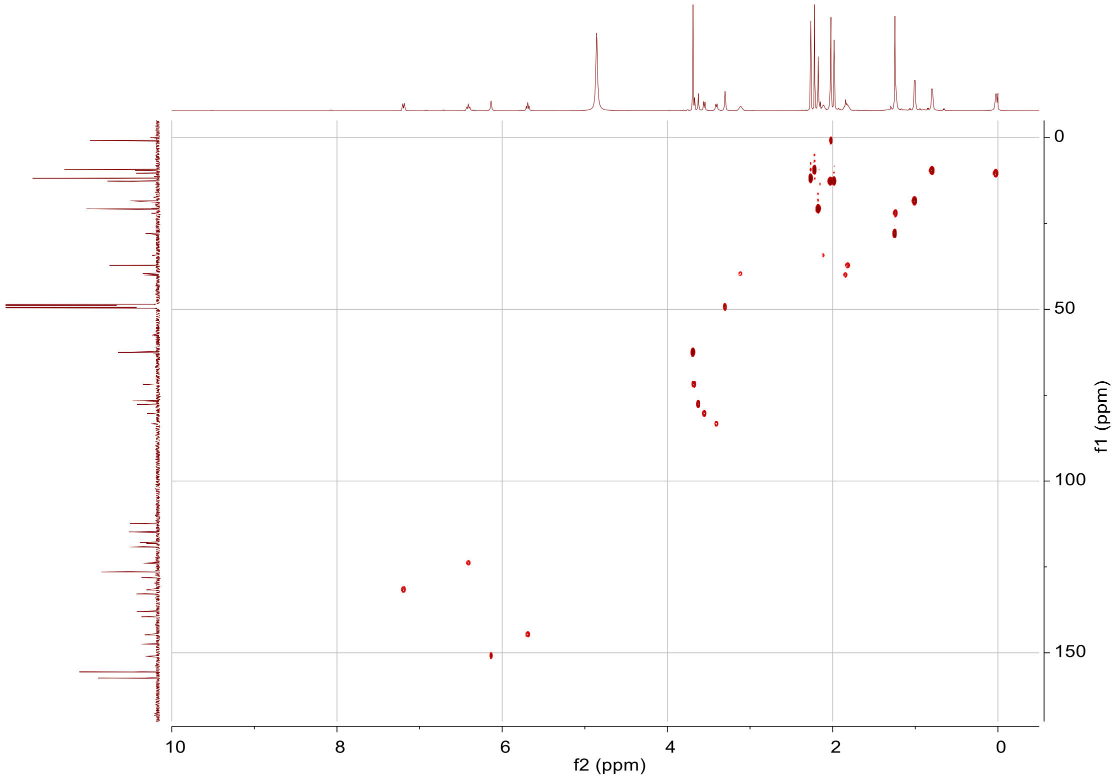

Supplementary Fig. 26 HSQC spectrum (600 MHz, CH_3OH-d_4) of 6-methoxy-streptovaricin H (4).

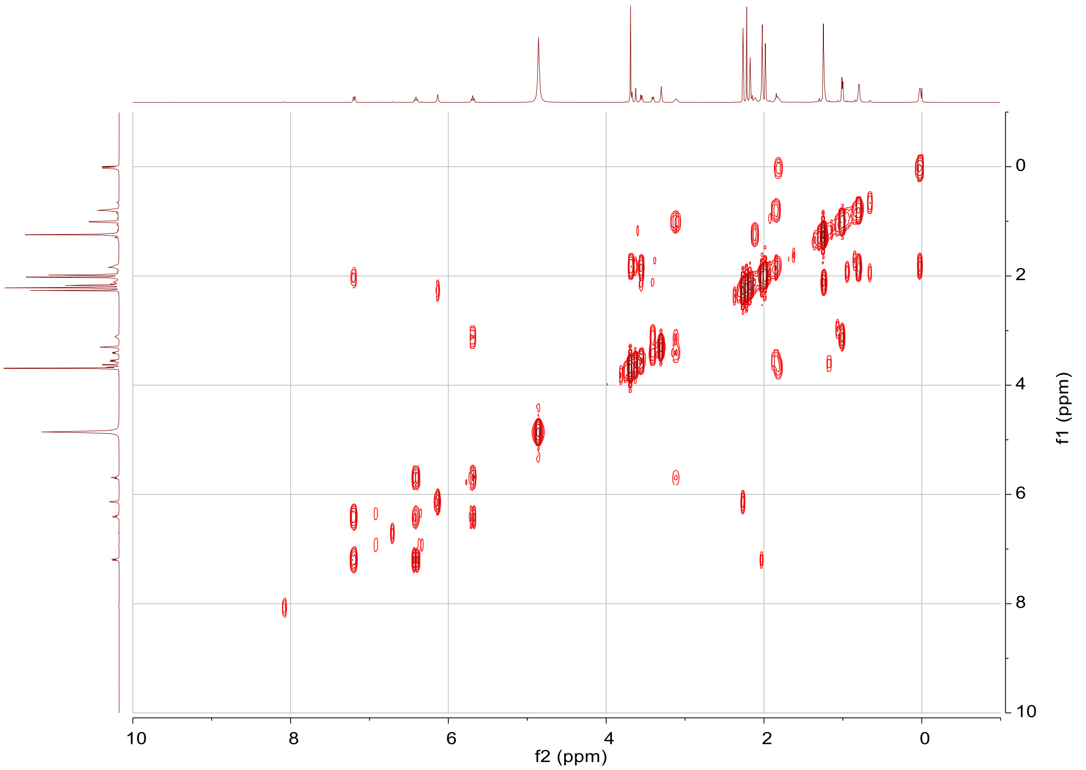

Supplementary Fig. 27 ^{1}H-^{1}H COSY spectrum (600 MHz, CH_3OH-d_4) of 6-methoxy-streptovaricin H (4).

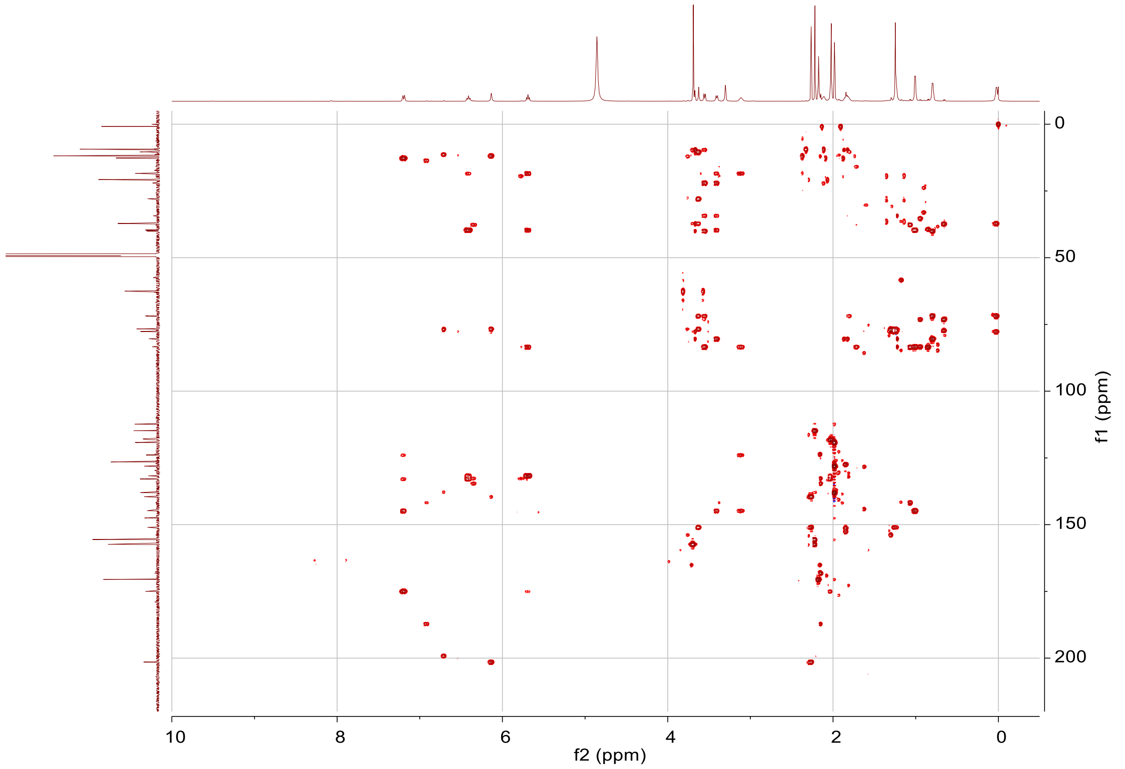

Supplementary Fig. 28 HMBC spectrum (600 MHz, CH_3OH-d_4) of 6-methoxy-streptovaricin H (4).

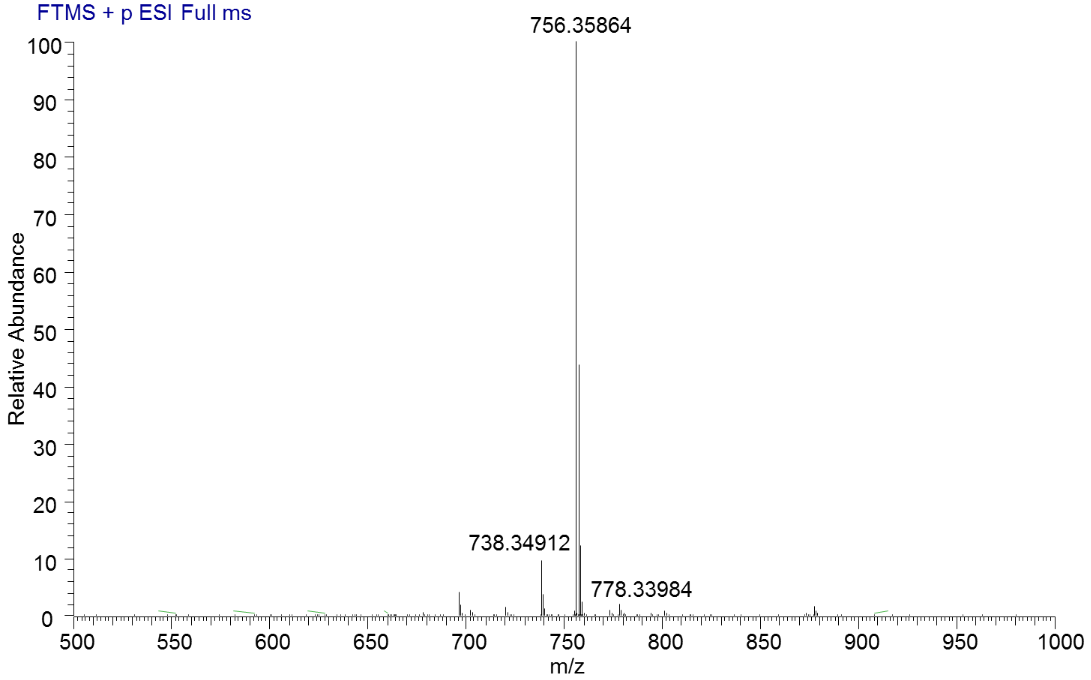

Supplementary Fig. 29 ESI-HRMS spectrum of streptovaricin D (5).

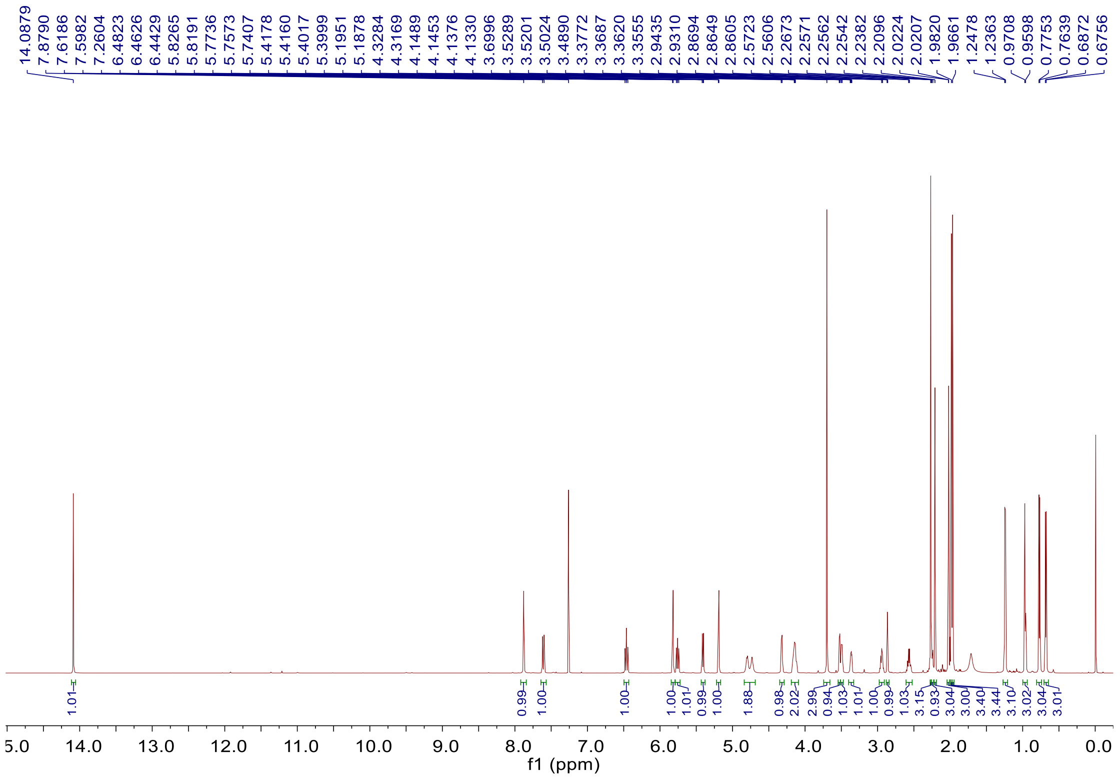

Supplementary Fig. 30 ^{1}H NMR spectrum (600 MHz, $CHCl_3$-d_1) of streptovaricin D (5).

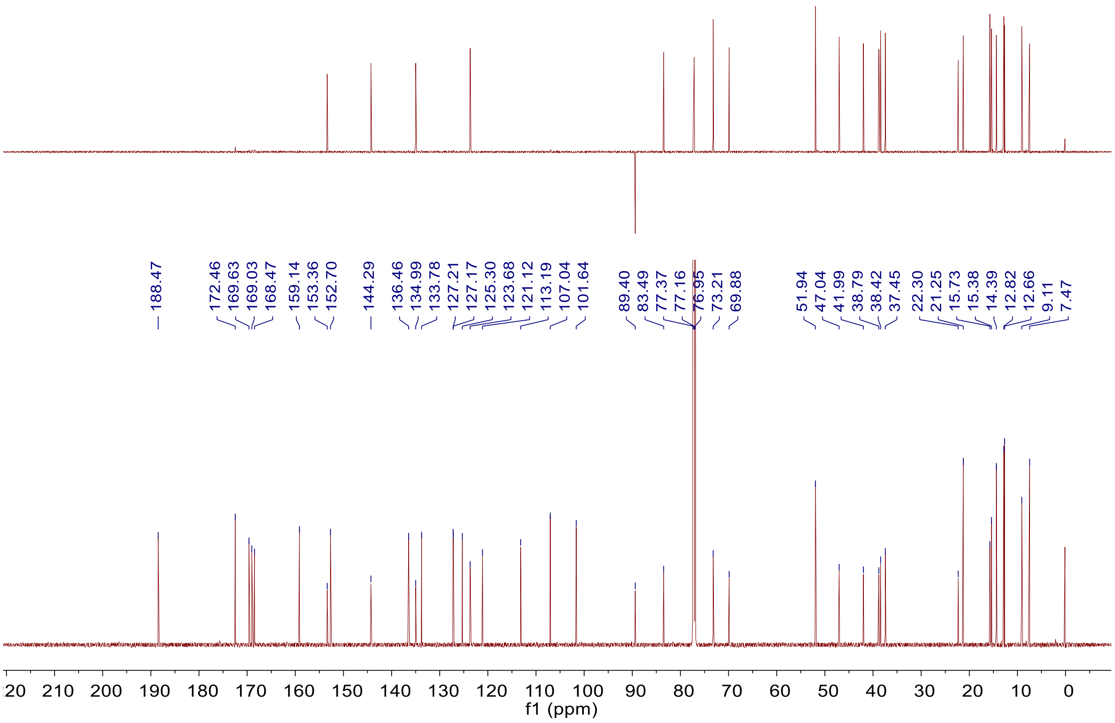

Supplementary Fig. 31 ^{13}C NMR and DEPT135 spectrum (150 MHz, $CHCl_3$-d_1) of streptovaricin D (5).

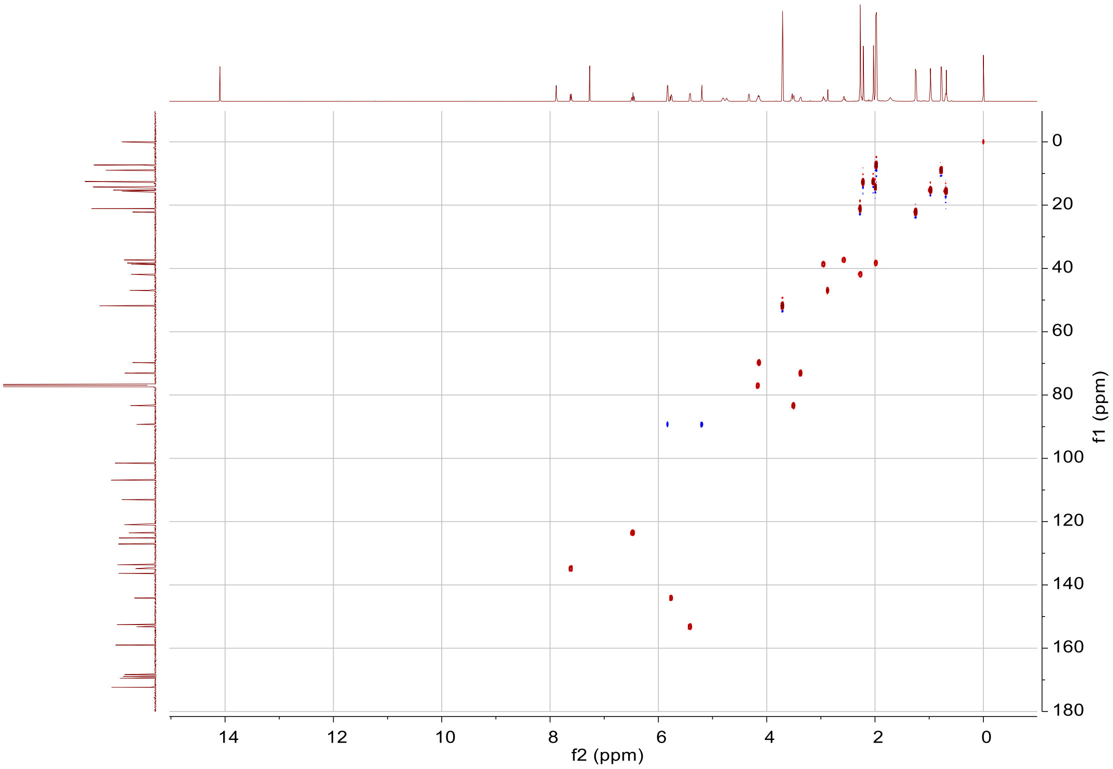

Supplementary Fig. 32 HSQC spectrum (600 MHz, $CHCl_3$-d_1) of streptovaricin D (6).

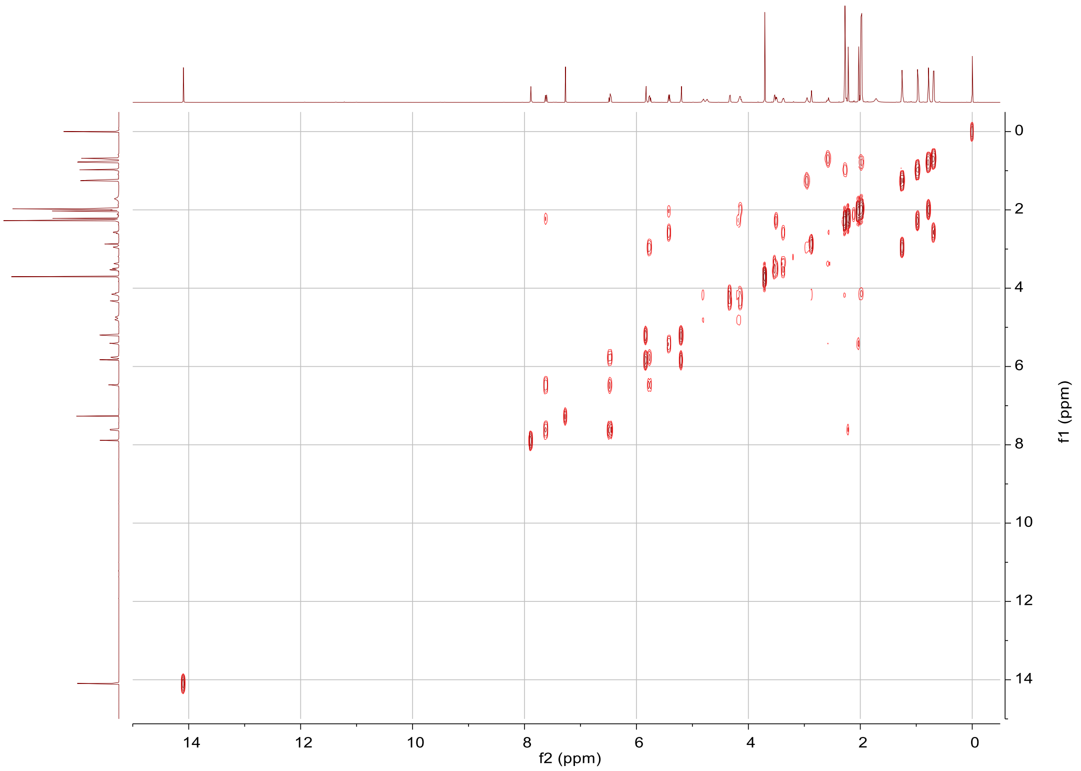

Supplementary Fig. 33 ^{1}H-^{1}H COSY spectrum (600 MHz, $CHCl_3$-d_1) of streptovaricin D (5).

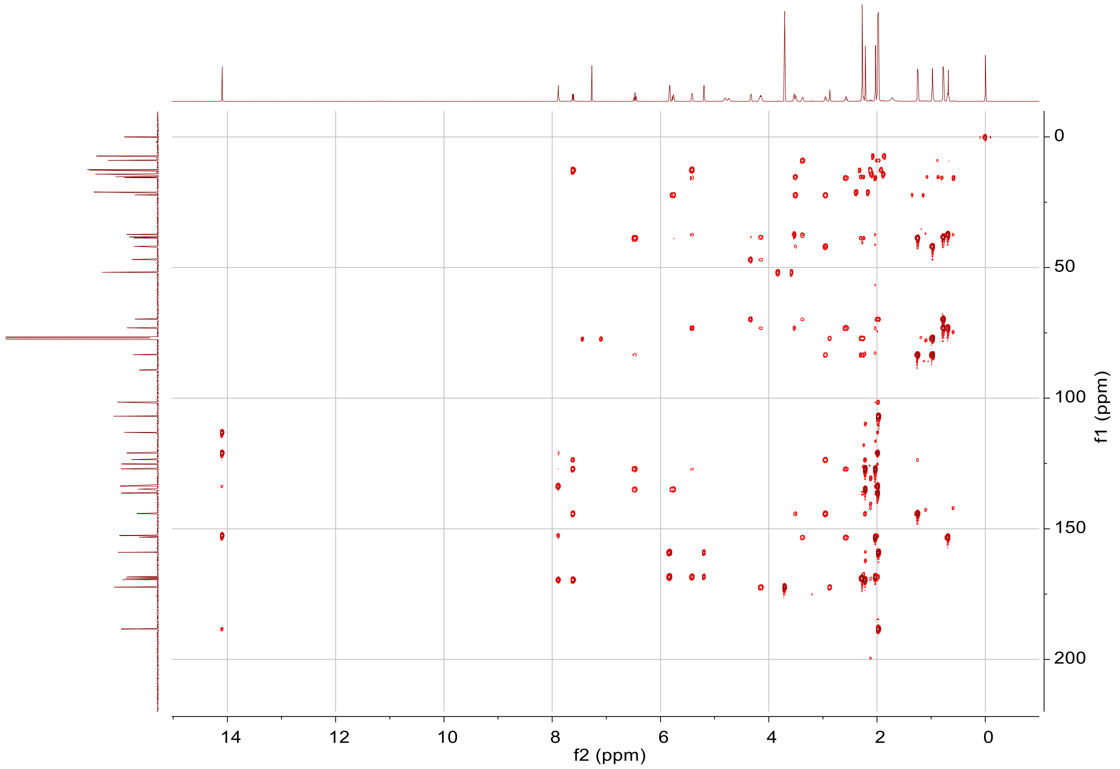

Supplementary Fig. 34 HMBC spectrum (600 MHz, $CHCl_3$-d_1) of streptovaricin D (5).

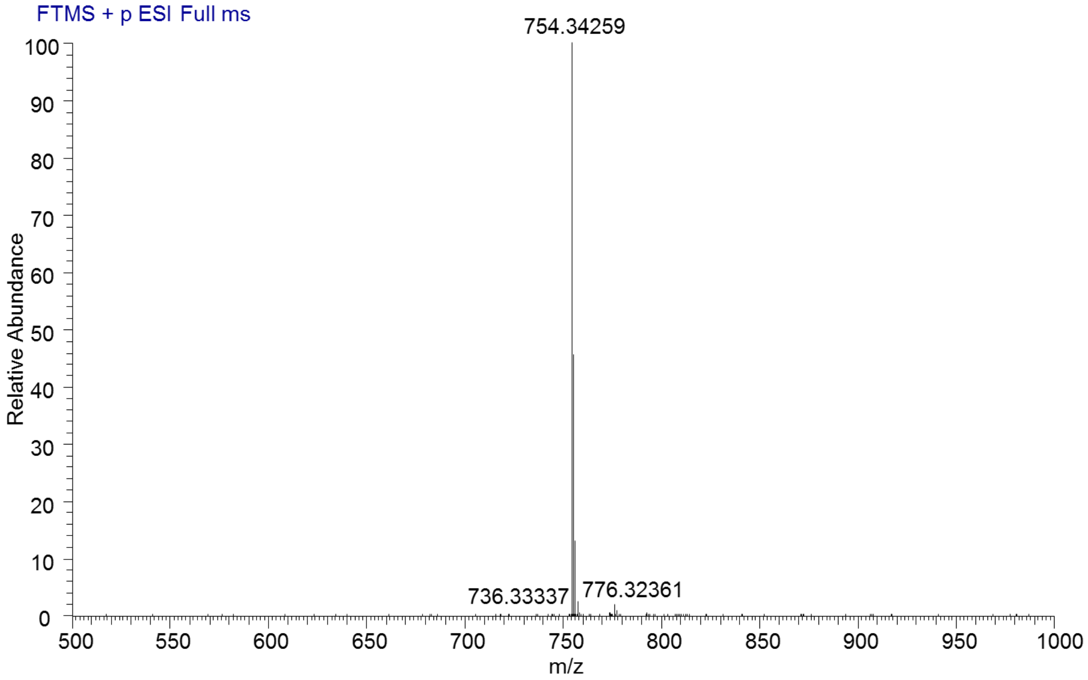

Supplementary Fig. 35 ESI-HRMS spectrum of 6-methoxy-streptovaricin D (6).

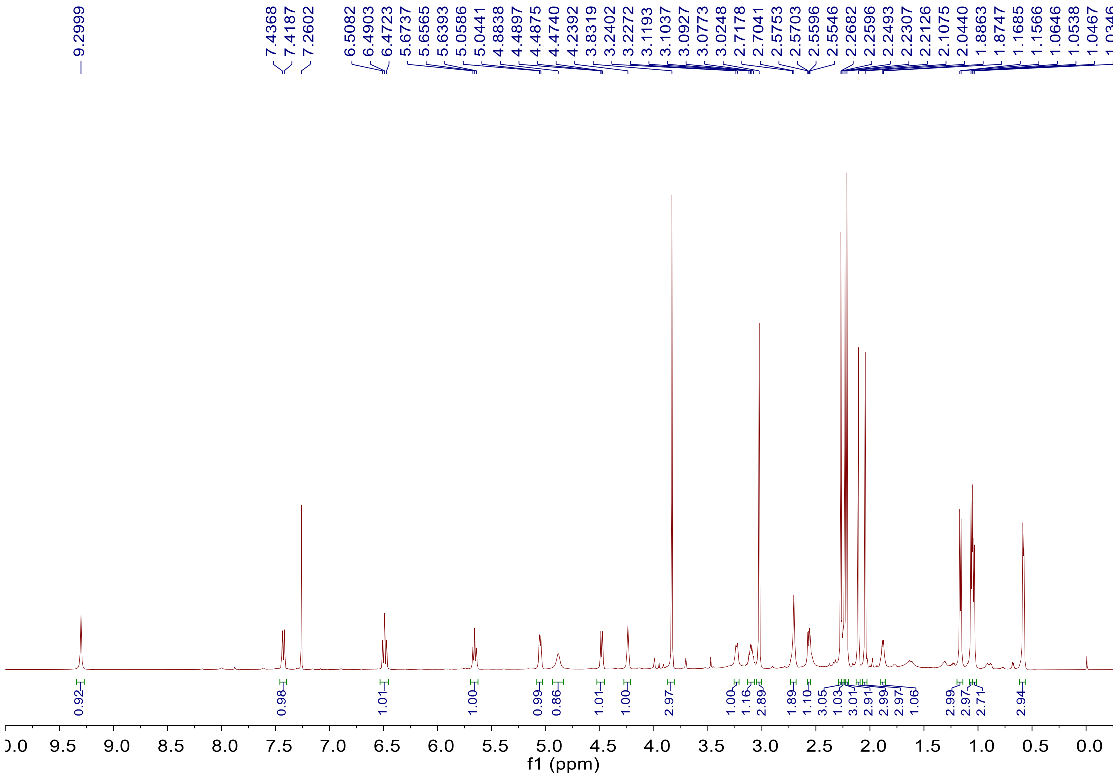

Supplementary Fig. 36 ^{1}H NMR spectrum (600 MHz, $CHCl_3$-d_1) of 6-methoxy-streptovaricin D (6).

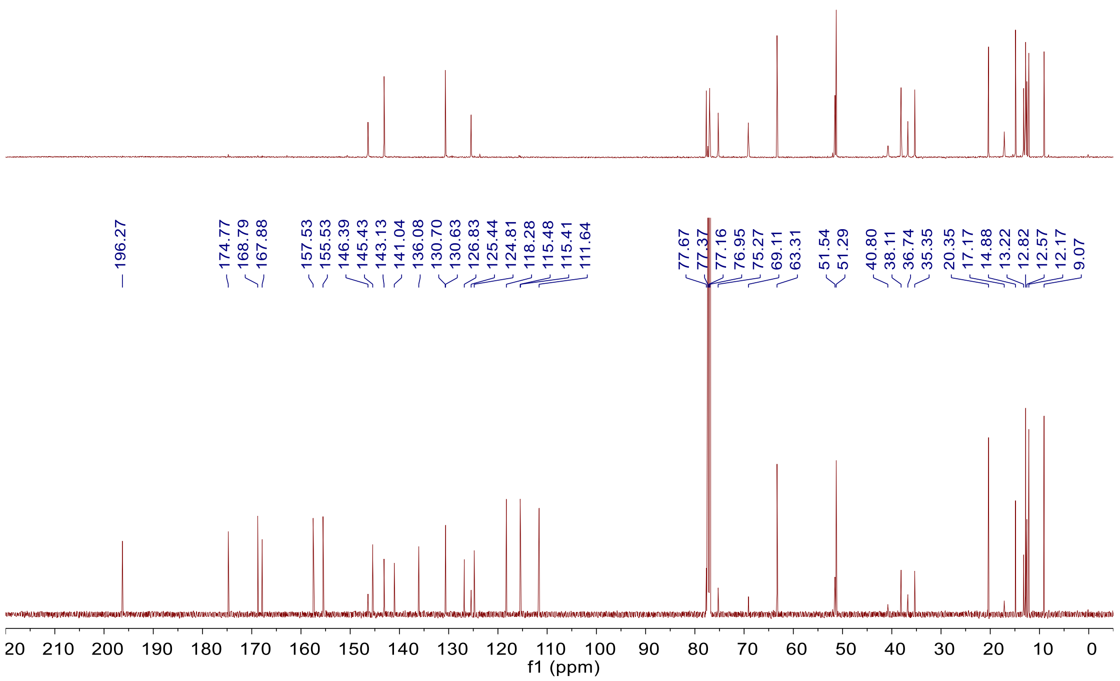

Supplementary Fig. 37 ^{13}C NMR and DEPT135 spectrum (150 MHz, $CHCl_3$-*d*$_1$) of 6-methoxy-streptovaricin D (6).

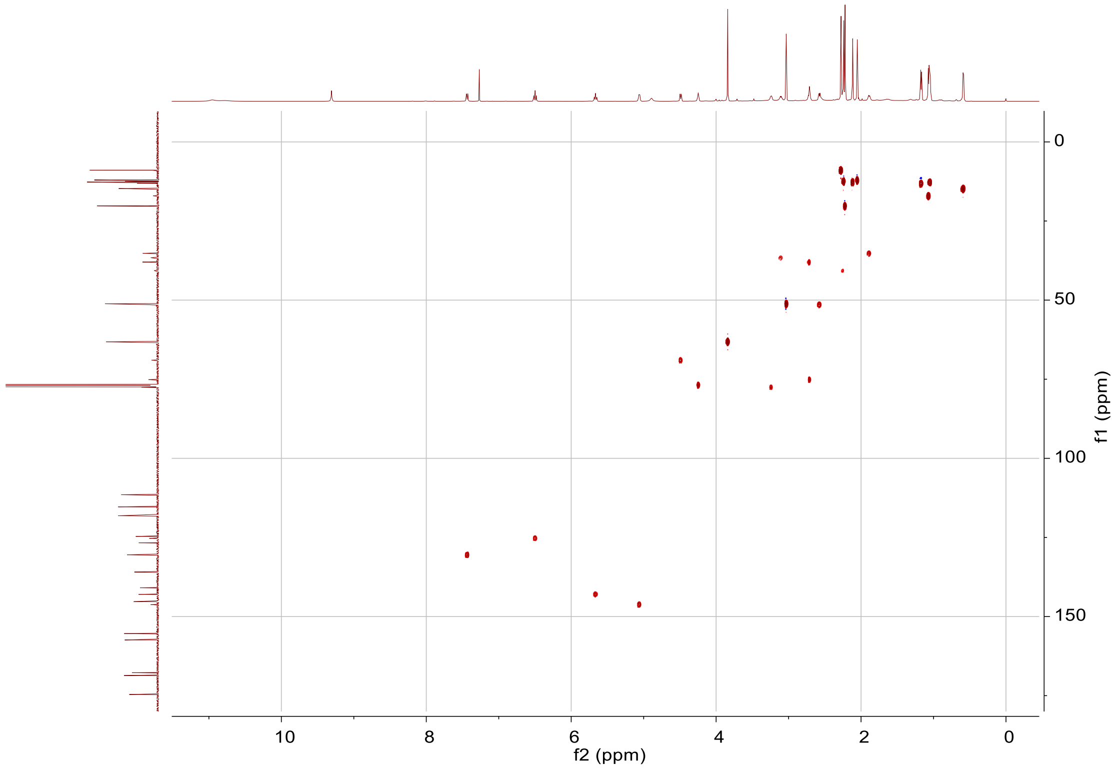

Supplementary Fig. 38 HSQC spectrum (600 MHz, $CHCl_3$-*d*$_1$) of 6-methoxy-streptovaricin D (6).

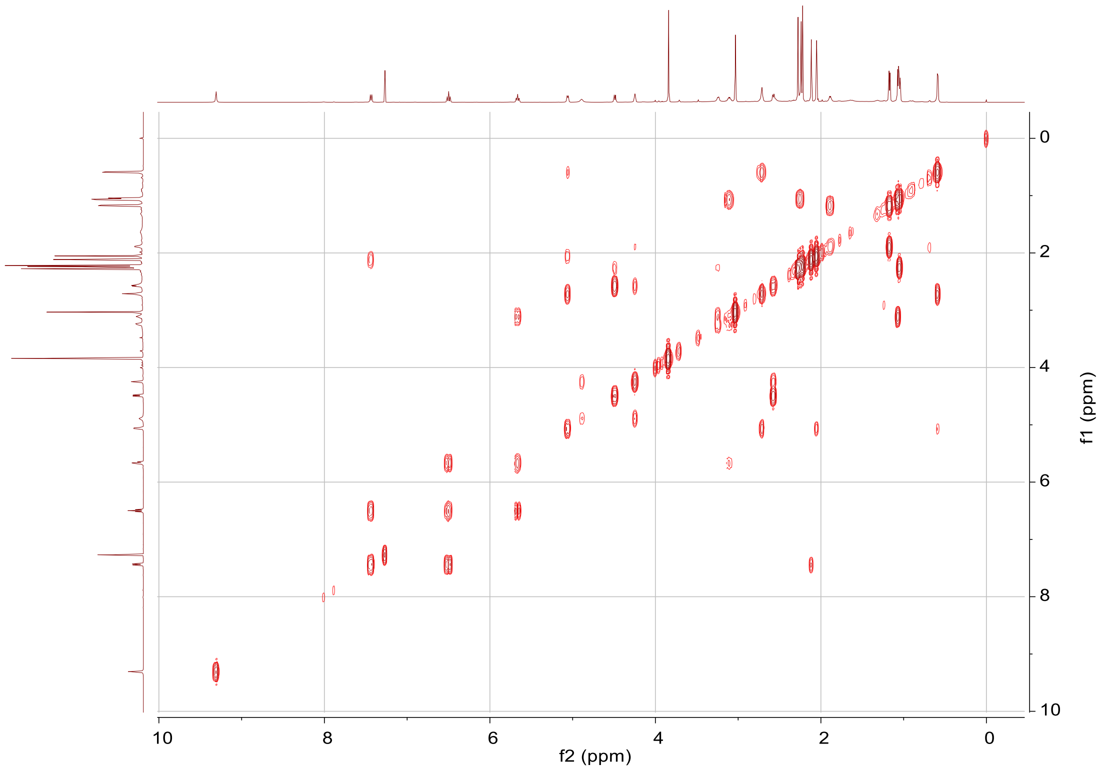

Supplementary Fig. 39 ^{1}H-^{1}H COSY spectrum (600 MHz, $CHCl_3$-*d*$_1$) of 6-methoxy-streptovaricin D (6).

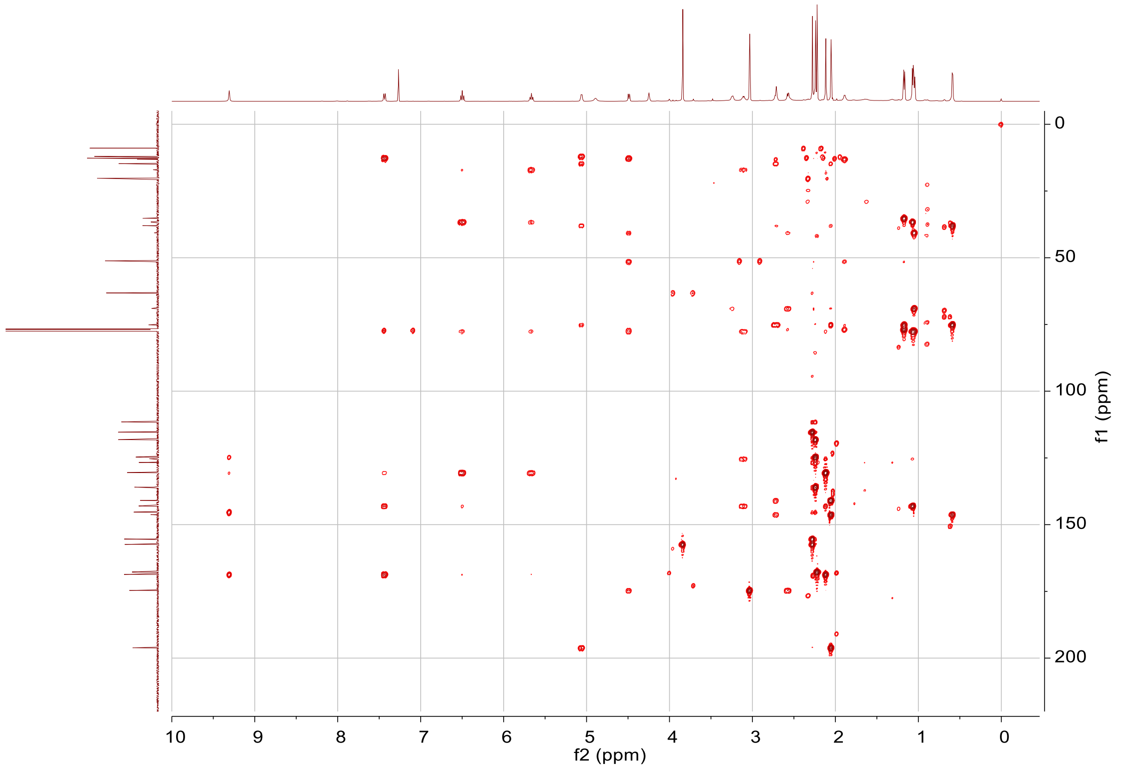

Supplementary Fig. 40 HMBC spectrum (600 MHz, $CHCl_3$-*d*$_1$) of 6-methoxy-streptovaricin D (6).

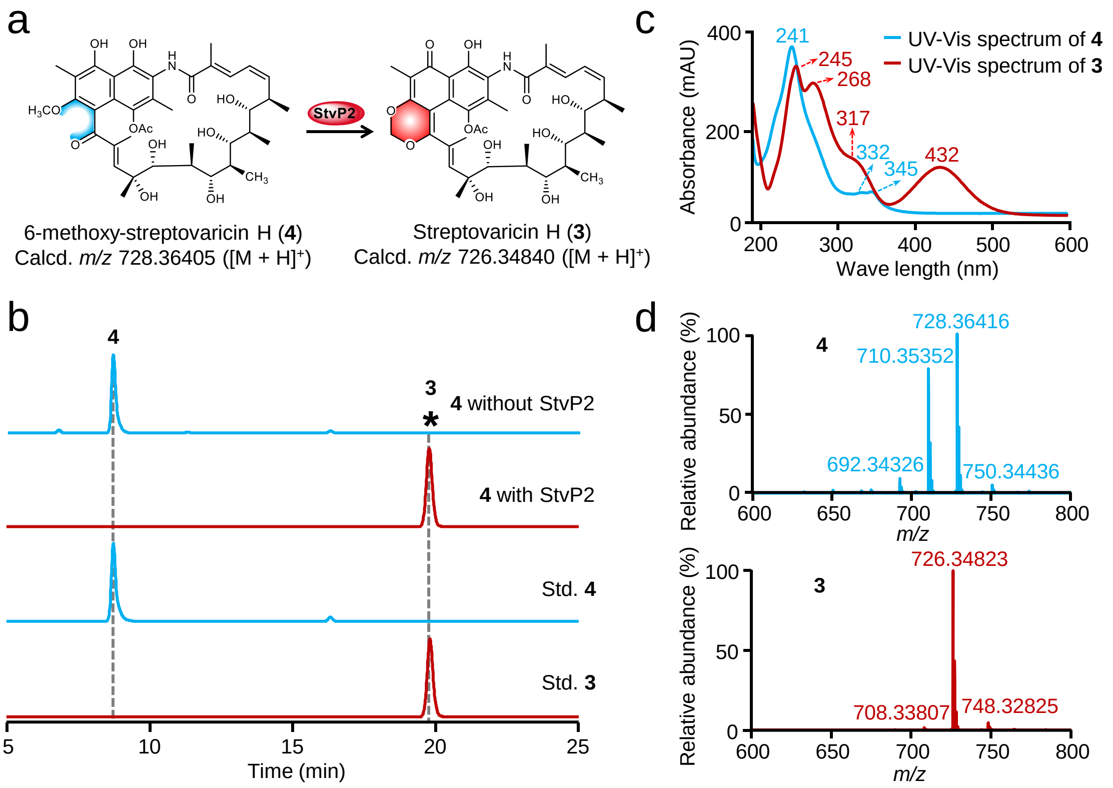

Supplementary Fig. 41 ***In vitro*** **enzymatic conversion of 6-methoxy-streptovaricin H (4) to form streptovaricin H (3) catalyzed by StvP2.** (**a**) Biochemical reaction formula of StvP2-catalyzed conversion of **4** to **3**. (**b**) LC-ESI-HRMS analysis of enzymatic conversion of **4** to **3** by StvP2. The asterisk indicated no component corresponding to **3** was detected. (**c**) UV/Vis spectra of **4** and **3**. (**d**) ESI-HRMS spectra of **4** (calcd. *m/z* 728.36405 $[M + H]^+$; 750.34600 $[M + Na]^+$; 710.35349 $[M + H - H_2O]^+$; 692.34292 $[M + H - 2H_2O]^+$) and **3** (calcd. *m/z* 726.34840 $[M + H]^+$; 748.33035 $[M + Na]^+$; 708.33784 $[M + H - H_2O]^+$) extracted from total ion chromatography. The pure compounds **4** and **3** identified by NMR were used as standards (Std. **4** and Std. **3**). Experiments in **b**-**d** are representative of three independent experiments.

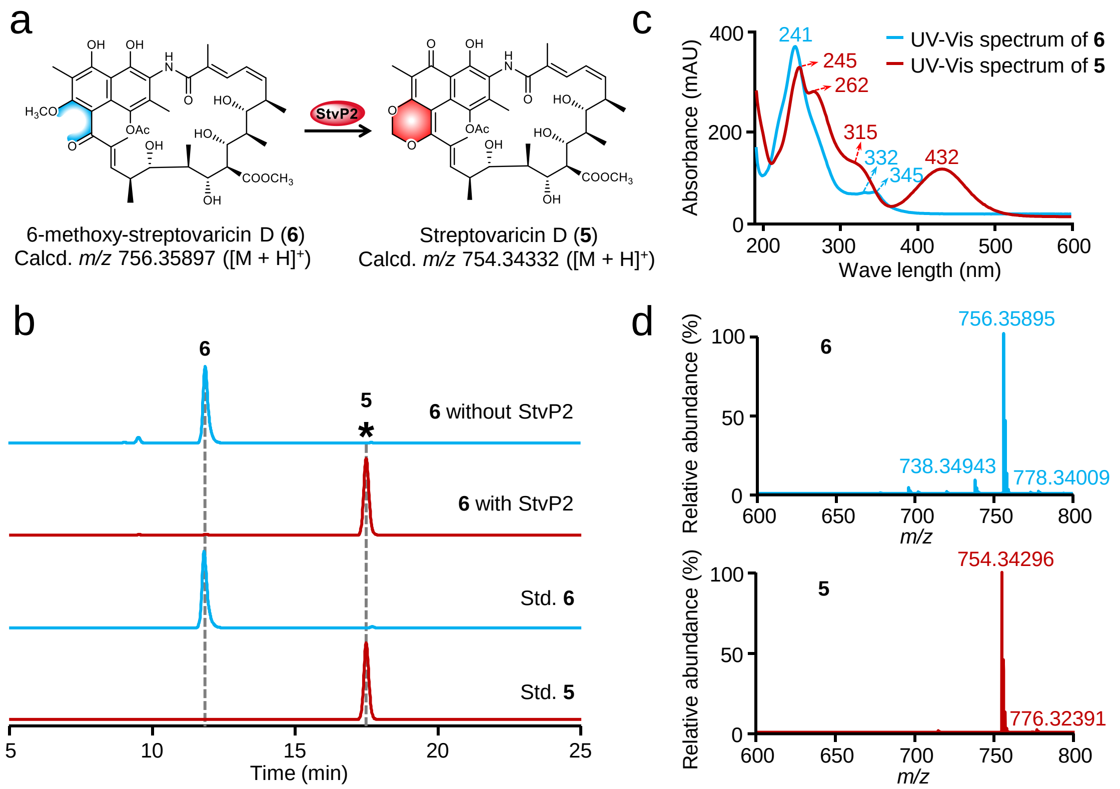

Supplementary Fig. 42 ***In vitro*** **enzymatic conversion of 6-methoxy-streptovaricin D (6) to form streptovaricin D (5) catalyzed by StvP2.** (**a**) Biochemical reaction formula of StvP2-catalyzed conversion of **6** to **5**. The asterisk indicated no component corresponding to **5** was detected. (**b**) LC-ESI-HRMS analysis of enzymatic conversion of **6** to **5** by StvP2. (**c**) UV/Vis spectra of **6** and **5**. (**d**) ESI-HRMS spectra of **6** (calcd. *m/z* 756.35897 [M + H]$^+$; 778.34091 [M + Na]$^+$; 738.34840 [M + H – H_2O]$^+$) and **5** (calcd. *m/z* 754.34332 [M + H]$^+$; 776.32526 [M + Na]$^+$) extracted from total ion chromatography. The pure compounds **6** and **5** identified by NMR were used as standards (Std. **6** and Std. **5**). Experiments in **b**-**d** are representative of three independent experiments.

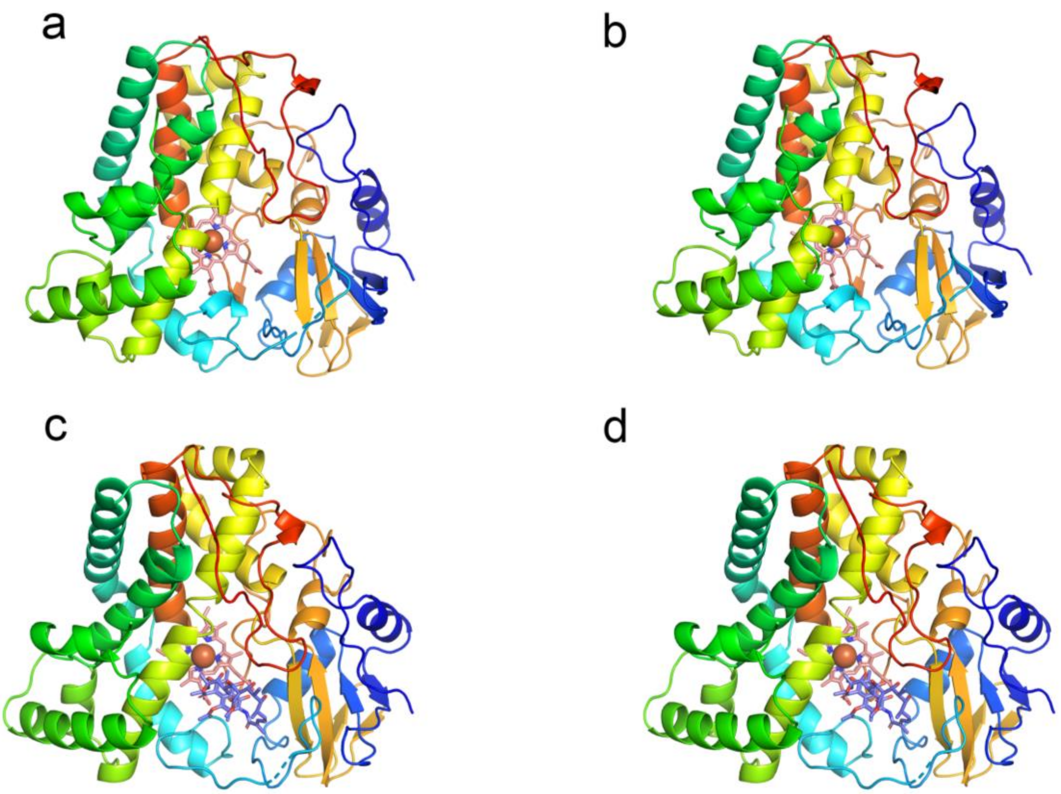

Supplementary Fig. 43 Stereo view of the StvP2 and substrate 2-bound StvP2 structures. (**a**) Left-eye image of stereo view of substrate-free StvP2. (**b**) Right-eye image of stereo view of substrate-free StvP2. (**c**) Left-eye image of stereo view of substrate **2**-bound StvP2. (**d**) Right-eye image of stereo view of substrate **2**-bound StvP2.The structure is colored in rainbow. Heme is colored in salmon and its Fe^{3+} ion is colored in orange. The substrate **2** is colored in blue.

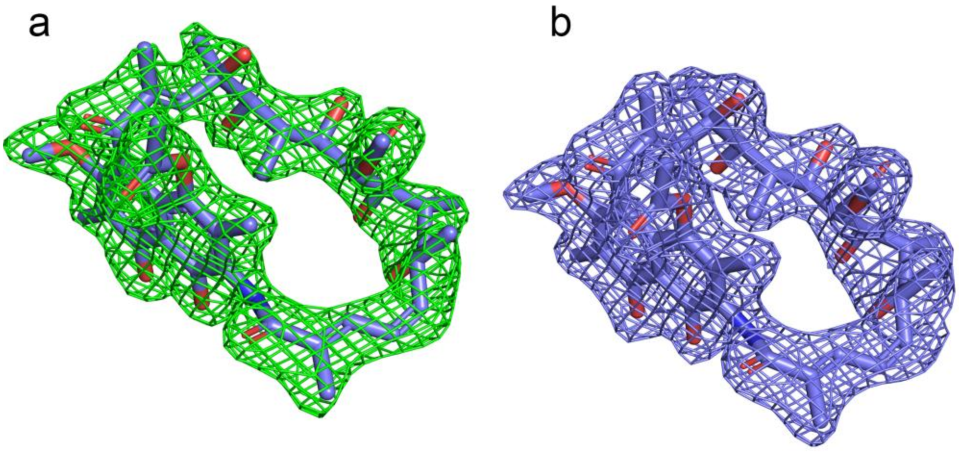

Supplementary Fig. 44 Electron density map of substrate 2. (**a**) The F_O-F_C map was calculated without substrate **2** and contoured at 2.5σ. The **2** is colored in blue. (**b**) The $2F_O$-F_C map was calculated after refinement with substrate **2** molecule and the map is contoured at 1.0σ.

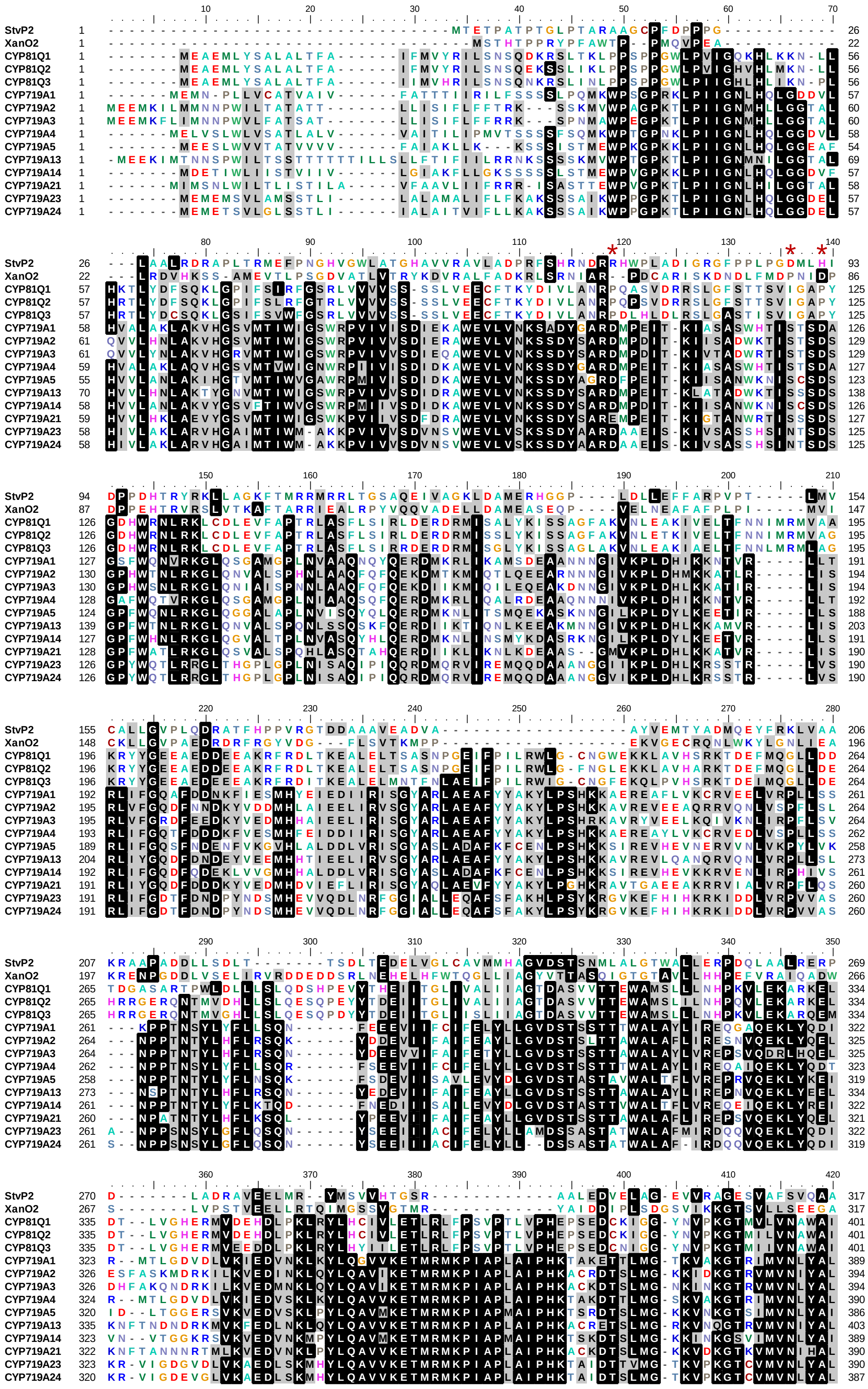
StvP2
XanO2
CYP81Q1
CYP81Q2
CYP81Q3
CYP719A1
CYP719A2
CYP719A3
CYP719A4
CYP719A5
CYP719A13
CYP719A14
CYP719A21
CYP719A23
CYP719A24

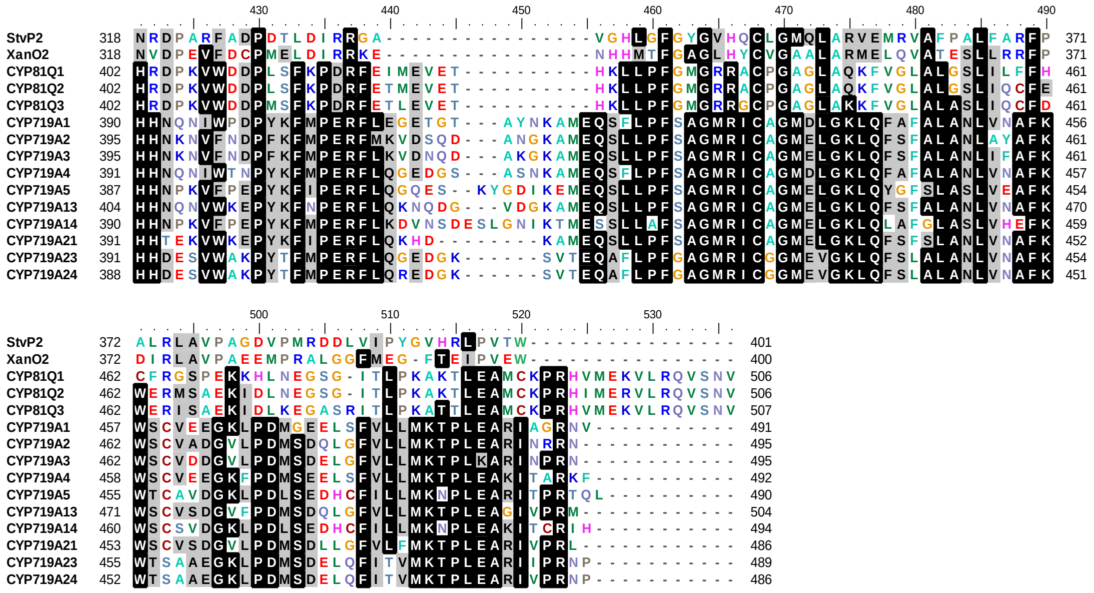

Supplementary Fig. 45 Amino acid sequence alignment of StvP2 with other cytochrome P450 enzymes reported to catalyze MDB formation. The amino acid residues of crucial catalytic triad in StvP2 protein are marked with the red asterisks and the amino acid sequences of other cytochrome P450 enzymes used for the analysis were obtained from GenBank showed in Supplementary Fig. 5. The sequence alignment is conducted with the software BioEdit (version: 7.2.6).

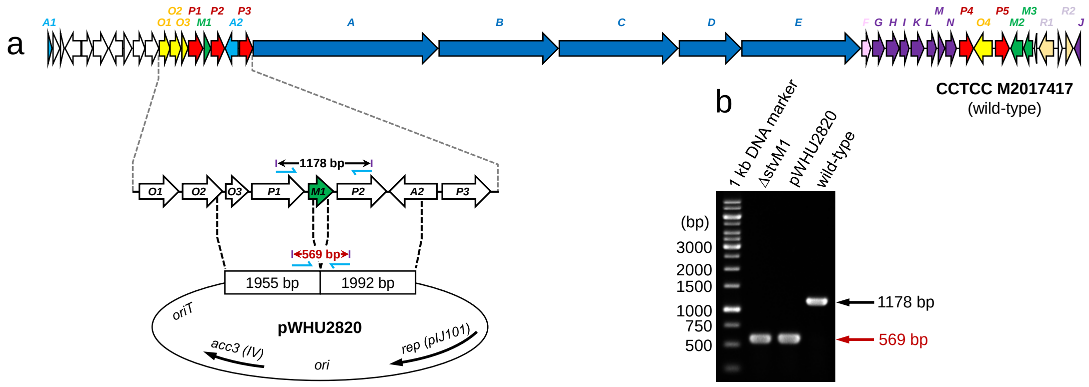

Supplementary Fig. 46 Scheme of mutant ΔstvM1 construction and mutant verification. (**a**) Representative scheme for in-frame deletion of *stvM1*. The primers used for checking the mutant is indicated by blue half arrows. (**b**) Agarose gel electrophoresis of mutant verification for ΔstvM1 by PCR. The result in **b** is representative of three independent experiments. Source data are provided as a Source Data file.

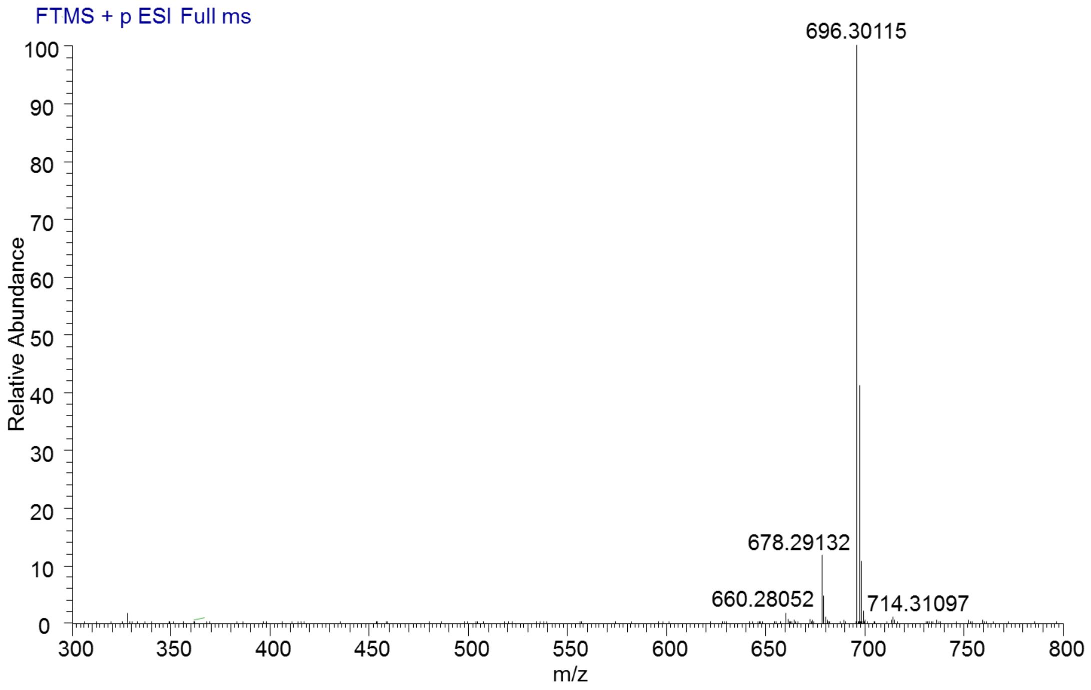

Supplementary Fig. 47 ESI-HRMS spectrum of damavaricin C (7).

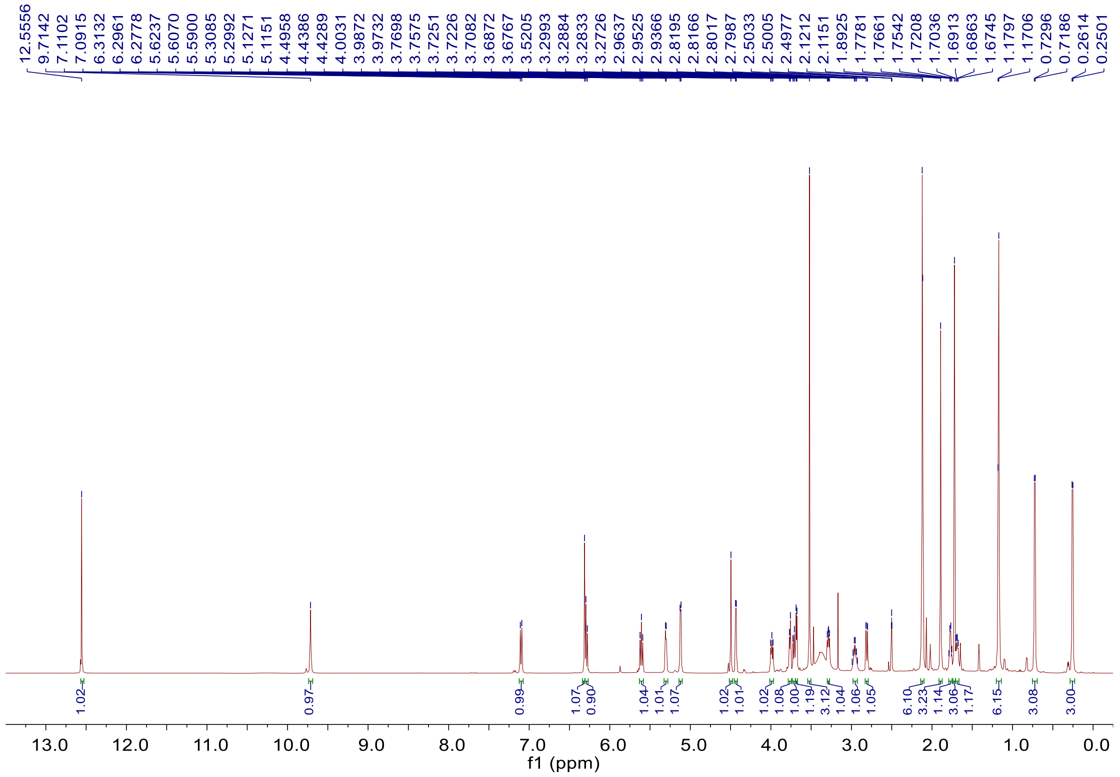

Supplementary Fig. 48 ^{1}H NMR spectrum (600 MHz, DMSO-d_6) of damavaricin C (7).

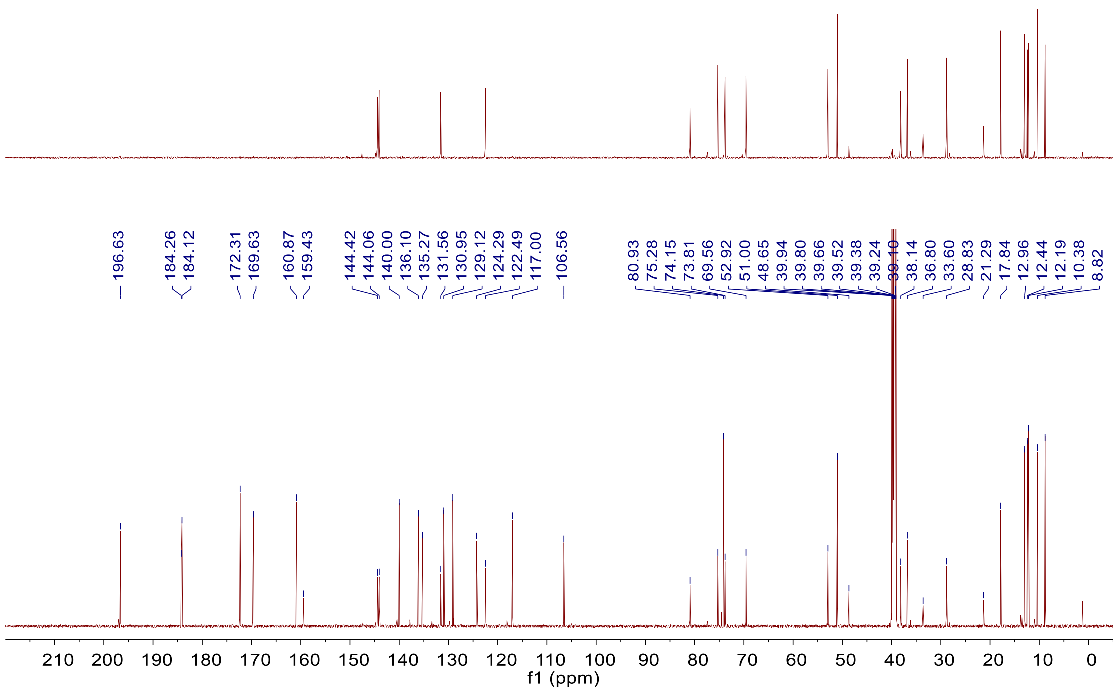

Supplementary Fig. 49 ^{13}C NMR and DEPT135 spectrum (150 MHz, DMSO-d_6) of damavaricin C (7).

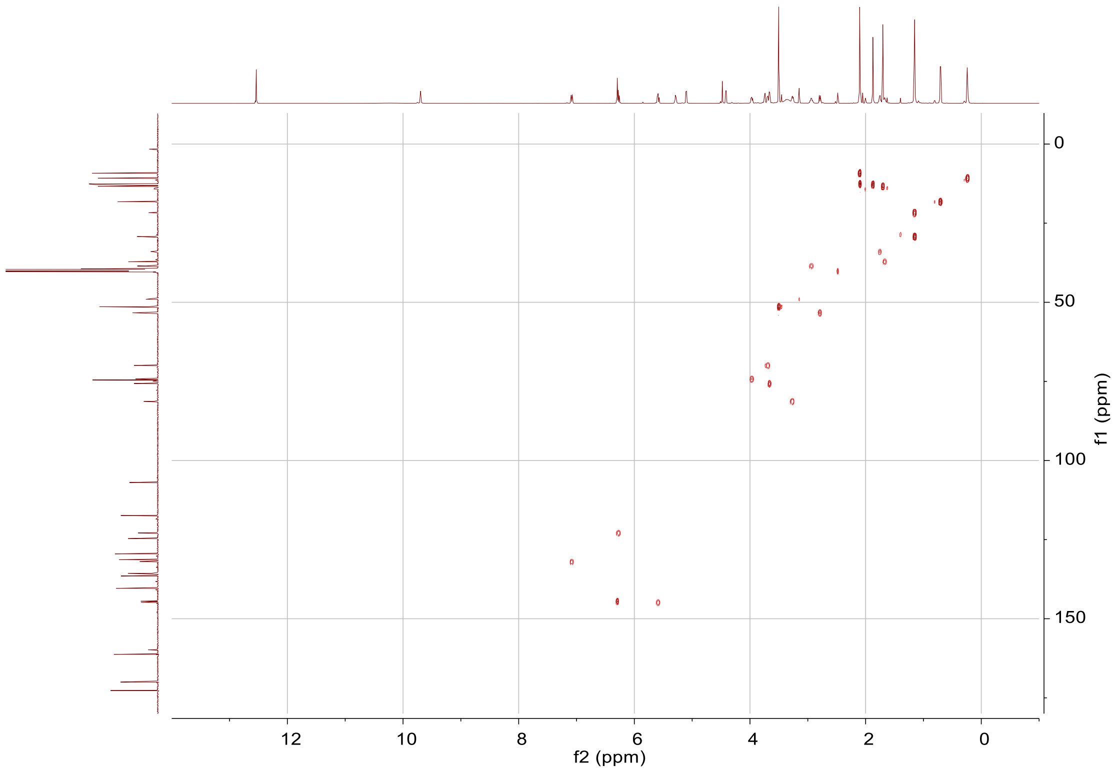

Supplementary Fig. 50 HSQC spectrum (600 MHz, DMSO-d_6) of damavaricin C (7).

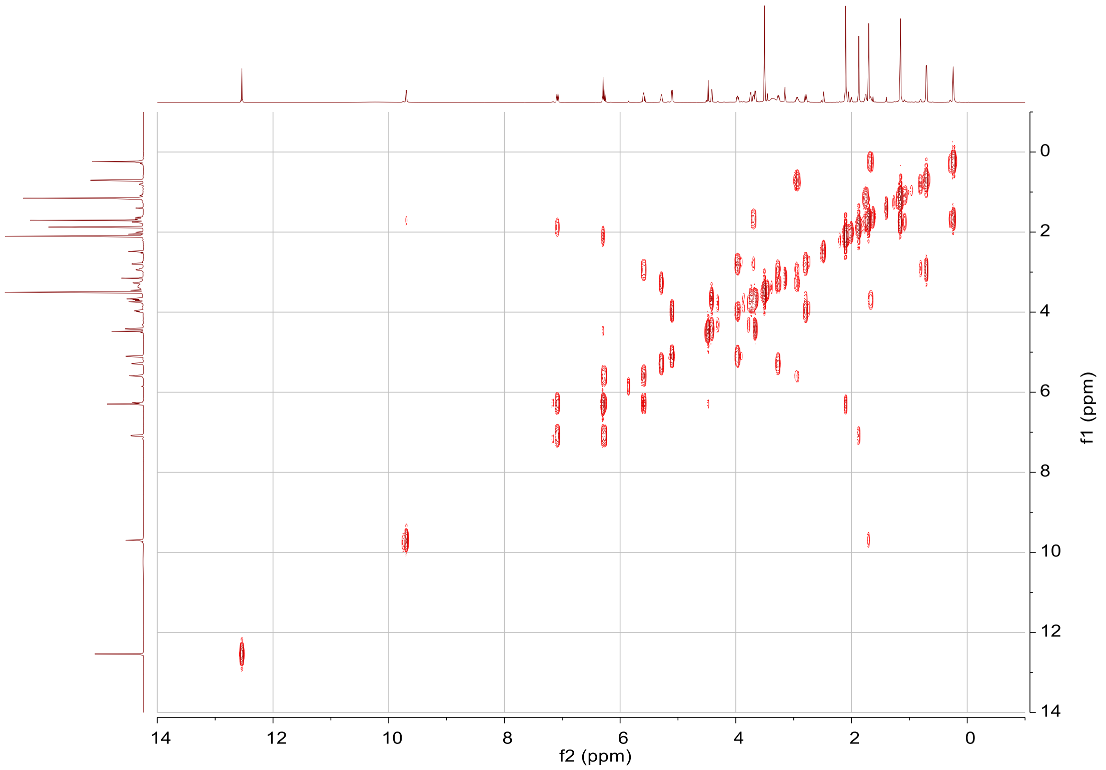

Supplementary Fig. 51 ^{1}H-^{1}H COSY spectrum (600 MHz, DMSO-d_6) of damavaricin C (7).

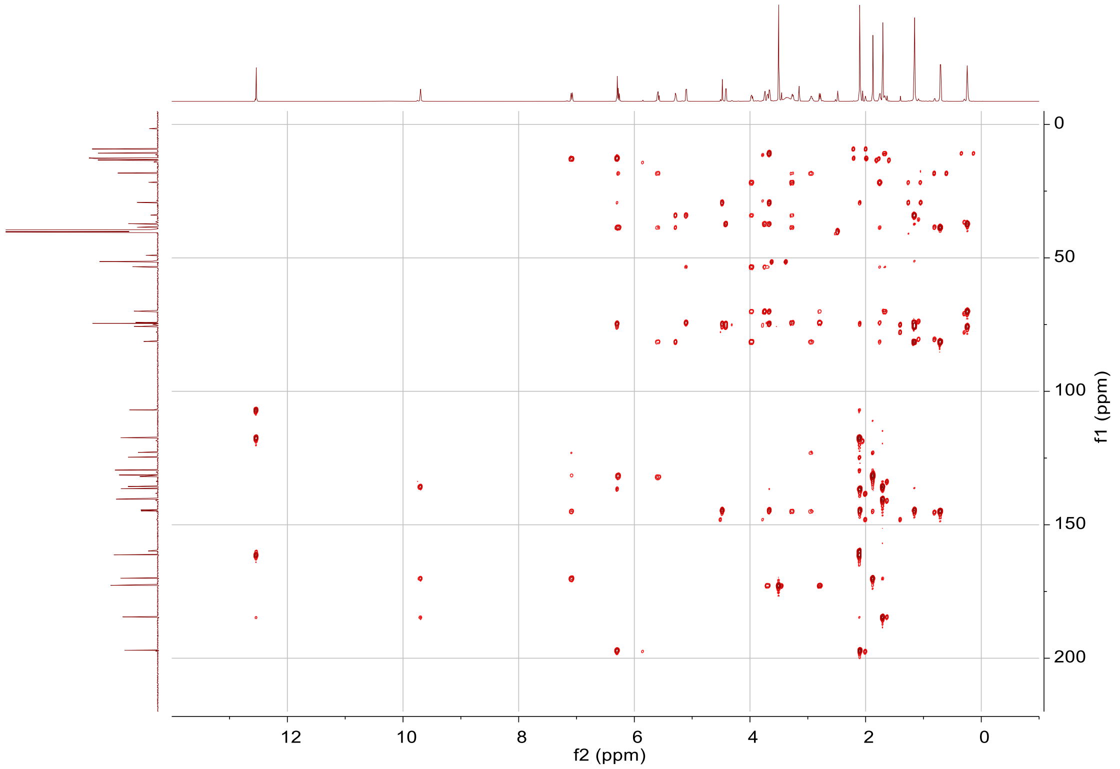

Supplementary Fig. 52 HMBC spectrum (600 MHz, DMSO-d_6) of damavaricin C (7).

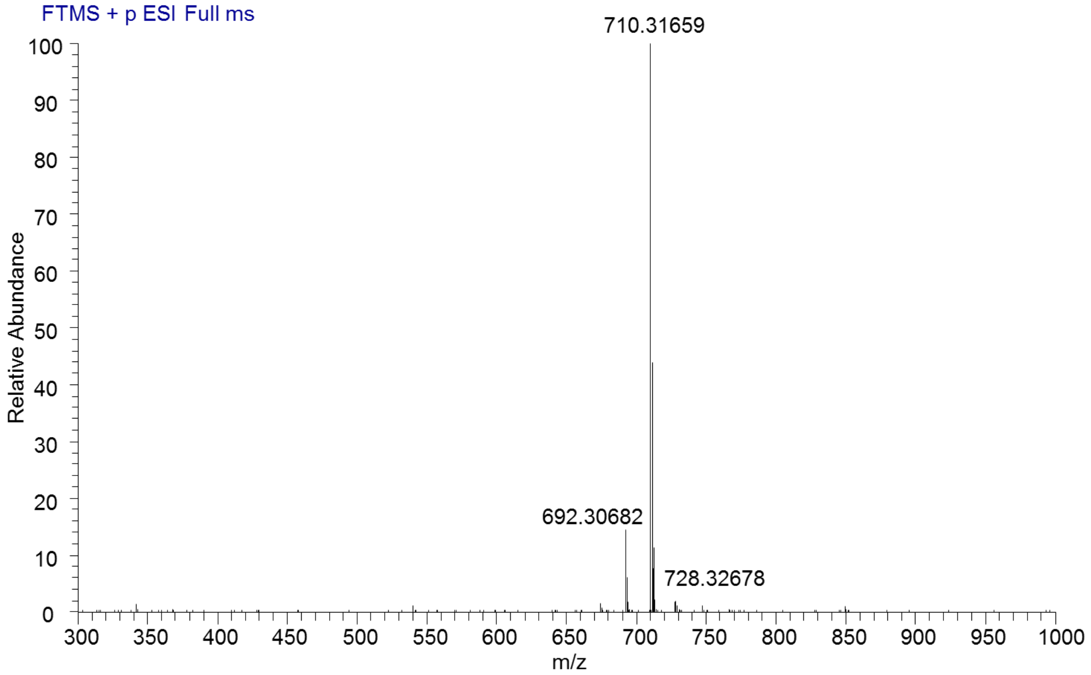

Supplementary Fig. 53 ESI-HRMS spectrum of 6-methoxy-damavaricin C (8).

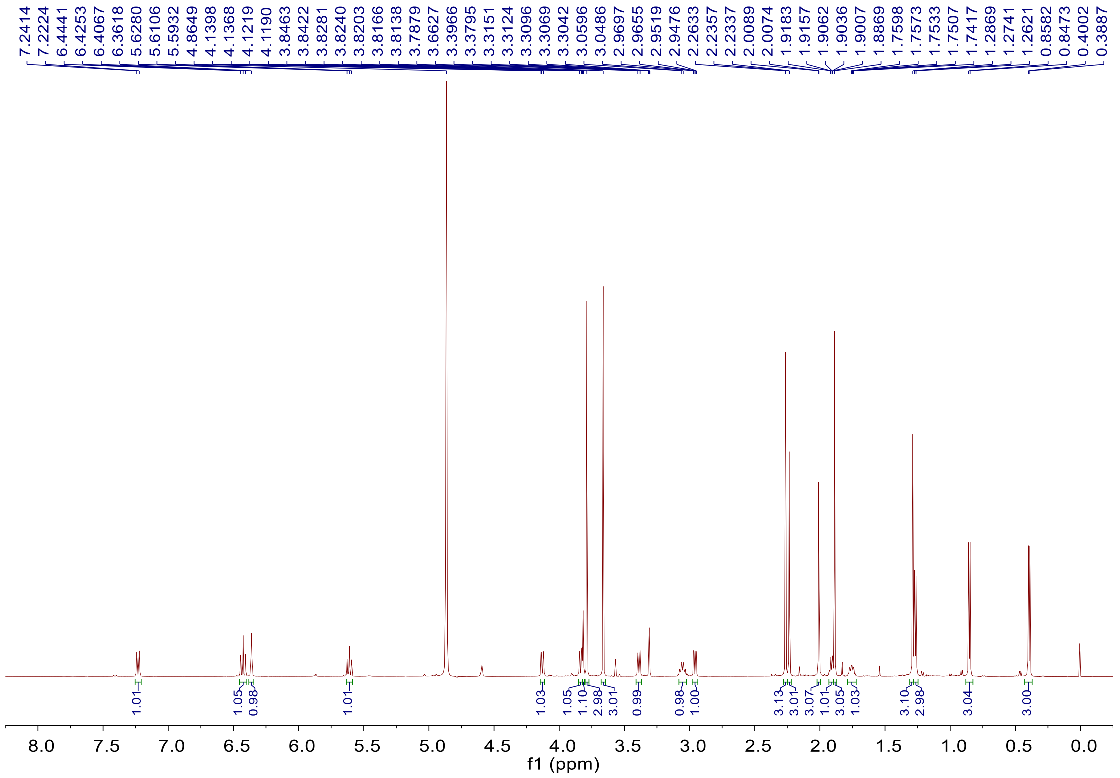

Supplementary Fig. 54 ^1H NMR spectrum (600 MHz, CH_3OH-d_4) of 6-methoxy-damavaricin C (8).

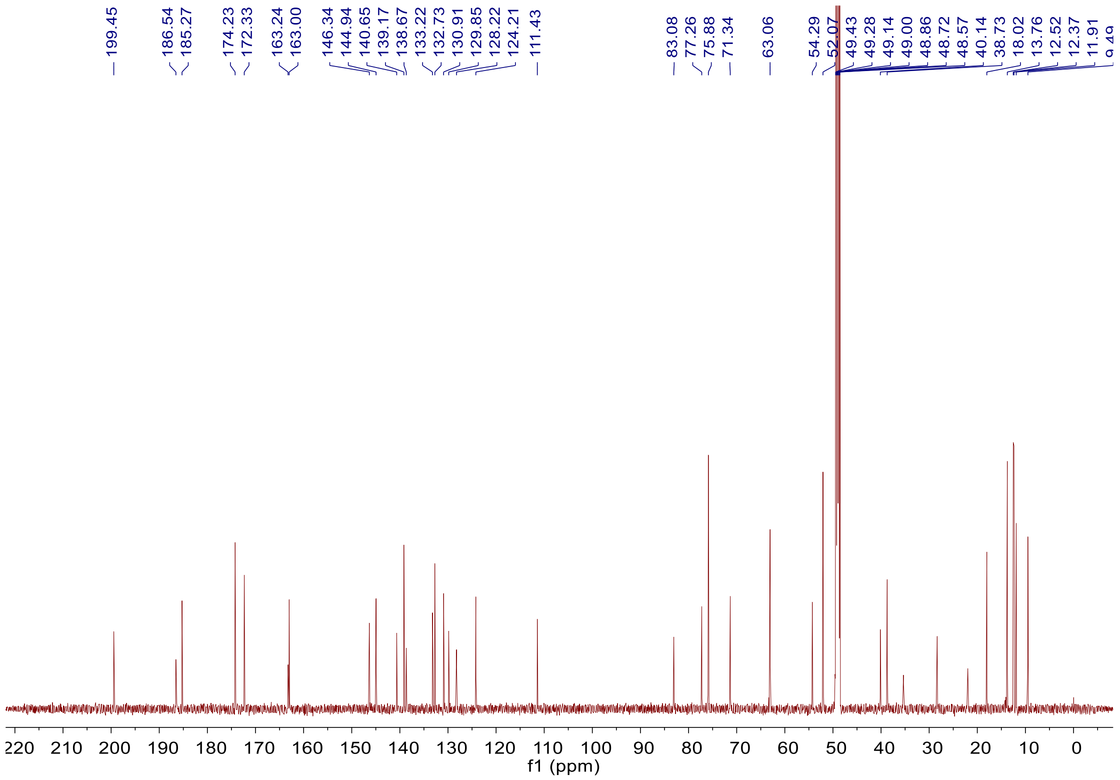

Supplementary Fig. 55 **^{13}C NMR spectrum (150 MHz, CH_3OH-*d*$_4$) of 6-methoxy-damavaricin C (8).**

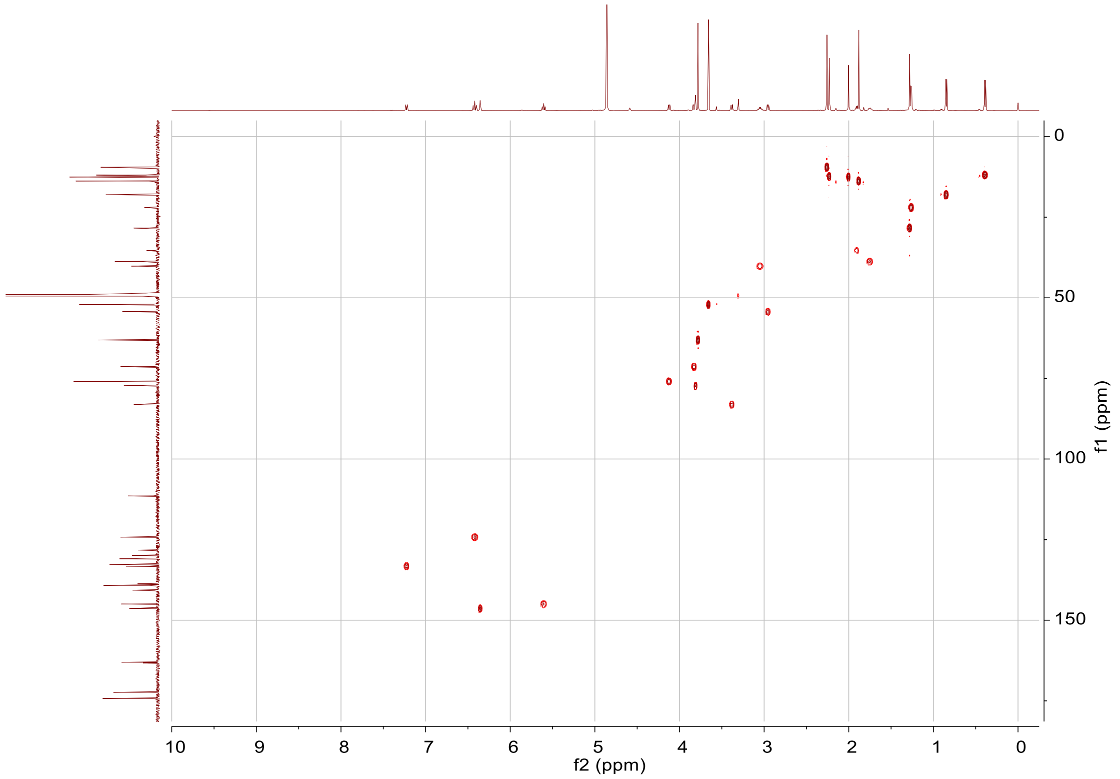

Supplementary Fig. 56 HSQC spectrum (600 MHz, CH_3OH-*d*$_4$) of 6-methoxy-damavaricin C (8).

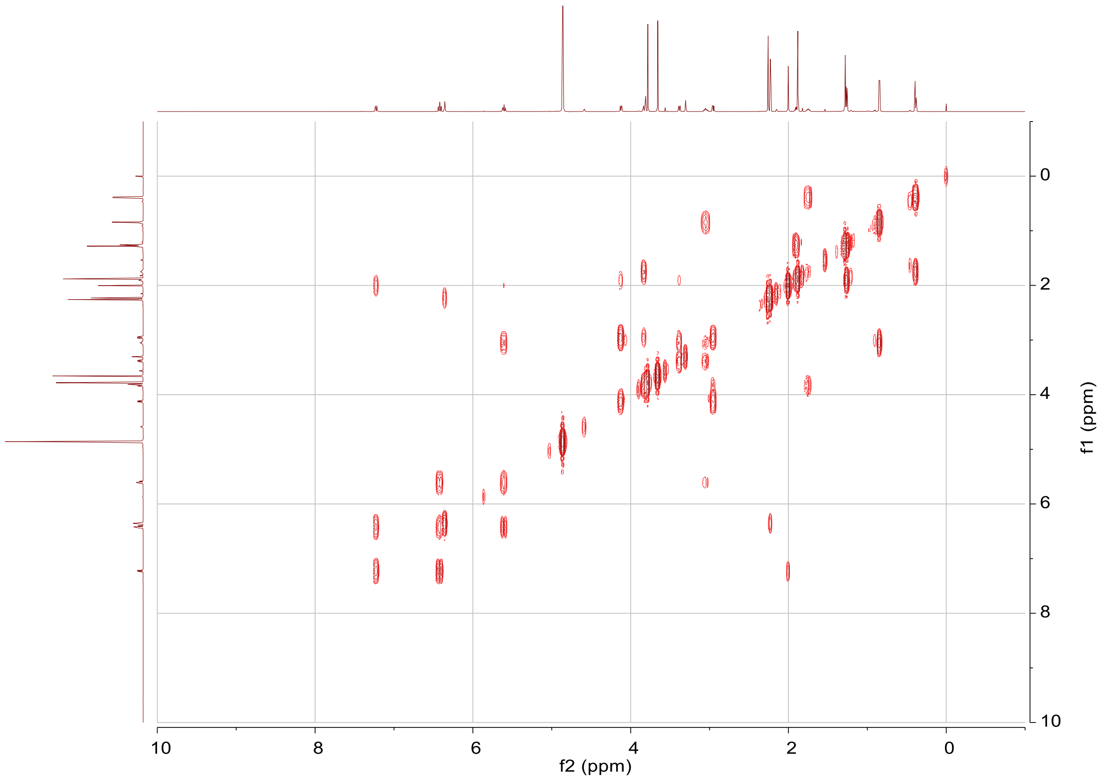

Supplementary Fig. 57 ^{1}H-^{1}H COSY spectrum (600 MHz, CH_3OH-d_4) of 6-methoxy-damavaricin C (8).

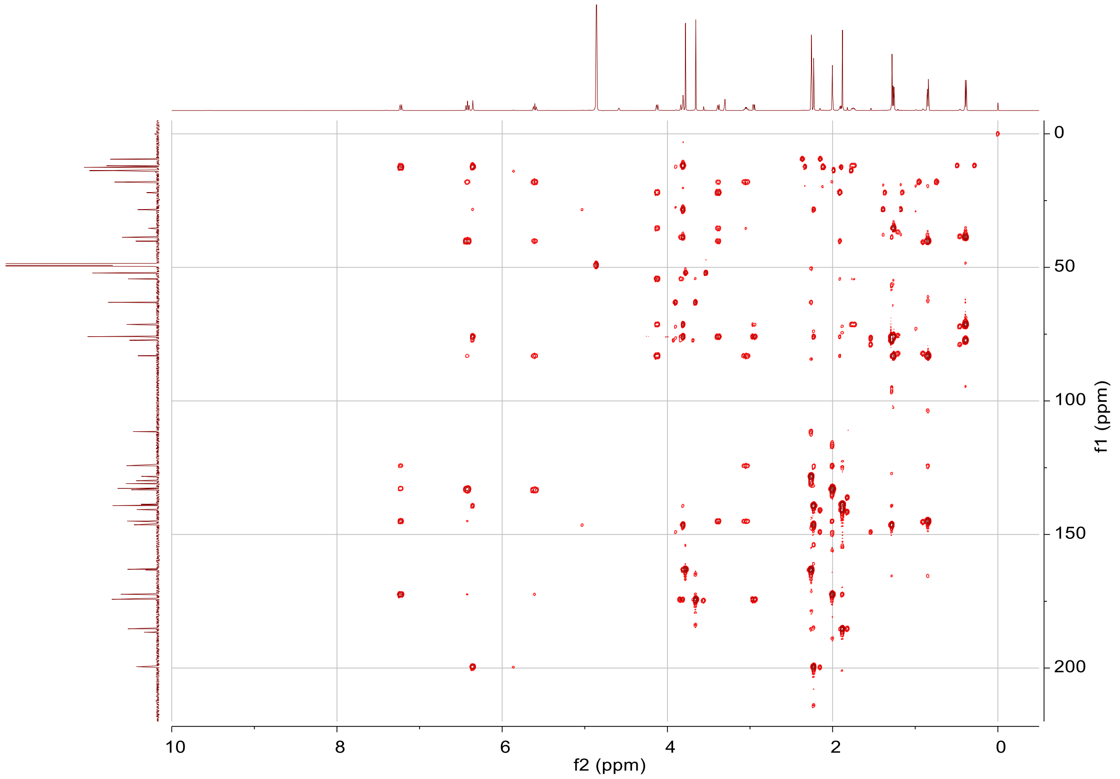

Supplementary Fig. 58 HMBC spectrum (600 MHz, CH_3OH-d_4) of 6-methoxy-damavaricin C (8).

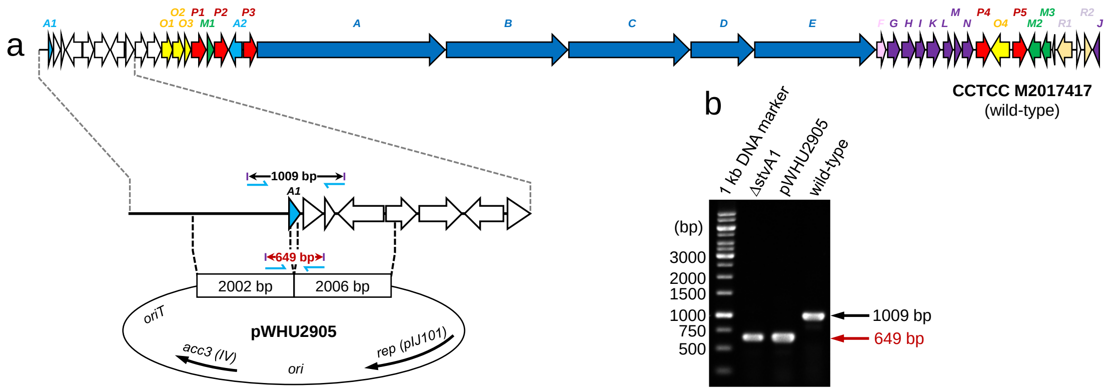

Supplementary Fig. 59 Scheme of mutant ΔstvA1 construction and mutant verification. (**a**) Representative scheme for in-frame deletion of *stvA1*. The primers used for checking the mutant is indicated by blue half arrows. (**b**) Agarose gel electrophoresis of mutant verification for ΔstvA1 by PCR. The result in **b** is representative of three independent experiments. Source data are provided as a Source Data file.

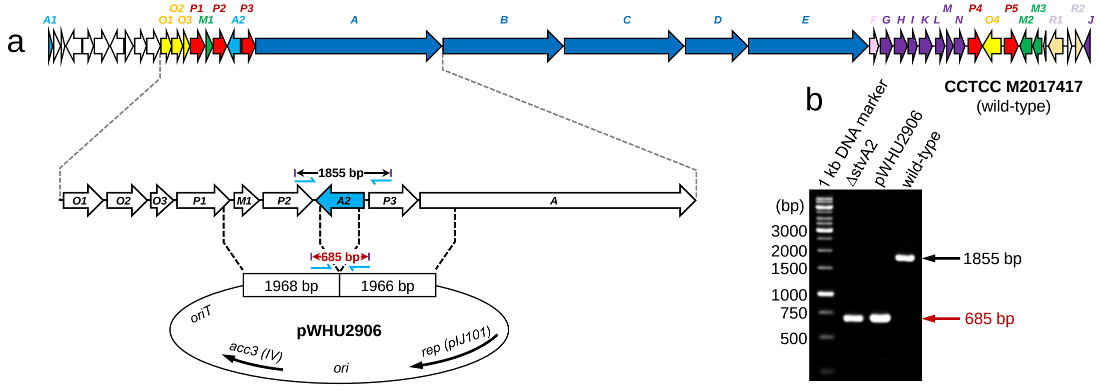

Supplementary Fig. 60 Scheme of mutant ΔstvA2 construction and mutant verification. (**a**) Representative scheme for in-frame deletion of *stvA2*. The primers used for checking the mutant is indicated by blue half arrows. (**b**) Agarose gel electrophoresis of mutant verification for ΔstvA2 by PCR. The result in **b** is representative of three independent experiments. Source data are provided as a Source Data file.

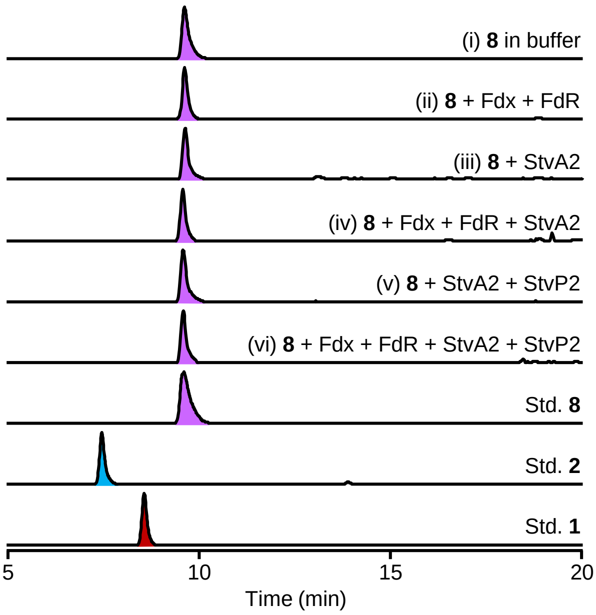

Supplementary Fig. 61 *In vitro* conversion of compound 8. (i) **8** in reaction buffer. (ii) **8** in reaction buffer with Fdx, FdR and NADPH. (iii) **8** and StvA2 in reaction buffer without Fdx, FdR and NADPH. (iv) **8** and StvA2 in reaction buffer with Fdx, FdR and NADPH. (v) **8**, StvA2 and StvP2 in reaction buffer without Fdx, FdR and NADPH. (vi) **8**, StvA2 and StvP2 in reaction buffer with Fdx, FdR and NADPH. All the enzymatic reactions were incubated at 28ºC for 3 h. Pure compounds (**1**, **2**, and **8**) identified by NMR were used as standards or substrate in assays. The LC-ESI-HRMS traces were extracted at calcd. *m/z* for $[M + H]^+$: 770.33823 (**1**, red peak), 772.35388 (**2**, blue peak), 728.32767 (**8**, purple peak), 814.36445 (the compound with double acetylations at C-4 and C-21 or C-25), 812.34880 (the MDB-contained compound with double acetylations at C-4 and C-21 or C-25). Experiments in (i)-(ix) are representative of three independent experiments.

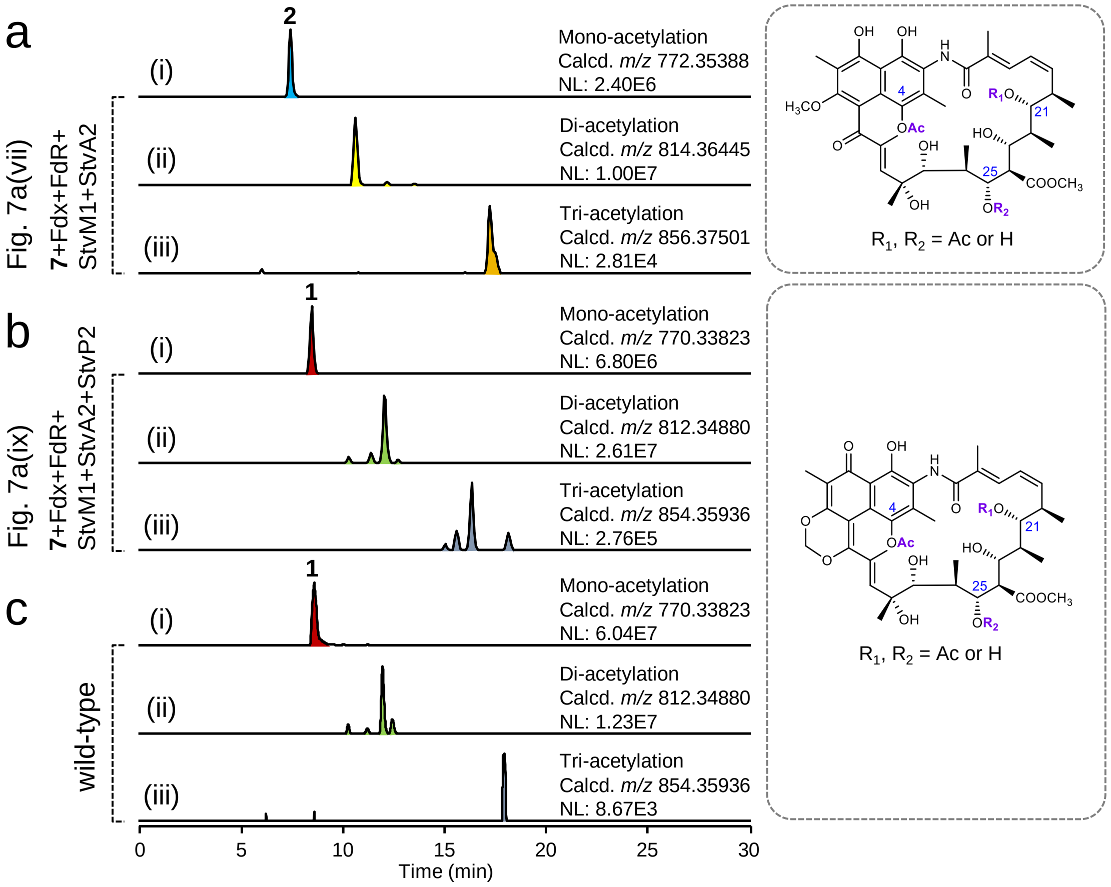

Supplementary Fig. 62 LC-ESI-HRMS analysis of acetylated products of enzymatic conversion of 7 by StvP2. (**a**) Mono-acetylated product (i), di-acetylated products (ii), tri-acetylated products (iii) from enzymatic conversion in Fig. 6a(vii) (**7**+Fdx+FdR+StvM1+StvA2) were extracted at calcd. *m/z* ([M+H]$^+$) 772.35388, 814.36445, 856.37501, respectively; (**b**) Mono-acetylated product (i), di-acetylated products (ii), tri-acetylated products (iii) from enzymatic conversion in Fig. 6a(ix) (**7**+Fdx+FdR+StvM1+StvA2+StvP2) were extracted at calcd. *m/z* ([M+H]$^+$) 770.33823, 812.34880, 854.35936, respectively. (**c**) Mono-acetylated product (i), di-acetylated products (ii), tri-acetylated products (iii) from the crude extracts of wild-type strain was extracted at calcd. *m/z* ([M+H]$^+$) 770.33823, 812.34880, 854.35936, respectively, and used as natural standards for determination of the acetylated products. Experiments in **a**-**c** are representative of three independent experiments.

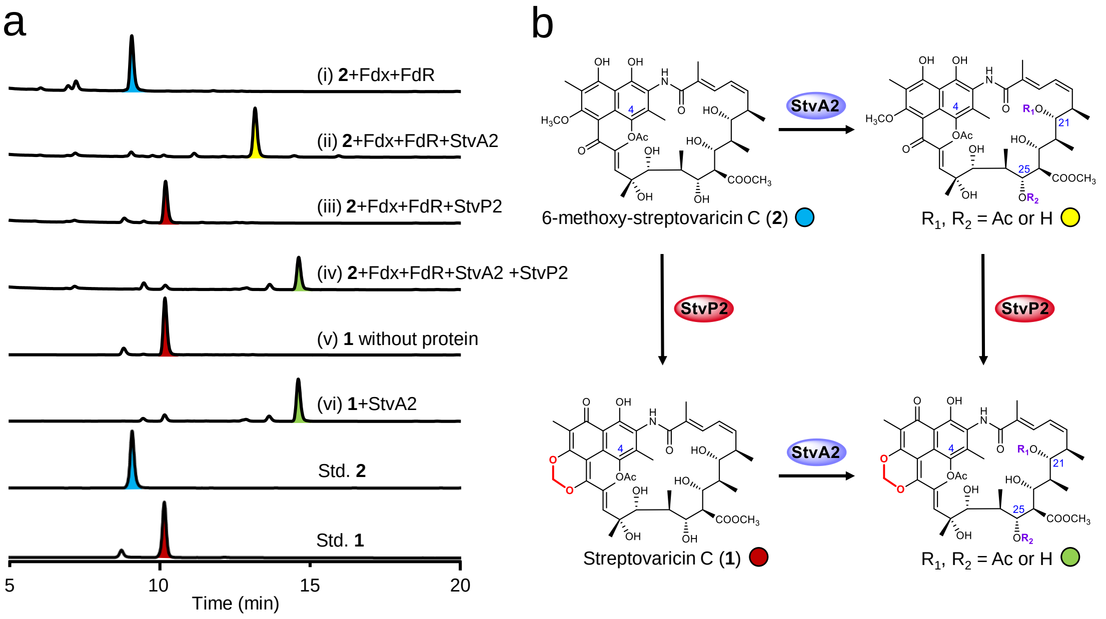

Supplementary Fig. 63 A biosynthetic network of acetylation and MDB formation confirmed by *in vitro* assay. (**a**) HPLC-DAD analysis at λ_{max}245 nm of enzymatic conversion by StvA2 and/or StvP2 using **2** as substrate. The pure compounds **1** and **2** identified by NMR were used as substrate in assay. The peak with the same retention time was labeled in the same color. (**b**) A proposed biosynthetic network of acetylation and MDB formation based on the assay results. Experiments in **a** are representative of three independent experiments.

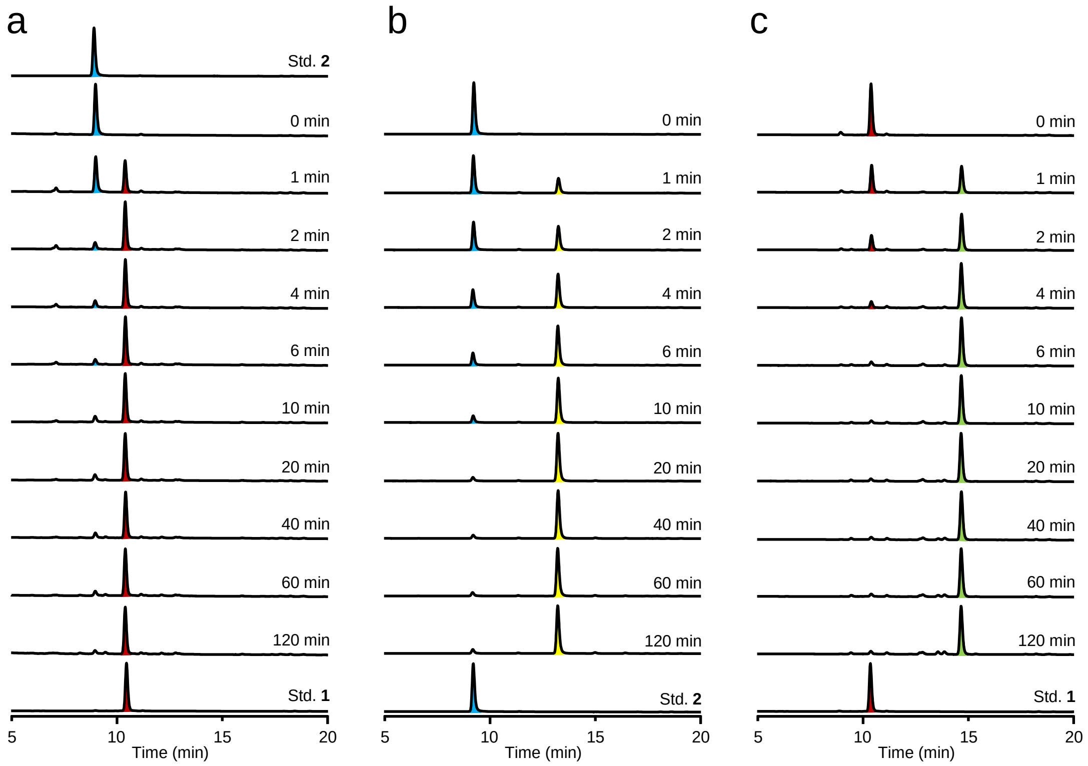

Supplementary Fig. 64 Comparative analysis of the catalytic efficiency of StvP2 and onStvA2. (**a**) Time dependent conversion of **2** to **1** by StvP2. (**b**) Time dependent conversion of **2** to 21 or 25-acetoxy-6-methoxy-streptovaricin C by StvA2. (**c**) Time dependent conversion of **1** to 21 or 25-acetoxy-streptovaricin C by StvA2. The conversions are terminated at 0 min, 1 min, 2 min, 4 min, 6 min, 10 min, 20 min, 40 min, 60 min, and 120 min, respectively, and analyzed by HPLC-DAD at the λ_{max} 245 nm. The pure compounds **1** and **2** identified by NMR are used as standards (Std. **1** and Std. **2**). The peaks with different color are representative of different components: **1** (blue), **2** (red), 21 or 25-acetoxy-6-methoxy-streptovaricin C (yellow), 21 or 25-acetoxy-streptovaricin C (green). All conversions are independently conducted three times with the similar results.

Supplementary References

1 Kakinuma, K., Milavetz, B. I. & Rinehart, K. L. Carbon-13 nuclear magnetic resonance spectra of the streptovaricins and related compounds. *J. Org. Chem.* **41**, 1358-1364 (1976).

2 Rinehart, K. L. et al. Identification and preparation of damavaricins, biologically active precursors of streptovaricins. *J. Antibiot.* **29**, 201-203 (1976).

3 Zhang, Z. et al. Ansavaricins A–E: five new streptovaricin derivatives from *Streptomyces* sp. S012. *RSC Adv.* **7**, 5684-5693, (2017).

4 MacNeil, D. J. et al. Analysis of *Streptomyces avermitilis* genes required for avermectin biosynthesis utilizing a novel integration vector. *Gene* **111**, 61-68 (1992).

5 Liu, Y. et al. Functional analysis of cytochrome P450s involved in streptovaricin biosynthesis and generation of anti-MRSA analogues. *ACS Chem. Biol.* **12**, 2589-2597 (2017).

6 Sun, Y., He, X., Liang, J., Zhou, X. & Deng, Z. Analysis of functions in plasmid pHZ1358 influencing its genetic and structural stability in *Streptomyces lividans* 1326. *Appl. Microbiol. Biotechnol.* **82**, 303-310 (2009).
