# Supplementary material for: Uncovering the cytochrome P450-catalyzed methylenedioxy bridge formation in streptovaricins biosynthesis
Source: Nat Commun. 2020 Sep 9;11:4501. doi: 10.1038/s41467-020-18336-5 (PMC7481197; doi:10.1038/s41467-020-18336-5)
Supplement: Supplementary file 3 — Reporting Summary [file 41467_2020_18336_MOESM3_ESM.pdf]

nature research

Corresponding author(s): Yuhui Sun and Zhengyu Zhang

Last updated by author(s): Aug 13, 2020

Reporting Summary

Nature Research wishes to improve the reproducibility of the work that we publish. This form provides structure for consistency and transparency in reporting. For further information on Nature Research policies, see our Editorial Policies and the Editorial Policy Checklist.

Statistics

For all statistical analyses, confirm that the following items are present in the figure legend, table legend, main text, or Methods section.

n/a	Confirmed	
☐	☒	The exact sample size (n) for each experimental group/condition, given as a discrete number and unit of measurement
☐	☒	A statement on whether measurements were taken from distinct samples or whether the same sample was measured repeatedly
☒	☐	The statistical test(s) used AND whether they are one- or two-sided<br>*Only common tests should be described solely by name; describe more complex techniques in the Methods section.*
☒	☐	A description of all covariates tested
☒	☐	A description of any assumptions or corrections, such as tests of normality and adjustment for multiple comparisons
☒	☐	A full description of the statistical parameters including central tendency (e.g. means) or other basic estimates (e.g. regression coefficient) AND variation (e.g. standard deviation) or associated estimates of uncertainty (e.g. confidence intervals)
☒	☐	For null hypothesis testing, the test statistic (e.g. F, t, r) with confidence intervals, effect sizes, degrees of freedom and P value noted<br>*Give P values as exact values whenever suitable.*
☒	☐	For Bayesian analysis, information on the choice of priors and Markov chain Monte Carlo settings
☒	☐	For hierarchical and complex designs, identification of the appropriate level for tests and full reporting of outcomes
☒	☐	Estimates of effect sizes (e.g. Cohen's d, Pearson's r), indicating how they were calculated

Our web collection on statistics for biologists contains articles on many of the points above.

Software and code

Policy information about availability of computer code

Data collection	The crystallography data were collected at SSRF, China Shanghai. No software was used for data collection.
Data analysis	CPP4i Version 7.0<br>Coot Version: 0.8.1<br>Phenix Version 1.18<br>Pymol Vesion 1.82<br>MEGA Version: 6.0<br>BioEdit Version: 7.2.6<br>OriginPro 2017C Version: SR2 b9.4.2.380<br>MestReNova Version: 9.0.1-13254<br>ChemBioDraw Version: 14.0.0.117

For manuscripts utilizing custom algorithms or software that are central to the research but not yet described in published literature, software must be made available to editors and reviewers. We strongly encourage code deposition in a community repository (e.g. GitHub). See the Nature Research guidelines for submitting code & software for further information.

Data

Policy information about availability of data

All manuscripts must include a data availability statement. This statement should provide the following information, where applicable:

- Accession codes, unique identifiers, or web links for publicly available datasets
- A list of figures that have associated raw data
- A description of any restrictions on data availability

The X-ray structures of StvP2 alone or in complex with its substrate were deposited in the Protein Data Bank under accession codes 6M4Q [https://www.rcsb.org/structure/6M4Q or 10.2210/pdb6M4Q/pdb] and 6M4P [https://www.rcsb.org/structure/6M4P or 10.2210/pdb6M4P/pdb]. The source data for Fig. 3, Supplementary Table 4, and Supplementary Figs. 6, 15, 16, 46, 59, 60 are provided with this paper. All other data generated and analyzed in this study are available within the article and the Supplementary Information.

Field-specific reporting

Please select the one below that is the best fit for your research. If you are not sure, read the appropriate sections before making your selection.

[x] Life sciences [ ] Behavioural & social sciences [ ] Ecological, evolutionary & environmental sciences

For a reference copy of the document with all sections, see nature.com/documents/nr-reporting-summary-flat.pdf

Life sciences study design

All studies must disclose on these points even when the disclosure is negative.

Sample size	In this study, the kinetics analyses were determined with the sample of n=3.
Data exclusions	No data were excluded.
Replication	All biochemical assays and the product detection of Streptomycete mutants were repeated in triplicate with the similar results.
Randomization	The data represented in this manuscript are the biochemical assays on enzyme function and reconstitution of the biosynthetic pathway, which require a rational approach to data collection and analysis. Therefore, randomization is not applicable to our experimental set up.
Blinding	Blinding is not applicable to any biochemical assay performed in this study due to the need for rational design.

Reporting for specific materials, systems and methods

We require information from authors about some types of materials, experimental systems and methods used in many studies. Here, indicate whether each material, system or method listed is relevant to your study. If you are not sure if a list item applies to your research, read the appropriate section before selecting a response.

Materials & experimental systems

n/a	Involved in the study	
[x]	[ ]	Antibodies
[x]	[ ]	Eukaryotic cell lines
[x]	[ ]	Palaeontology and archaeology
[x]	[ ]	Animals and other organisms
[x]	[ ]	Human research participants
[x]	[ ]	Clinical data
[x]	[ ]	Dual use research of concern

Methods

n/a	Involved in the study	
[x]	[ ]	ChIP-seq
[x]	[ ]	Flow cytometry
[x]	[ ]	MRI-based neuroimaging
